# Infant rhesus macaques immunized against SARS-CoV-2 are protected against heterologous virus challenge one year later

**DOI:** 10.1126/scitranslmed.add6383

**Published:** 2022-12-01

**Authors:** Emma C. Milligan, Katherine Olstad, Caitlin A. Williams, Michael Mallory, Patricio Cano, Kaitlyn A. Cross, Jennifer E. Munt, Carolina Garrido, Lisa Lindesmith, Jennifer Watanabe, Jodie L. Usachenko, Lincoln Hopkins, Ramya Immareddy, Yashavanth Shaan Lakshmanappa, Sonny R. Elizaldi, Jamin W. Roh, Rebecca L. Sammak, Rachel E. Pollard, JoAnn L. Yee, Savannah Herbek, Trevor Scobey, Dieter Miehlke, Genevieve Fouda, Guido Ferrari, Hongmei Gao, Xiaoying Shen, Pamela A. Kozlowski, David Montefiori, Michael G. Hudgens, Darin K. Edwards, Andrea Carfi, Kizzmekia S. Corbett, Barney S. Graham, Christopher B. Fox, Mark Tomai, Smita S. Iyer, Ralph Baric, Rachel Reader, Dirk P. Dittmer, Koen K.A. Van Rompay, Sallie R. Permar, Kristina De Paris

**Affiliations:** ^1^ Department of Microbiology and Immunology, Children’s Research Institute, School of Medicine, University of North Carolina at Chapel Hill, Chapel Hill, North Carolina 27599, USA.; ^2^ California National Primate Research Center, University of California at Davis, Davis, California 95616, USA.; ^3^ Department of Pediatrics, Weill Cornell Medical College, NY, New York 10065, USA.; ^4^ Department of Epidemiology, Gillings School of Global Public Health, University of North Carolina at Chapel Hill, Chapel Hill, North Carolina 27599, USA.; ^5^ Lineberger Cancer Center, University of North Carolina at Chapel Hill, Chapel Hill, North Carolina 27599, USA.; ^6^ Department of Biostatistics, Gillings School of Global Public Health, University of North Carolina at Chapel Hill, 27599 Chapel Hill, NC, USA.; ^7^ Center for Immunology and Infectious Diseases, University of California at Davis, Davis, California 95616, USA.; ^8^ Graduate Group in Immunology, University of California, Davis, California 95616, USA.; ^9^ School of Veterinary Medicine, University of California, Davis, California 95616, USA.; ^10^ Duke Human Vaccine Institute, Duke University Medical Center, Durham, North Carolina 27710, USA.; ^11^ Departent of Surgery, Duke University School of Medicine, Durham, North Carolina 27710, USA.; ^12^ Department of Molecular Genetics and Microbiology, Duke University Medical Center, Durham, North Carolina 27710, USA.; ^13^ Department of Microbiology, Immunology and Parasitology, Louisiana State University Health Sciences Center, New Orleans, Louisiana 70112, USA.; ^14^ Moderna, Inc., Cambridge, MA 02139, USA.; ^15^ Department of Immunology and Infectious Diseases, Harvard T.H. Chan School of Public Health, Boston, MA 02115, USA.; ^16^ Vaccine Research Center, National Institute of Allergy and Infectious Diseases, National Institutes of Health, Bethesda, MD 20852, USA.; ^17^ Access to Advanced Health Institute, Seattle, Washington 98102, USA.; ^18^ Department of Global Health, University of Washington, Seattle, Washington 98105, USA.; ^19^ 3M Corporate Research Materials Laboratory, Saint Paul, MN 55144, USA.; ^20^ Department of Pathology, Microbiology and Immunology, University of California at Davis, Davis, California, USA.

## Abstract

The U.S. Food and Drug Administration only gave emergency-use-authorization of the BNT162b2 and the mRNA-1273 SARS-CoV-2 vaccines for infants 6 months and older in June 2022. Yet, questions regarding the durability of vaccine efficacy, especially against emerging variants, in this age group remain. We demonstrated previously that a two-dose regimen of stabilized prefusion Washington SARS-CoV-2 S-2P spike (S) protein encoded by mRNA encapsulated in lipid nanoparticles (mRNA-LNP) or purified S-2P mixed with 3 M-052, a synthetic toll-like receptor (TLR) 7/8 agonist, in a squalene emulsion (Protein+3 M-052-SE) was safe and immunogenic in infant rhesus macaques. Here, we demonstrate that broadly neutralizing and spike-binding antibodies against variants of concern (VOC), as well as T cell responses, persisted for 12 months. At one year, corresponding to human toddler age, we challenged vaccinated rhesus macaques and age-matched non-vaccinated controls intranasally and intratracheally with a high-dose of heterologous SARS-CoV-2 B.1.617.2 (Delta). Seven of eight control rhesus macaques exhibited severe interstitial pneumonia and high virus replication in the upper and lower respiratory tract. In contrast, vaccinated rhesus macaques had faster viral clearance with mild to no pneumonia. Neutralizing and binding antibody responses to the B.1.617.2 variant at the day of challenge correlated with lung pathology and reduced virus replication. Overall, the Protein+3 M-052-SE vaccine provided superior protection to the mRNA-LNP vaccine, emphasizing opportunities for optimization of current vaccine platforms. Notably, the observed efficacy of both vaccines one year after vaccination supports the implementation of an early life SARS-CoV-2 vaccine.

## Teaser

SARS-CoV-2 vaccines protected infant rhesus macaques against severe lung disease after high dose challenge one year after vaccination.

## INTRODUCTION

Severe acute respiratory syndrome coronavirus 2 (SARS-CoV-2) has infected close to 600 million people and caused more than 6 million deaths worldwide (*1*). Despite available vaccines, this pandemic virus continues to spread globally posing a long-lasting burden. Fewer than 70% have received the full two-dose regimen worldwide and even fewer have received booster immunizations (*1*). Until June 2022, children under the age of 5 were excluded from vaccination due to stringent age de-escalation clinical trial protocols and early data from the pandemic suggesting that children become less frequently infected with SARS-CoV-2 than adults and experience only mild disease (*2*–*4*). Nonetheless, children can develop severe disease along with other complications, notably multisystem inflammatory syndrome (MIS-C), and require hospitalization (*5*). In the US alone, there have been more than 1500 reported deaths in children due to SARS-CoV-2 infection (*6*). Furthermore, with the emergence of more transmissible SARS-CoV-2 variants, such as B.1.617.2 (Delta) and B.1.1.529 (Omicron), relative disease incidence increased in the pediatric population, with neonates being one of the most affected age groups requiring hospitalization (*7*), emphasizing the urgent need to implement effective vaccines for this age group (*8*, *9*). Controversy remains as to whether young infants and children mount immune responses to SARS-CoV-2 infection comparable to those observed in adults (*10*–*13*). Recent data from phase 2/III studies in children ages 6 months and older for Moderna’s mRNA-1273 and Pfizer-BioNTech’s BNT162b vaccines, however, demonstrate the immunogenicity and efficacy of mRNA SARS-CoV-2 vaccines despite substantially lower doses than those used in adults (*14*, *15*), leading to recent emergency-use-authorization (EUA) of both of these vaccines for infants 6 months and older.

In human adults, vaccine-induced neutralizing antibody (nAb) responses against SARS-CoV-2 decline over time (*16*–*18*). Several studies have attempted to model the kinetics of antibody decline to inform timing of booster immunization and predict protective efficacy (*19*–*21*). Pediatric SARS-CoV-2 vaccine doses for infants between the ages of 6 months to 5 years will be scaled proportionally to that of adults (*14*, *15*) and, thus, it will be important to determine whether vaccine-induced antibody responses are durable and of sufficient breadth to protect against infection with new variants of concern (VOCs). The importance of this question is further underlined by the fact that booster immunizations in children aged 5 years and older were only approved in May 2022, and – to ensure an acceptable benefit to risk ratio – a similar delay can be expected for booster vaccines in the youngest age group.

Several groups have demonstrated the relevance of SARS-CoV-2 nonhuman primate (NHP) models predictive of vaccine-mediated protection and disease outcomes (*22*–*30*). We previously reported that infant rhesus macaques (RMs) vaccinated at 2 months of age with two doses of an adjuvanted S-2P Protein+3 M-052-SE vaccine or a preclinical version of the Moderna SARS-CoV-2 mRNA-LNP vaccine mounted potent antibody and T cell responses that persisted for 22 weeks and that, despite lower doses, were comparable to those induced by the same vaccines in adult RMs (*28*). The objective of the current study was to provide proof-of-concept that immune responses induced by early life vaccination would persist and protect from severe disease after high-dose challenge with a heterologous variant one year after vaccination. Our results suggest that the decay kinetics of vaccine-induced neutralizing antibody responses are comparable to those observed in adult humans and NHPs. Even at one year after immunization, an age corresponding to human toddlers, RMs vaccinated as infants had reduced virus replication and were protected against SARS-CoV-2-induced severe lung inflammation.

## RESULTS

### Study Design

**Fig. 1.**
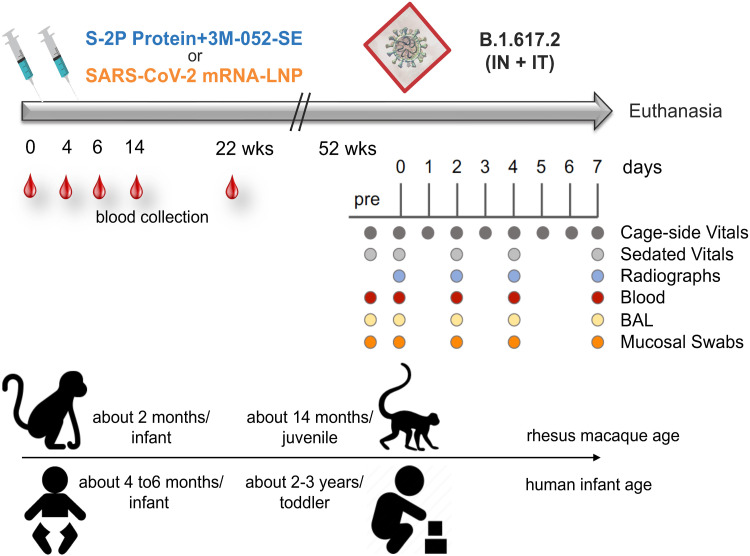
Experimental Design. Two groups of 2-month old rhesus macaques (RMs) were immunized intramuscularly at weeks (wks) 0 and 4 with stabilized prefusion SARS-CoV-2 S-2P spike (S) protein of the Washington (SARS-CoV-2/human/USA/WA-CDC-02982586-001/2020) strain encoded by mRNA encapsulated in lipid nanoparticles (mRNA-LNP; n = 8) or purified S protein mixed with 3 M-052, a synthetic TLR7/8 agonist, in a squalene emulsion (Protein+3 M-052-SE; n = 8). Immunogenicity data up to week 22 post-immunization have been previously reported (*28*). RMs were monitored for 1 year post immunization, when vaccinated and an additional group of age-matched unvaccinated control RMs were challenged by the intranasal (IN) and intratracheal (IT) route with heterologous B.1.617.2 (Delta). The clinical monitoring and sample collection schedule until euthanasia on day 7 post-challenge is indicated underneath the time bar. The arrow at the bottom of the figure indicates the approximate age of human infants at the time of the first immunization of RMs and at the time of challenge. The age comparison is based on our data of normal changes in immune parameters in human and RM infants (*32*–*34*, *65*).

We immunized two groups of 2-month old RMs (n = 8 per group) at weeks 0 and 4 intramuscularly (IM) with stabilized prefusion SARS-CoV-2 S-2P spike (S) protein of the Washington strain encoded by mRNA encapsulated in lipid nanoparticles (mRNA group; Fig. 1; table S1) or purified S protein mixed with 3 M-052, a synthetic toll-like receptor (TLR) 7/8 agonist, in a squalene emulsion (Protein group; Fig. 1; table S1) (*28*). The current study expanded on the previously published findings of vaccine-induced immune responses out to week 22 (*28*) and assessed the persistence of antibody and T cell responses to the vaccine virus and to VOCs for up to 12 months. To determine vaccine efficacy, at one year after the initial immunization, the 2 vaccine groups and an added control group of 8 age-matched non-immunized RMs (table S1) were exposed to a high-dose heterologous challenge with the B.1.617.2 (Delta) variant, administered by the intratracheal [IT; 2x10^6^ plaque-forming units (pfu)] and intranasal (IN; 1x10^6^ pfu) routes. After challenge, animals were monitored closely by clinical observations and regular sample collection until day 7 when animals were euthanized for tissue collection (Fig. 1). Day 7 was selected for euthanasia, because prior studies in adult RMs had demonstrated that even with minimal or mild overt clinical signs, at this time point there is substantial gross and histological evidence of lung inflammation, particularly interstitial pneumonia, which subsides later (*30*, *31*). Based on findings of normal developmental changes in infant immune parameters by us and others (*32*–*35*) and the fact that RMs have a 3 to 4-fold reduced lifespan to humans (*36*), we estimate that the RM age at the time of immunization or challenge corresponded to approximately 6-month old human infants or 3.5 year old toddlers, respectively.

### Vaccine-induced immune responses persisted in infant RMs for one year after immunization

**Fig. 2.**
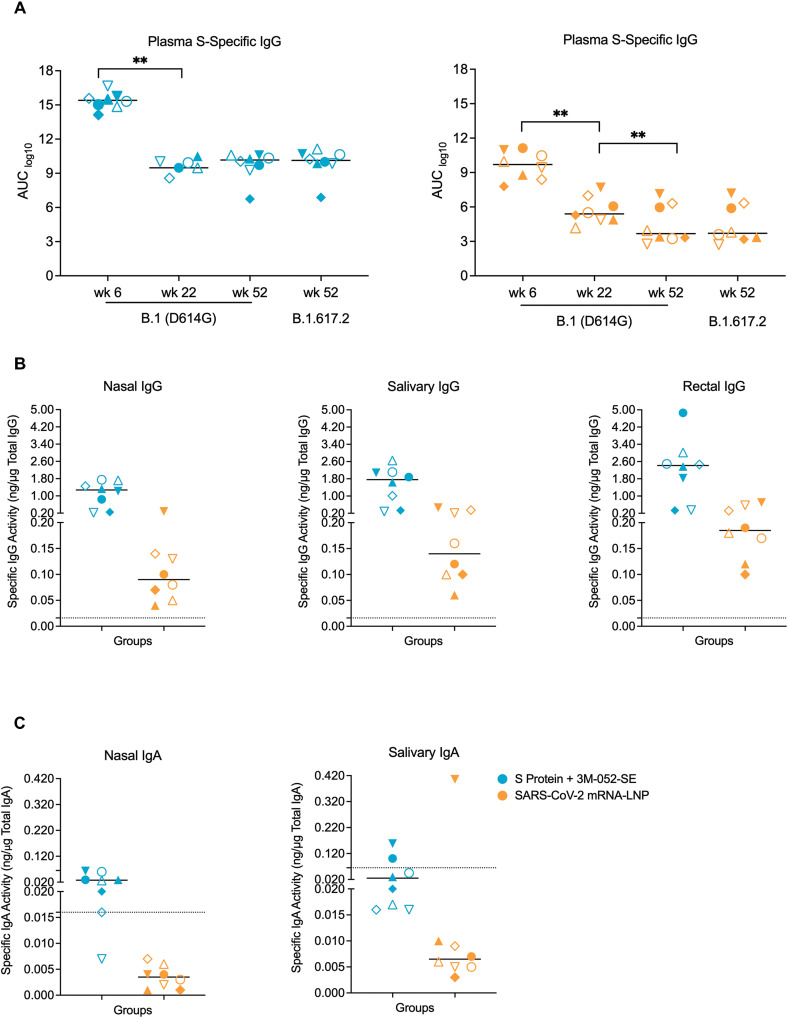
mRNA and protein vaccines induced SARS-CoV-2-specific antibody responses in infant RMs. (**A**) Week 6, 22, and 52 plasma IgG responses to the B.1 (D614G) or B.1.617.2 S protein (wk 52 only) are reported as log_10_ area-under-the curve (AUC) for RMs in the Protein+3 M-052-SE (blue symbols; left panel) or mRNA (orange symbols, right panel). (**B**) Specific IgG activity to B.1.617.2 RBD was measured in nasal, salivary and rectal secretions of vaccinated RMs after 1 year. (**C**) Specific IgA activity to B.1.617.2 RBD was measured in nasal and salivary secretions of the vaccinated RMs at 1 year. Each symbol represents an individual animal (table S1) with n = 8 RMs per group. Group medians are denoted by bars in all graphs. Dotted lines in Panels B and C represent the cut-off for significance (mean + 3SD of negative controls) in the assays. Within group comparisons (A) and between group comparisons (B and C) were respectively performed using Wilcoxon signed rank test and Mann-Whitney rank sum test. *p < 0.05, **p < 0.01, and ***p < 0.001.

Plasma B.1 (D614G) S-specific IgG responses peaked at week 6 and then declined, as reported previously (*28*), but then stabilized throughout the one year follow-up period in the Protein group (Fig. 2A). In the mRNA group, plasma D614G S-specific IgG responses also persisted, but had declined by 23.8% from week 22 to week 52 (Fig. 2A). At week 52, the time of challenge, B.1.617.2 S-specific plasma IgG responses were of similar magnitude as those to the D614G S protein in each group, but with overall higher titers in the Protein vaccine group (Fig. 2A). Vaccine-induced IgG responses against the receptor-binding domain (RBD) of the B.1.617.2 S protein were also detectable in nasal, salivary, and rectal secretions (Fig. 2B). B.1.617.2-specific IgA activity was found in nasal secretions of 7 of 8 RMs and in saliva of 2 of 8 RMs vaccinated with the Protein vaccine, whereas only 1 RM of the mRNA group had B.1.617.2-specific IgA activity and only in saliva (Fig. 2C). IgG and IgA responses were higher in RMs immunized with the Protein vaccine compared to mRNA vaccinated RMs. The IgG and IgA antibodies in secretions of these animals were most likely derived from plasma transudate because parenteral immunization with mRNA vaccines or 3 M-052-adjuvanted protein vaccines does not typically induce local mucosal antibody responses in humans or NHPs (*37*–*39*).

**Fig. 3.**
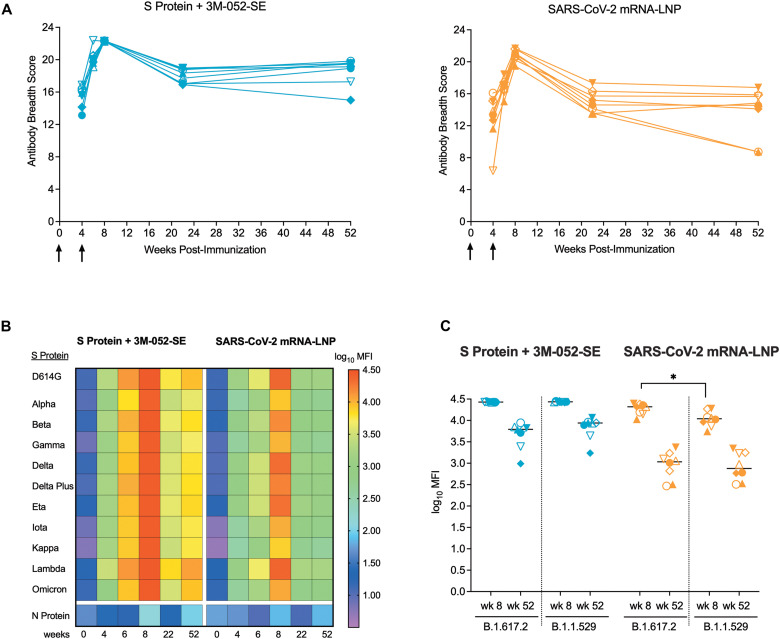
SARS-CoV-2 vaccination resulted in increased breadth of the plasma antibody response. (**A**)Each line represents the longitudinal measurements of the plasma IgG breadth scores of individual RMs in the Protein+3 M-052-SE (left graph) or mRNA-LNP (right graph) group. Arrows indicate time of vaccination. (**B**) The heatmap shows the median antibody binding as the log_10_ median fluorescence intensity (MFI) to 11 different S proteins over time in RMs vaccinated with the Protein or mRNA vaccine. Each row represents the antibody binding log_10_ MFI against one specific S protein (see legend for color code) and each column represents a specific week post-immunization. Antibody binding to the SARS-CoV-2 nucleoprotein (N protein) was negative at all time points. (**C**) Shown is a comparison of plasma antibody binding to the S proteins of the B.1.617.2 (Delta) and B.1.1.529 (Omicron) variants at peak response (wk 8) and at the time of challenge (wk 52) in RMs of the Protein and mRNA vaccine group. Horizontal lines represent group medians, each symbol represents an individual animal (table S1) with n = 8 RMs per group. Differences in antibody binding to the two distinct S proteins within each group at the same time point were determined by Mann-Whitney test with *p < 0.05.

At the time when the current challenge studies were performed, the B.1.617.2 variant was the dominant circulating variant. Since then, Omicron variants have emerged as the main variants. Therefore, we examined the breadth of the antibody response. Both vaccines had elicited cross-binding plasma IgG responses to 11 different SARS-CoV-2 variant S proteins (Fig. 3) that peaked at week 8 (Fig. 3A). Although binding strength differed dependent on the VOC, cross-reactive IgG binding responses to all tested S proteins, including Delta (B.1.617.2) and Omicron (B.1.1.529), were maintained in RMs of both vaccine groups to week 52 (Fig. 3B and C).

**Fig. 4.**
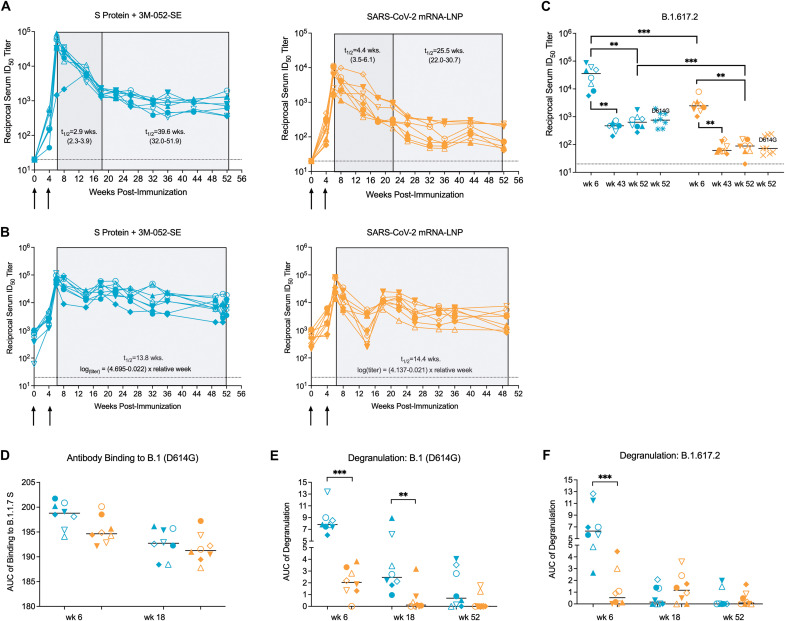
Vaccine-elicited plasma antibodies exhibit distinct degrees of effector functions. (**A** and **B**)The ID_50_ titers of D614G-specific neutralizing antibodies from week 0 to week 52 for individual RMs in the Protein+3 M-052-SE (left graph) or mRNA-LNP (right graph) group were measured by the pseudovirus assay (A) or the whole virus assay (B), respectively. Arrows indicate the time of immunizations. The shaded areas in (A and B) indicate the decay of nAbs. In the pseudovirus assay (A), the decay was characterized by an early rapid (dark gray) decay phase followed by a slower decline (light gray). The estimated half-life of nAbs in each phase is listed with the 95% confidence interval. The decay of nAbs measured in the whole virion assay (B) was calculated using the listed formula. (**C**) ID_50_ titers of B.1.617.2-specific nAbs are shown at week 6 (peak response), week 43, and week 52 for individual RMs in the Protein+3 M-052-SE (blue symbols) or mRNA-LNP (orange symbols) group in comparison to corresponding ID_50_ titers of D614G (B.1)-specific nAbs at week 52. (**D**) The ability of plasma antibodies to bind to D614G S protein-transfected cells is shown. Data are reported as AUC measured by S protein-expressing cell antibody binding assay. Dotted horizontal lines (A to C) indicate the lower limit of detection. (**E** and **F**)Shown is the degranulation activity of D614G-specific or B.1.617.2-specific antibodies, reported as AUC, respectively. Horizontal bars indicate medians, each symbol represents an individual RM (table S1); each group has n = 8 RMs. Differences between groups were determined by Mann-Whitney test and between two timepoints within a group by Wilcoxon rank test with *p < 0.05, **p < 0.01, and ***p < 0.001.

To determine the potential of these antibodies to protect against challenge with B.1.617.2, we assessed neutralizing activity and Fc-mediated effector functions using plasma. As reported previously, peak neutralizing antibodies (nAb) to the D614G virus were observed shortly after the second immunization (*28*). In the Protein group, D614G-specific half maximal inhibitory dilution (ID_50_) nAb titers declined 59-fold from a median of 44,794 (range: 1,452 to 89,867) at week 6 to a median of 755 (range: 359 to 1,949) at week 52 in a pseudovirus neutralization assay (Fig. 4A). D614G-specific ID_50_ nAb titers in the mRNA group were reduced 89-fold from a median of 6,430 at week 6 (range: 1,496 to 11,325) to 73 (range: 41 to 240) at week 52 (Fig. 4A). The kinetics of nAb decline were biphasic with an early steeper, followed by a slower decline over time. Applying a piecewise linear model, the half-life of nAb titers in the Protein group was estimated to be 2.9 weeks (95% CI 2.3 to 3.9 weeks) during weeks 6 to 18, and 39.6 weeks (95% CI 32.0 to 52.9 weeks) from week 18 to 52 (Fig. 4A). The half-life of the nAb response in the mRNA group was estimated to be 4.4 weeks (95% CI 3.5 to 6.1 weeks) and 25.5 weeks (95% CI 22.0 to 30.7 weeks) in the first and second phase, respectively (Fig. 4A). Results for whole virus assay nAb responses followed a similar pattern, although the decline in nAbs appeared to be more continuous and gradual (Fig. 4B). The half-life was estimated to be 13.8 weeks in the Protein group and 14.4 weeks in the mRNA group (Fig. 4B). The vaccines also induced cross-neutralizing antibodies to B.1.617.2. Despite 1.3-fold or 2.6-fold lower median ID_50_ titers against the B.1.617.2 variant versus the D614G variant at week 6 in the Protein or mRNA groups, respectively (Fig. 4C), median ID_50_ titers to the D614G and the B.1.617.2 variants were of comparable magnitude within each group at the time of challenge.

Both vaccines induced plasma antibodies able to bind to the D614G S protein on transfected cells (Fig. 4D). More importantly, at week 6 (the peak of antibody responses), binding of vaccine-induced antibodies to D614G S-transfected 293 T cells triggered degranulation, indicative of antibody-mediated cytotoxicity (ADCC) function. Despite comparable antibody binding, antibody-mediated degranulation at weeks 6, 18, and 52 was higher in RMs who had received the Protein compared to the mRNA vaccine (Fig. 4E). At week 6, antibody-mediated degranulation was also detectable against B.1.617.2 S-transfected cells (Fig. 4F). However, the responses against both variants decreased over time, with only few RMs maintaining degranulation activity at week 52 (Fig. 4E and F).

**Fig. 5.**
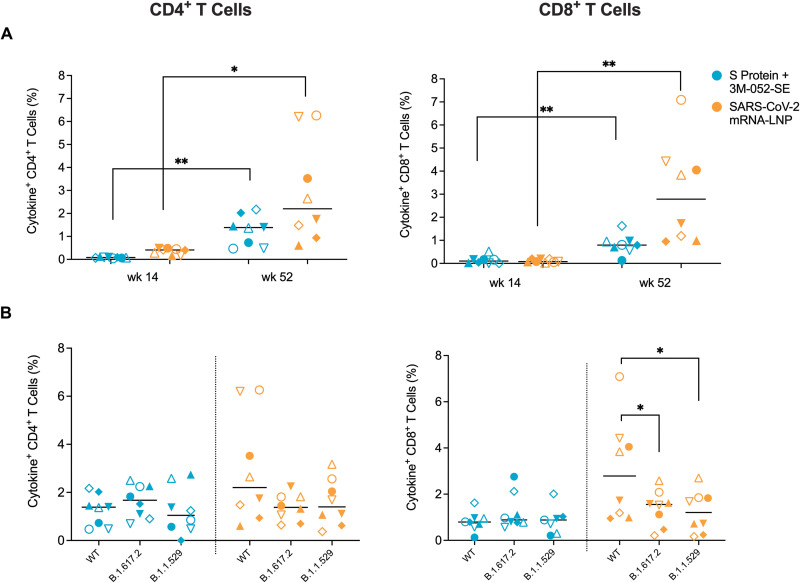
Vaccination elicits peripheral blood T cell responses in infant RMs. Peripheral blood CD4^+^ and CD8^+^ T cell responses were assessed after in vitro stimulation of PBMCs with a peptide pool spanning the wildtype, B.1.617.2 (Delta), or B.1.1.529 (Omicron) S protein and measuring the percentage of interleukin (IL)-2, interferon (IFN)-γ, IL-17, and tumor necrosis factor (TNF)-α producing CD4^+^ and CD8^+^ T cells by flow cytometry. The data represent the sum of all single, double, or triple cytokine positive CD4^+^ or CD8^+^ T cells as determined by Boolean gating. (**A**) CD4^+^ or CD8^+^ T cell responses to the WT protein at week 52 were compared to the previously published data of week 14 to illustrate the increase in T cell responses over time in both vaccine groups. (**B**) The magnitude of CD4^+^ or CD8^+^ T cell responses against the Delta or Omicron S proteins are shown in comparison to the WT at the time of challenge (week 52). Horizontal bars indicate medians, each symbol represents an individual RM as outlined in table S1 with n = 8 RMs per group. Differences between RMs of the same group at different timepoints were determined by Wilcoxon rank test with *p < 0.05 and **p < 0.01.

In addition to vaccine-induced antibody responses, we measured peripheral blood T cell responses to the ancestral S protein of the vaccine (wildtype or WT) and the B.1.617.2 variant at the time of challenge. In contrast to the decline in antibody responses, SARS-CoV-2 specific T cell responses were maintained and were higher at week 52 compared to week 14 (Fig. 5A). Median CD4^+^ T cell responses to the WT S protein at week 52 did not differ among the Protein (1.4%, range 0.5% to 2.4%) or mRNA (2.2%, range 0.9% to 6.3%) vaccine groups (Fig. 5A), whereas median CD8^+^ T cell responses to WT S protein were higher (p = 0.002, Mann Whitney) in the mRNA group (2.8%, range 0.9% to 7.1%) compared to RMs in the Protein group (0.8%, range 0.1% to 1.6%) (Fig. 5A). In both vaccine groups, cross-reactive median CD4^+^ T cell responses against S proteins of the B.1.617.2 and B.1.1.529 variants were of similar magnitude as those against the WT S protein (Fig. 5B). Median CD8^+^ T cell responses against the S proteins of the B.1.617.2 and B.1.1.529 variants were lower compared to WT S protein responses in the mRNA group, but B.1.617.2 and B.1.1.529 S-specific CD8^+^ T cell responses were of comparable magnitude and did not differ between the vaccine groups (Fig. 5B). There were no differences in the cytokine profile between RMs in the two different vaccine groups (table S2). Together, these results corroborated that the low-dose, two-dose regimens of both vaccines were effective in inducing cross-variant cellular and humoral SARS-CoV-2-specific immune responses that persisted for at least one year.

### Clinical symptoms, hematology, blood chemistry and plasma inflammatory markers varied after heterologous B.1.617.2 challenge in vaccinated RMs

From the day of challenge onwards, RMs were scored daily for several clinical signs, such as coughing, nasal discharge, or respiratory distress, by cage-side observations and scored for clinical parameters at each sedation (Fig. 1). No obvious differences between the groups were observed as even in the control group overall clinical signs were absent to mild (fig. S1) and RMs in all groups maintained stable weights. Consistent with clinical disease symptoms, despite some minor fluctuations in complete blood count (CBC) values (fig. S2), likely attributable to experimental procedures (such as blood collections and frequent sedations), CBC data were generally maintained within the normal range for this age group. Early declines in lymphocyte counts after B.1.617.2 challenge were consistent with observations in humans infected with SARS-CoV-2 (*40*). There were also no or only slight fluctuations in blood chemistry values, including C-reactive protein (fig. S3; table S3).

Systemically, SARS-CoV-2 infection was reflected by increased plasma concentrations of inflammatory cytokines (fig. S4 and table S4). At day 2 post-challenge, control RMs had elevated concentrations of interleukin (IL)-6, IL-1RA, eotaxin, monocyte chemoattractant protein-1 (MCP-1), CXCL10, and CXL11. In contrast, such changes were not observed in the Protein group, whereas the mRNA vaccinated RMs had intermediate patterns of increased inflammatory cytokine concentrations (fig. S4).

### Prior SARS-CoV-2 vaccination controlled virus replication in the upper and lower respiratory tract of infant RMs

**Fig. 6.**
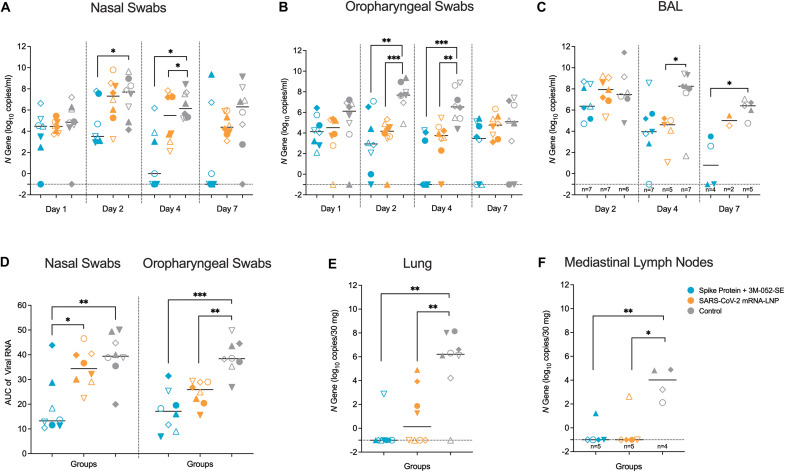
SARS-CoV-2 RNA concentrations are lower in mucosal secretions and tissues isolated from vaccinated RMs. (**A** to **C**) Virus replication was measured by qRT-PCR for the SARS-CoV-2 *N* gene. The viral RNA concentrations in nasal (A) and oropharyngeal (B) swabs were determined on days 1, 2, 4, and 7, and the viral RNA concentrations in BAL (C) on days 2, 4, and 7. Data are reported as log_10_ copies per ml for fluids and as copies per 30 mg for tissue samples. (**D**) Shown is area-under-the curve analysis of log_10_ transformed viral RNA data in nasal and oropharyngeal swabs throughout the 7-day challenge period. (**E** and **F**)Viral RNA concentrations in the lung (E) and mediastinal lymph nodes (F) were measured on day 7. Dashed lines indicated the lower limit of detection. Individual RMs in the different groups are represented by distinct symbols as outlined in table S1. (A, B, C, D and E) show data for n = 8 RMs per group; (C and E) show the number of RMs for which sufficient sample and RNA was available above the x-axis. Differences between the groups were determined by Mann-Whitney test with *p < 0.05, **p < 0.01, and ***p < 0.001.

Virus replication was monitored in the upper and lower respiratory tract by testing nasal and oropharyngeal swabs (days 1, 2, 4, and 7 post-challenge), bronchoalveolar fluid (BAL; days 2, 4, and 7), lung tissue, and mediastinal lymph node tissues (day 7) (Fig. 1) for SARS-CoV-2 N gene RNA expression to capture both genomic and subgenomic viral RNA; data for *orf1a,b* genomic RNA are presented in fig. S5. On day 1 post-challenge, high viral RNA (vRNA) abundance was observed in nasal or oropharyngeal swabs of all RMs independent of their vaccination status, and a similar pattern was observed in BAL on day 2, possibly reflecting residual challenge input virus (Fig. 6A to C). By days 2 and 4 post-challenge, median vRNA copies per ml in nasal and oropharyngeal secretions increased in control RMs, indicative of active virus replication (Fig. 6A and B); vRNA declined by day 7 (but with high individual variability) (Fig. 6A to C). In contrast, RMs in the Protein vaccine group demonstrated a decline in vRNA concentrations after day 1 and vRNA concentrations on days 2 and 4 were significantly lower (p < 0.05 and p < 0.01, respectively) than those of the control group. On day 7, vRNA was no longer detectable in 5 of 8 nasal and 3 of 8 oropharyngeal samples of the Protein group. Virus replication in the mRNA vaccine group was intermediate to the Protein vaccine and control group (Fig. 6). Differences in virus control dependent on the sampling site across all groups became more apparent when we compared vRNA area-under-the-curve (AUC) values over the total 7-day challenge period (Fig. 6D).

Similarly, the immunized RMs had lower median vRNA in BAL samples on day 4 compared to controls (8.3 log_10_ copies/ml versus 4.0 or 4.6 log_10_ copies/ml in the Protein or mRNA group, respectively) (Fig. 6C). Day 7 vRNA concentrations were also lower in BAL samples of the Protein compared to the control group (Fig. 6C). Finally, vRNA concentrations in lung and mediastinal lymph node tissues collected on day 7 revealed marked differences between the vaccinated groups and the control group (Fig. 6E and F). Correlations between virus replication in the upper and lower respiratory tract are presented in fig. S6 (see also table S5). In summary, although the vaccine regimens did not provide sterilizing immunity after high-dose challenge, the immunized RMs displayed faster clearance of vRNA compared to the control group, with the Protein vaccine group mediating the best control of virus replication. Overall, the vaccine-mediated reduction in virus replication was more pronounced in the lower than the upper respiratory tract.

### Reduced lung inflammation was observed in immunized animals after heterologous SARS-CoV-2 challenge

**Fig. 7.**
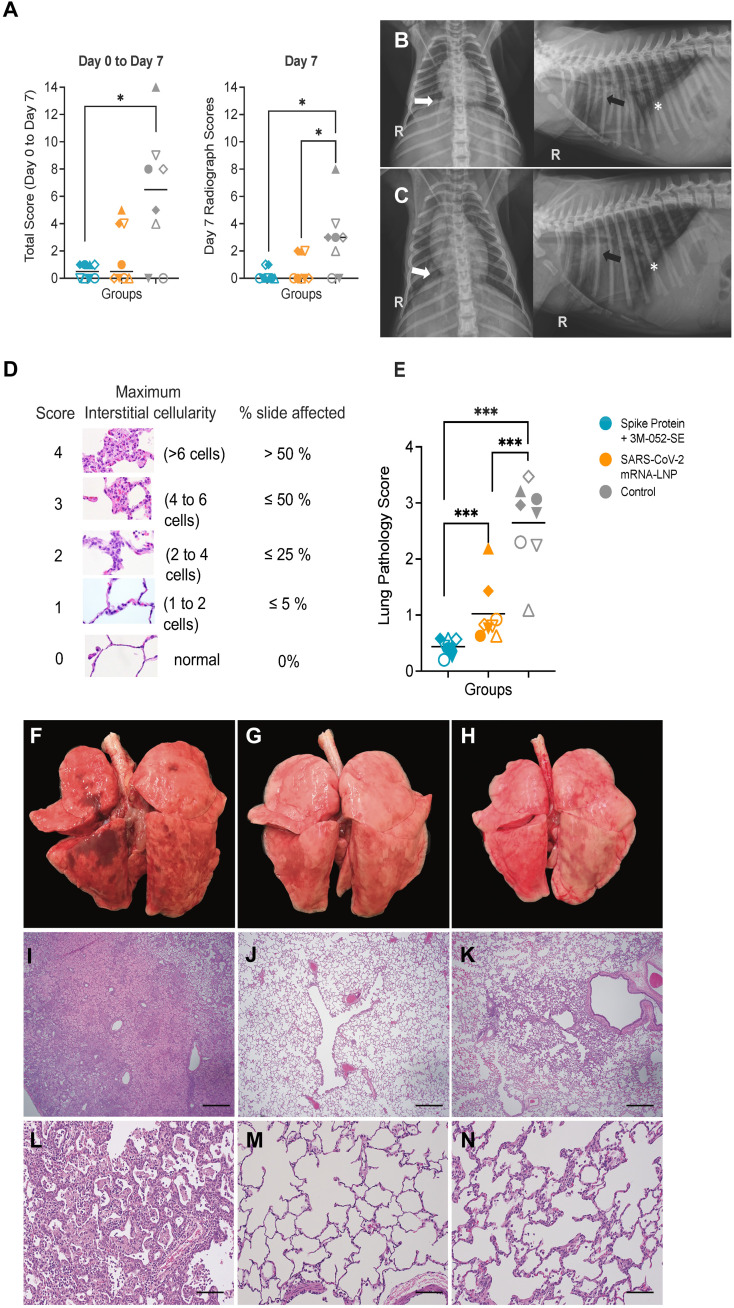
Lung pathology is reduced in vaccinated RMs compared to controls. (**A**) Lung radiograph scores are presented as the sum of daily scores from day 0 to day 7 (left graph) or on day 7 only (right graph). Differences of lung radiograph scores between two groups were determined by Mann-Whitney test with *p < 0.05. (**B** and **C**) Shown are the ventrodorsal and right lateral radiographs of the control animal RM 19, with the highest radiograph score, on the day of challenge (B) and at day 7 (C). On the baseline images, all lung lobes received a score of 0 meaning no evidence of pulmonary infiltrate was observed. On the day 7 images, scores are as follows: right cranial lung lobe 2, right middle lung lobe 1, right caudal lung lobe 2, accessory lung lobe 1, cranial segment of left cranial lung lobe 1, caudal segment of left cranial lung lobe 1, left caudal lung lobe 0. Note the reduced visibility of the pulmonary vasculature to the cranial segment of the left cranial lung lobe (black arrow), right caudal lung lobe (white arrow) and caudal vena cava (*) as a result of pulmonary infiltrates on day 7 in comparison to the baseline images. (**D**) An overview of the lung pathology scoring system is shown (table S7). (**E**) The average whole lung pathology scores are listed for each RM (n = 8 per group). Medians are indicated by horizontal bars. Differences of average lung pathology scores between two groups were determined by Mann-Whitney test with ***p < 0.001. (**F** to **H**) Shown are representative lung images for control RMs (F), and RMs vaccinated with the Protein+3 M-052-SE (G) or mRNA-LNP (H) vaccine; images of all lungs can be found in figure S7. (**I** to **N**) Shown are representative lung H&E images at 4x magnification (I to K) or 20x magnification (L to N) indicating the various degree of interstitial pneumonia in control RMs (I, L) Protein+3 M-053-SE (J, M), or mRNA-LNP (K, N) vaccinated RMs. The scale bar in (I to K) corresponds to 500 μm, and the scale bar in (L to N) to 100 μm.

Lung disease was assessed by radiographs collected prior to and on days 2, 4, and 7 post challenge. The different areas of the lung were scored for the radiologic presence of pulmonary infiltrates and an overall lung score was tabulated for each day (table S6). The median sum of individual scores from day 0 to day 7 and median day 7 only scores were highest in control RMs (Fig. 7A to C; p < 0.05 by Mann-Whitney two group comparisons), with no difference between RMs in the Protein or mRNA vaccine groups. By day 7, 6 of 8 and 5 of 8 RMs in the Protein or mRNA vaccine group, respectively, showed no evidence of pulmonary infiltrates compared to only 2 of 8 control RMs (Fig. 7A to C).

To gain further insight into lung pathology, we performed gross examination and selected four lobes – both caudal lobes, the right cranial lobe, and the left middle lobe – for histology examination on day 7 post challenge. Per animal, between 20 to 24 slides were blindly evaluated and scored based on the extent and severity of interstitial and alveolar inflammation as outlined in table S7. The comprehensive scoring system had been validated in prior studies as a very sensitive tool to detect efficacy of prophylactic and therapeutic interventions (*30*, *31*). Unvaccinated, SARS-CoV-2-infected control RMs could be easily distinguished from vaccinated RMs based on gross pathology, average individual lung lobe scores, or the average combined (4-lobe) whole lung pathology scores (Fig. 7D to N, fig. S7, table S8).

Most of the control RMs had moderate to severe interstitial pneumonia that was extensive in some of the lobes (Fig. 7D to N). In the most severe lesions, regions of alveolar inflammation were characterized by marked expansion of the alveolar septae by inflammatory cells, mostly macrophages and neutrophils, accompanied by variable numbers of macrophages and neutrophils in the alveolar lumen, consistent with pathology in adult RMs (*25*, *30*, *31*). Type 2 hyperplasia was relatively common, indicative of previous type 1 alveolar cell injury and loss. In some areas there was marked alveolar edema with scattered fibrin accumulation and occasional syncytial cells (Fig. 7D to N). In contrast, the lesions in the mRNA and Protein vaccinated RMs were generally non-existent to mild and localized, consisting of small foci of increased alveolar interstitial cellularity, and only occasionally with a very small increase in the numbers of alveolar macrophages (Fig. 7D to N). Two of the RMs immunized with the mRNA vaccine had lesions that were slightly more severe and extensive, and, although lung pathology did not approach the degree of severity or extent of those seen in the control RMs, the lung pathology scores in these 2 RMs overlapped with the lowest pathology scores observed in the control group (Fig. 7D to N; fig. S7). Both vaccinated groups had significantly lower lung pathology scores than the control group, and the scores of the Protein vaccine group were also lower than that of the mRNA group (all p values <0.001). The day 7 lung pathology scores were strongly correlated (Spearman rank test: r = 0.643; p = 0.0009) with lung radiograph scores on day 7 (table S5). Finally, median lung pathology scores and median lung viral RNA concentrations did not differ between male and female RMs (fig. S8).

To avoid any bias in our challenge outcome conclusions, we performed a sensitivity analysis excluding the 3 control RMs (#17, #18, and #21) that were housed outdoors until one month prior to SARS-CoV-2 challenge to determine if a potentially different microbiota in these 3 control RMs impacted virus replication or lung pathology. With the exception of lung virus replication in mRNA vaccinated RMs, differences in lung SARS-CoV-2 replication, lung pathology scores, and day 7 radiograph scores between control RM (n = 5) and vaccinated RMs remained statistically significant (p < 0.05, table S5, fig. S9). We therefore used data from all 8 control RMs in further analyses. In a prior study, the comprehensive pathology evaluation proved to be more sensitive in detecting lung pathology than radiographs (*41*), and, therefore, we selected the average combined lung pathology score, referred to as lung pathology score, in subsequent analyses of challenge outcome and in the identification of immune correlates of protection.

### Immunized infant RMs mounted anamnestic immune responses to SARS-CoV-2 challenge

Generally, despite a few exceptions, antibody responses reflected anamnestic responses in the vaccine groups and primary responses in the control group. On day 7, compared to day 0, RMs in both vaccine groups had increased plasma IgG concentrations specific to the D614G and the B.1.617.2 S proteins (fig. S10A and B) and increased B.1.617.2 RBD-specific IgG responses in nasal secretions (fig. S10C). Control RMs mounted S-specific IgG responses at day 7 post-challenge in plasma, but not in mucosal secretions (fig. S10). Modest but significant (p = 0.0391) increases in B.1.617.2 RBD-specific IgA were also detected in nasal secretions of the Protein group (fig. S10E). Like IgG, these IgA antibodies represented anamnestic responses because no IgA or IgG antibodies were apparent in secretions of naive controls on day 7 post-infection (fig. S10). IgG bound to S proteins of 11 VOCs at day 0 and day 7 with slight variation in magnitude between VOCs, and with the mRNA vaccine group exhibiting increased binding on day 7 compared to day 0 (fig. S11). In the Protein group, post-challenge plasma antibody responses resulted in higher Fc-mediated degranulation activity against the D614G, but not the B.1.617.2 S protein, indicative of an anamnestic vaccine response (fig. S12A and B). nAb responses of Protein vaccinated RMs did not change in response to viral challenge (fig. S12C and D). In contrast, RMs in the mRNA group responded with increased plasma neutralizing activity against the vaccine virus, but no change in Fc-mediated antibody function (fig. S12C and D). Control RMs developed low concentrations of plasma neutralizing B.1.617.2-specific IgG responses at day 7 post-challenge (fig. S12C and D).

To distinguish between anamnestic and challenge virus-induced T cell responses, we assessed day 7 peripheral blood T cell responses to the S proteins of the WT virus and the B.1.617.2 variant and to the nucleocapsid (N) protein of the WT strain that was not expressed by the vaccine (fig. S13). Vaccinated RMs, regardless of the vaccine type, did not exhibit increased peripheral blood CD4^+^ or CD8^+^ T cell responses to the WT or the B.1.617.2 S protein on day 7 compared to day 0 post-challenge. RMs of the Protein vaccine group, however, mounted CD8^+^ T cell responses to the N protein. N-specific T cell responses were also observed in the CD4^+^, but not the CD8^+^ T cell population of control RMs, consistent with observations in unvaccinated adult RMs (*42*). Further, RMs in the control group had evidence of peripheral blood N-specific CD4^+^ T cell responses. The majority of vaccinated and control RMs also had detectable CD4^+^ and CD8^+^ T cell responses in mediastinal lymph nodes.

### Neutralizing antibody responses against wildtype and B.1.617.2 SARS-CoV-2 at one year post vaccination correlated with protection against SARS-CoV-2 challenge

As lung pathology scores proved to be the most relevant marker to evaluate relative vaccine efficacy in this animal model of COVID-19 and were reflective of virus replication (fig. S14), we tested what vaccine-induced immune responses at the time of challenge correlated best with protection against lung inflammation. All virus-specific antibody parameters on the day of challenge, with the exception of Fc-mediated degranulation function of B.1.617.2-specific plasma antibodies, were inversely correlated with lung inflammation (r values range: −0.66 to −0.92; all p values ≤0.007) (table S5). These antibody responses included (i) serum neutralizing antibodies (assessed by both pseudovirus and whole virus assay) against both D614G and B.1.617.2, (ii) D614G or B.1.617.2 S-specific binding IgG in plasma, (iii) B.1.617.2 RBD-specific IgG in nasal or salivary secretions, (iv) B.1.617.2 RBD-specific IgA in nasal secretions, (v) plasma antibody binding to other VOCs, and (vi) Fc-mediated degranulation function of D614G-specific antibodies (fig. S15, table S5). Considering these strong inverse correlations, we tested whether peak antibody responses at week 6 could already predict challenge outcome and found indeed strong inverse correlations between plasma IgG binding and neutralizing antibodies, and antibody Fc-mediated degranulation against both the D614G and the B.1.617.2 S proteins and lung pathology scores (fig. S16).

Day of challenge peripheral blood T cell responses also appeared to contribute to vaccine-mediated protection. B.1.617.2 S protein-specific CD4^+^, but not CD8^+^, T cell responses were inversely correlated with lung pathology scores (r = −0.524, p = 0.0095). In contrast, positive correlations were observed between mediastinal lymph node N-specific CD4^+^T cell responses and day 4 oropharyngeal vRNA concentrations (0.509, p = 0.0116), and between lung pathology and mediastinal lymph node N-specific CD4^+^ T cell responses (r = 0.522, p = 0.0096) and B.1.617.2 S-specific CD8^+^ T cell responses (r = 0.451, p = 0.0287) (table S5), implying that these T cell responses were likely induced in response to virus replication.

**Fig. 8.**
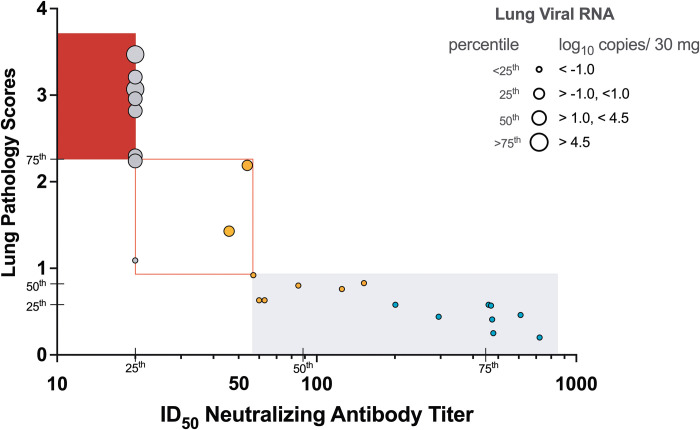
Neutralizing ID_50_ antibody titers are a correlate of protection in infant RMs challenged with SARS-CoV-2. ID_50_ titers of B.1.617.2-specific nAbs at the time of challenge are plotted against lung pathology scores. The symbol size for each RM is based on the degree of virus replication in the lung of the same RM. RMs in the Protein or mRNA vaccine groups are indicated by blue and orange circles, respectively, and control RMs by gray circles. Individual animal symbols were not used to more clearly document the impact of vaccine-induced nAb ID_50_ titers on lung inflammation and virus replication. Note that RMs with ID_50_ titers above 50 have pathology scores below 1. Vertical ticks indicate the 25th, 50th and 75th percentile of all ID_50_ titers across all groups and horizontal ticks indicate the 25th, 50th and 75th percentile of lung pathology scores (table S9). Most control RMs have ID_50_ nAb titers at or below the 25th percentile and pathology scores above the 75th percentile, whereas the majority of Protein+3 M-052 vaccinated RMs have ID_50_ nAb titers above the 75th percentile and pathology scores at or below the 25th percentile. RMs of the mRNA-LNP group have intermediate (between the 25th and 75th percentile) ID_50_ nAb titers and pathology scores. Please see figure S17 for the data with D614G nAb. Red-filled, red-outlined, and light gray shaded areas indicate severe, intermediate and no or mild lung pathology, respectively.

To obtain quantitative measurements that could be applied to identify immune correlates of vaccine-induced protection, immune response parameters on the day of challenge (day 0) and parameters of challenge outcome, including lung pathology scores, SARS-CoV-2 RNA concentrations, clinical and cage observation scores, and day 2 increases in plasma cytokines were tabulated across the three groups (see Materials and Methods). The 25th, 50th, and 75th percentiles for each parameter were calculated (table S9), followed by assignment of scores from 1 to 4 corresponding to the respective quartile from lowest to highest. The data were ordered from lowest (no or mild) to highest (severe) challenge outcome score (fig. S17), with separate matrices for WT/D614G or B.1.617.2 S-specific immune responses. The overall conclusions from both analyses were similar: (i) the higher immune response scores on the day of challenge, the less severe was challenge outcome, and (ii) neutralizing antibody responses were the main driver of protection against lung pathology and SARS-CoV-2 replication (Fig. 8). Thus, RMs with SARS-CoV-2 S-specific nAb responses ≥75th percentile (pseudovirus assay: D614G ID_50_≥430 or B1.617.2 ID_50_≥448) presented with normal lung histology and did not show evidence of virus replication in the lung (Fig. 8). In RMs with nAb titers within the range of the 50th to 75th percentile (RM12, RM14) and WT or B.1.617.2 S-specific CD4^+^ T or CD8^+^ T cell responses ≥75th percentile appeared to have partial protection (fig. S18). The results further emphasized that the magnitude of the responses elicited by the Protein vaccine provided superior efficacy compared to the mRNA vaccine (Fig. 8, fig. S18).

## DISCUSSION

SARS-CoV-2 vaccination has provided substantial protection against severe disease and hospitalization in all age groups for which the vaccine is available. The recognition of the potential benefits of SARS-CoV-2 vaccination of children was recently met by the U.S. Food and Drug Administration’s Emergency Use Authorization (EUA) of mRNA-based SARS-CoV-2 vaccines for infants and toddlers from 6 months to 4 or 5 years of age (*43*). Although this EUA is based on clearly favorable efficacy and safety data, many parents are likely to remain skeptical and wait to get their infants and toddlers immediately immunized due to concerns on safety and durability of efficacy, including against VOCs. Results of the current study provide a valuable contribution to efforts to alleviate some of these concerns.

Our study addressed the question whether SARS-CoV-2 vaccines administered in early life at doses much lower than those given to adults can protect against challenge with a heterologous SARS-CoV-2 variant one year after immunization. The results demonstrate that both the WT S-2P Protein+3 M-052-SE vaccine and the preclinical version of the SARS-CoV-2 mRNA-LNP vaccine given to 2-month-old RMs induce cellular and humoral immune responses of comparable magnitude, quality and durability as those elicited by similar vaccines in adult RMs (*25*) and these responses can protect against lung pathology, as assessed by radiograph images and histology, after high dose challenge with the B.1.617.2 variant one year after immunization.

Human and NHP studies of adults have documented the decline of nAb after SARS-CoV-2 vaccination or infection (*18*, *25*, *44*–*46*). Our knowledge about the durability of SARS-CoV-2 immune responses in infants and children, however, is still limited (*47*, *48*). The decay estimates of nAbs from week 6 to week 48 for both the Protein and the mRNA vaccine are within the range of those obtained in modeling studies of SARS-CoV-2 infection- or vaccine-induced nAbs with predicted half-lives varying from as low as 20 days to more than 200 days (*19*, *20*, *44*, *49*). Analogously, in adult RMs vaccinated with the mRNA-1273 vaccine, geometric mean titers (GMT) of nAb against the D614G variant (whole virus assay) had declined by almost 90% from week 6 to week 48 (*25*), a decline also comparable to the one (89%) observed in the current study for the mRNA vaccinated RMs. Similarly, the reduction of nAb titers to the B.1.617.2 variant compared to those against the B.1 (D614G) variant at peak response (week 6) is well-aligned with differences in peak nAb titers found in other human and NHP studies (*19*, *25*). Our findings are also consistent with the recent immunogenicity and efficacy data of the Pfizer and Moderna mRNA vaccines for human infants between 6 months and 5 years of age (*14*, *15*), and together these results demonstrate the robustness of the infant immune response to SARS-CoV-2 mRNA and protein vaccine platforms.

Importantly, compared to mRNA vaccine studies in adult RMs and humans, the two vaccines tested at lower doses in the current study provided comparable protective efficacy as adult mRNA vaccines against infection with heterologous SARS-CoV-2 variants (*19*, *25*). This result is even more remarkable considering that that the challenge dose in the study here was 10-fold higher compared to the study of adult RMs vaccinated with the adult mRNA-1273 vaccine dose and challenged with the B.1.617.2 variant one year later (*25*). Furthermore, and similar to our results, during the first days after challenge adult RMs also had high concentrations of viral RNA in upper respiratory secretions and BAL samples (*25*). Prevention of SARS-CoV-2 replication in the upper respiratory tract would likely require the induction of locally-produced mucosal antibodies by vaccination. The latter should be an important consideration in the development of next generation vaccines. However, RMs in both vaccine groups exhibited more rapid viral clearance that likely translates into an overall reduced transmission risk. Furthermore, the young immunized RMs were protected against the moderate to severe lung pathology observed in unvaccinated control RMs. Thus, even though these vaccines cannot prevent infection in a high-dose challenge model a year after immunization, they confer major benefits.

In addition to efficacy against lung disease, pediatric SARS-CoV-2 vaccines may offer much broader benefits. Long COVID is becoming a growing concern not only for adults but also for children (*50*). Prior studies in adult RMs have demonstrated that SARS-CoV-2 infection, most likely through the olfactory route, invades the brain and may cause neuropathology (*51*, *52*). Accordingly, currently ongoing studies are analyzing the brains of the RMs in this study to explore whether the reduced viral replication in the respiratory tract of immunized animals translates into protection against such neuropathology.

Protective efficacy in adult human clinical trials (*44*, *46*, *53*) and in adult NHP (*24*, *25*, *54*) vaccine studies has been associated with vaccine-elicited neutralizing antibody responses. Our results extend this conclusion to infants younger than 5 years. The plasma nAb titers to both the D614G or the B.1617.2 S protein at the time of challenge were inversely correlated with lung SARS-CoV-2 RNA concentrations and pathology. Our data suggest that pseudovirus median ID_50_ nAb titers of approximately 400 against the D614G or B.1.617.2 variants provided protection from severe lung inflammation. These titers are remarkably similar to the protective titers (ID_50_ > 500) reported by adoptive transfer of IgG from SARS-CoV-2 convalescent adult RMs to naïve RMs (*16*). In the same study ID_50_ nAb titers >50 provided partial protection (*16*), also similar to the findings in the current study as the two mRNA vaccinated RMs with ID_50_ nAb titers below 50 against the B.1.617.2 variant had mild to moderate lung inflammation. Our data are also consistent with mRNA-LNP vaccine studies in adult RMs that found that lower, suboptimal antiviral antibody concentrations may not be able to reduce virus replication in upper respiratory tract but can still confer protection against virus replication in the lower respiratory tract (*24*).

Passive immunization studies (such as with monoclonal antibodies) have shown clearly that sufficient amounts of neutralizing antibodies by themselves can induce protection against infection and pathology (*16*, *30*, *31*). Our data also suggest that Fc-mediated antibody functions and vaccine-induced T cell responses contribute to protection especially when nAb titers are suboptimal, as has been described earlier in studies with adult RMs (*16*). In the current study, this may be especially relevant for the mRNA vaccine group, as CD4^+^ T cell responses at the time of challenge were inversely correlated with lung pathology scores and provided extra predictability beyond that of nAb responses. Bearing in mind that vaccine-induced antibody responses declined over time, whereas T cell responses in blood were maintained during the year post-immunization, it would be interesting to determine the kinetics of SARS-CoV-2-specific T cell responses by different vaccines and to dissect the relative contribution of antibody versus T cell responses to vaccine-mediated protective efficacy over time after immunization. A protective role of T cell responses against SARS-CoV-2 infection is supported by early data from the B162b2 mRNA vaccine trial demonstrated that 90% of protection was already achieved by day 14, prior to the induction of neutralizing antibody responses in most vaccinees (*55*, *56*).

It remains to be determined how the results of the study will translate into protective efficacy against currently circulating Omicron and future variants. However, current mRNA vaccines are predicted to have potent cross-reactive T cell responses to the Omicron variant (*57*) and the mutations in the Omicron RBD regions are thought to reduce binding to the human angiotensin converting enzyme (ACE) 2 receptor and therefore, reduce lung pathogenicity (*58*). Importantly, in the BNT162b phase 2/III trial in children ages 6 months and older, the mRNA vaccine was reported to have 80% efficacy against the BA.1 variant, although a 3-dose regimen of a lower dose mRNA-LNP vaccine than that used in this study was applied (*15*).

The advantages of mRNA vaccines to protect against SARS-CoV-2 disease are multifold: they are highly immunogenic, safe, and easily adaptable to express antigens of emerging variants, and can be produced at high amounts. Yet, the finding that the Protein+3 M-052-SE vaccine induced higher and more durable immune responses that translated into better efficacy compared to the mRNA-LNP vaccine raises the question about the optimization of alternative SARS-CoV-2 vaccines, including potentially prime-boost regimens that mix different vaccines to improve durability and breadth of immune responses. We and others have previously demonstrated that TLR 7/8-based adjuvants, including 3 M-052, are superior to other adjuvants in inducing durable antibody responses to HIV and pneumococcal vaccines in infant macaques (*59*, *60*), and to SARS-CoV-2 in juvenile macaques (*61*). Furthermore, the Protein+3 M-052-SE vaccine might be more amenable for use in developing countries because it only requires refrigeration. Eliciting high and long-lived immune responses is especially relevant for SARS-CoV-2 vaccines given during early life, both to overcome the potential interference of maternal antibodies, and to protect against newly evolving variants that may be encountered later in life (*62*, *63*).

The current study was limited by relatively small group sizes of 8 RMs and the short follow-up period after SARS-CoV-2 challenge, factors largely driven by limited BSL-3 facility capacity. All RMs were dam-reared and breast-fed. However, compared to RMs in the vaccine groups and the majority of the control RMs (5 of 8), who were housed indoors, 3 of 8 control RMs (#17, #18, and #21) were transitioned to indoor housing only one month prior to challenge. To control for a potential impact of differences in host microbiota on immune responses to SARS-CoV-2 challenge, we performed a sensitivity correlation analysis that excluded these three RM from the control group. The main conclusions of our study were confirmed by this additional analysis (table S5, fig. S9). We also do not know how effective vaccine doses determined in infant rhesus macaques translate into human infant vaccine doses. Thus, although infant RM studies cannot replace human infant vaccine safety and dosing studies, the similarities in human and RM infant physiology and immune development support the use of the pediatric RM model to inform human vaccine trial design. This conclusion is strengthened by the concurrence of our major findings with observations in human studies.

In summary, the results of the current study demonstrate that despite lower vaccine doses, the infant Protein+3 M-052-SE and mRNA-LNP vaccines induced plasma antibody responses of similar magnitude, quality, breadth, and durability as those elicited by adult vaccines and that provided comparable protective efficacy against infection with a heterologous SARS-CoV-2 variant. Our findings complement and add to the data on durability and cross-reactivity of vaccine-induced antibody and T cell responses. This body of data will inform the timing of booster immunization and the optimization of existing or the design of improved SARS-CoV-2 vaccines, including pan-coronavirus vaccines. Altogether, the highly favorable safety, immunogenicity, and efficacy data of SARS-CoV-2 vaccines in the current RM study and matching data coming out of human pediatric trials provide strong support to initiate SARS-CoV-2 vaccination in infancy and incorporate new SARS-CoV-2 vaccines into the global routine pediatric vaccine schedules to curb the SARS-CoV-2 pandemic.

## MATERIALS AND METHODS

### Study design

Two groups of 2-month old RMs (n = 8 per group; table S1) were immunized IM at weeks 0 and 4 with stabilized prefusion SARS-CoV-2 S-2P spike (S) protein of the Washington strain encoded by mRNA encapsulated in lipid nanoparticles (mRNA group) or purified S protein mixed with 3 M-052, a synthetic TLR7/8 agonist, in a squalene emulsion (Protein group) (*28*). At approximately one year after the first immunization, the 2 vaccine groups and an added control group of 8 age-matched non-immunized RMs (table S1) were exposed to a high-dose heterologous challenge with the B.1.617.2 (Delta) variant, administered by the IT and IN routes. After challenge, animals were monitored for clinical signs of infection and virus replication and then euthanized for tissue collection on day 7 (Fig. 1). Vaccine efficacy was assessed in a blinded manner by challenge virus replication and lung pathology. Vaccine-induced immune responses were measured prior to and 7 days after challenge to determine correlates of protection.

### Animals

Infant male (n = 8) and female (n = 8) rhesus macaques (*Macaca mulatta*; RM) of Indian-origin from the California National Primate Research Center (CNPRC, Davis, CA) breeding colony (negative for type D retrovirus, simian immunodeficiency virus, simian lymphocyte tropic virus type 1 and SARS-CoV-2) were enrolled at a median age of 2.2 months and randomly assigned into two groups (table S1). Infants were housed indoors with their dams until weaning at 7 to 8 months of age and then pair-housed. At week 49, age- and sex-matched unvaccinated animals (n = 8) were added (table S1). Control RMs were also dam-reared and weaned between 5 to 8 months of age. Three of 8 control RMs were initially housed outdoors in the same corral and transitioned to indoor pair-housing approximately 1 month prior to challenge. Animal care followed the *Guide for Care and Use of Laboratory Animals* by the Institute for Laboratory Animal Research. Animal procedures were approved by the UC Davis Institutional Animal Care and Use Committee prior to study initiation. RMs were moved into the animal biosafety level 3 (ABSL-3) facility just before virus challenge inoculation.

### Vaccines

The SARS-CoV-2 stabilized prefusion S (S-2P) mRNA vaccine formulated in lipid nanoparticles (LNP) was provided by Moderna, Inc. For the adjuvanted protein vaccine, the Vaccine Research Center (National Institutes of Health, NIH) provided the S-2P Protein, whereas Access to Advanced Health Institute (AAHI) and 3 M worked together to provide the 3 M-052-SE adjuvant (*28*). To balance immunogenicity and safety, we decided to immunize the infant RMs in the mRNA-LNP group IM at weeks 0 (quadriceps) and 4 (biceps) with 30 μg of mRNA-LNP as described (*28*). The vaccine was stored at −20°C until just prior to the immunization. Infant RMs in the Protein+3 M-052-SE vaccine group were immunized IM with 15 μg S-2P protein mixed with 3 M-052-SE - an adjuvant consisting of 10 μg of the synthetic TLR7/8 agonist 3 M-052 in a 2% squalene-in-water emulsion (Protein+3 M-052-SE) - in 0.5 mL divided across the left and right quadriceps (week 0) or biceps (week 4).

### SARS-CoV-2 challenge

The challenge virus was obtained through the Biodefense and Emerging Infections Research Resources Repository (BEI Resources), National Institute of Allergy and Infectious Diseases (NIAID), NIH: SARS-Related Coronavirus 2, Isolate hCoV-19/USA/MD-HP05647/2021 (Lineage B.1.617.2; Delta variant) (WCCM), NR-55674, contributed by Andrew S. Pekosz: titer: 1.8x10^7^ Median Tissue Culture Infectious Dose (TCID_50_) per mL. The virus was stored at −80°C. A new vial was thawed immediately prior to each animal inoculation and diluted 9-fold in phosphate-buffered saline (PBS). At approximately 52 weeks after the first immunization animals were challenged with this virus IT (2x10^6^ pfu in 1 mL) and IN (1x10^6^ pfu, 0.25 mL per nostril) (Fig. 1).

### Clinical observations

Daily cage-side clinical monitoring was performed by trained staff who were blinded to the group assignments, and included recording of responsiveness, discharge, respiratory rate and character, evidence of coughing or sneezing, appetite, stool quality. A score was tabulated for each of these parameters, and a total score was calculated for each animal per day. When RMs had to be sedated for procedures, additional clinical assessments (including rectal temperature, respiration, oxygen saturation (SpO2), heart rate, and skin turgor/hydration) were recorded as described earlier (*30*). Animals were sedated with ketamine HCl (10 mg/kg IM) for the clinical assessment. Dexmedetomidine (15 mcg/kg IM) was administered after clinical assessments to facilitate sampling, and midazolam (0.25 to 0.5 mg/kg IM) was added as needed. SpO2 was obtained by pulse oximetry with a Radical 7 (Masimo, Irvine, CA).

### Lung radiograph examinations

Radiographs were obtained with a HF100+ Ultralight imaging unit (MinXRay, Northbrook, IL) at 50 kVp, 40 mA, and 0.1 sec. Ventrodorsal, dorsoventral, right (R) lateral, and left (L) lateral radiographs were obtained prior to and on days 2, 4, and 7 after challenge. Radiographs were scored for the presence of pulmonary infiltrates by a board-certified veterinary radiologist, who was blinded to the experimental group and time point, according to a standard scoring system (0: normal; 1: mild interstitial pulmonary infiltrates; 2: moderate pulmonary infiltrates perhaps with partial cardiac border effacement and small areas of pulmonary consolidation; 3: severe interstitial infiltrates, large areas of pulmonary consolidation, alveolar patterns and air bronchograms). Individual lobes were scored and total lung scores per animal per day and over the 4 time points were tabulated.

### Statistical Analyses

All raw, individual-level data are presented in data file S1. Between-group comparisons utilized the nonparametric Wilcoxon signed rank test, and within-group comparisons utilized the nonparametric Mann-Whitney U test. Associations were assessed using the Spearman rank correlation coefficient test. All reported p-values are exact. When computing exact p-values was not computationally feasible, a Monte-Carlo method was utilized to approximate the exact p-value. The Benjamini-Hochberg procedure was employed to control the false discovery rate (FDR) in the correlation analyses. FDR corrections were applied separately for the correlates of protection, correlates of pathogenicity, and inter-strain correlations analyses. P-values where p < 0.05 before the FDR correction. Longitudinal generalized estimating equations (GEE) models were fit on neutralizing antibody data post week 6 with a continuous time component as the predictor, log-transformed neutralizing antibody as the outcome, and a random intercept for each animal. Pseudovirus neutralization data showed a change in the log-linear trend over time, so piecewise linear models were fit, with the knot placement determined by selecting the model with the lowest quasi-likelihood under the independence model criterion (QIC). Decay rates were calculated by exponentiating the slope of the log-linear models, and antibody half-life was calculated accordingly. Analysis was performed in GraphPad Prism version v9.4.0 and SAS version 9.4.

## Supplementary Materials

### Other Supplementary Material for this manuscript includes the following:

MDAR Reproducibility Checklist

## Notes

View/request a protocol for this paper from *Bio-protocol*.

## References

[1] WHO. (WHO, 2022), vol. 2022.

[2] P. Zimmermann, N. Curtis, Why is COVID-19 less severe in children? A review of the proposed mechanisms underlying the age-related difference in severity of SARS-CoV-2 infections. Arch. Dis. Child. 106, 429–439 (2021). 10.1136/archdischild-2020-320338 33262177

[3] P. Zimmermann, N. Curtis, COVID-19 in Children, pregnancy and neonates: A review of epidemiologic and clinical features. Pediatr. Infect. Dis. J. 39, 469–477 (2020). 32398569 10.1097/INF.0000000000002700 PMC7363381

[4] A. A. Butt, H. Chemaitelly, A. Al Khal, P. V. Coyle, H. Saleh, A. H. Kaleeckal, A. N. Latif, R. Bertollini, A. B. Abou-Samra, L. J. Abu-Raddad, SARS-CoV-2 vaccine effectiveness in preventing confirmed infection in pregnant women. J. Clin. Invest. 131, e153662 (2021). 34618693 10.1172/JCI153662 PMC8631593

[5] W. J. Moss, L. O. Gostin, J. B. Nuzzo, Pediatric COVID-19 vaccines: What parents, practitioners, and policy makers need to know. JAMA 326, 2257–2258 (2021). 34739041 10.1001/jama.2021.20734

[6] CDC. (CDC, cdc.gov, 2022), vol. 2022.

[7] K. J. Marks, M. Whitaker, N. T. Agathis, O. Anglin, J. Milucky, K. Patel, H. Pham, P. D. Kirley, B. Kawasaki, J. Meek, E. J. Anderson, A. Weigel, S. Kim, R. Lynfield, S. L. Ropp, N. L. Spina, N. M. Bennett, E. Shiltz, M. Sutton, H. K. Talbot, A. Price, C. A. Taylor, F. P. Havers; COVID-NET Surveillance Team, Hospitalization of infants and children aged 0-4 years with laboratory-confirmed COVID-19 - COVID-NET, 14 States, March 2020-February 2022. MMWR Morb. Mortal. Wkly Rep. 71, 429–436 (2022). 35298458 10.15585/mmwr.mm7111e2 PMC8942304

[8] L. L. Chen, G. T. Chua, L. Lu, B. P. Chan, J. S. Wong, C. C. Chow, T. C. Yu, A. S. Leung, S. Y. Lam, T. W. Wong, H. W. Tsang, I. C. Wong, K. H. Chan, K. Y. Yuen, P. Ip, M. Y. Kwan, K. K.-W. To, Omicron variant susceptibility to neutralizing antibodies induced in children by natural SARS-CoV-2 infection or COVID-19 vaccine. Emerg. Microbes Infect. 11, 543–547 (2022). 35084295 10.1080/22221751.2022.2035195 PMC8843159

[9] L. Wang, N. A. Berger, D. C. Kaelber, P. B. Davis, N. D. Volkow, R. Xu, COVID infection severity in children under 5 years old before and after Omicron emergence in the US. *medRxiv*, 2022.01.12.22269179 (2022). 10.1101/2022.01.12.22269179.

[10] C. A. Pierce, S. Sy, B. Galen, D. Y. Goldstein, E. Orner, M. J. Keller, K. C. Herold, B. C. Herold, Natural mucosal barriers and COVID-19 in children. JCI Insight 6, e148694 (2021). 33822777 10.1172/jci.insight.148694 PMC8262299

[11] C. A. Pierce, P. Preston-Hurlburt, Y. Dai, C. B. Aschner, N. Cheshenko, B. Galen, S. J. Garforth, N. G. Herrera, R. K. Jangra, N. C. Morano, E. Orner, S. Sy, K. Chandran, J. Dziura, S. C. Almo, A. Ring, M. J. Keller, K. C. Herold, B. C. Herold, Immune responses to SARS-CoV-2 infection in hospitalized pediatric and adult patients. Sci. Transl. Med. 12, eabd5487 (2020). 32958614 10.1126/scitranslmed.abd5487 PMC7658796

[12] S. P. Weisberg, T. J. Connors, Y. Zhu, M. R. Baldwin, W. H. Lin, S. Wontakal, P. A. Szabo, S. B. Wells, P. Dogra, J. Gray, E. Idzikowski, D. Stelitano, F. T. Bovier, J. Davis-Porada, R. Matsumoto, M. M. L. Poon, M. Chait, C. Mathieu, B. Horvat, D. Decimo, K. E. Hudson, F. D. Zotti, Z. C. Bitan, F. La Carpia, S. A. Ferrara, E. Mace, J. Milner, A. Moscona, E. Hod, M. Porotto, D. L. Farber, Distinct antibody responses to SARS-CoV-2 in children and adults across the COVID-19 clinical spectrum. Nat. Immunol. 22, 25–31 (2021). 33154590 10.1038/s41590-020-00826-9 PMC8136619

[13] V. Singh, V. Obregon-Perko, S. A. Lapp, A. M. Horner, A. Brooks, L. Macoy, L. Hussaini, A. Lu, T. Gibson, G. Silvestri, A. Grifoni, D. Weiskopf, A. Sette, E. J. Anderson, C. A. Rostad, A. Chahroudi, Limited induction of SARS-CoV-2-specific T cell responses in children with multisystem inflammatory syndrome compared with COVID-19. JCI Insight 7, e155145 (2022). 35044955 10.1172/jci.insight.155145 PMC8876428

[14] Moderna, accesswire, Ed. (investors.modernatx.com, online, 2022), vol. 2022.

[15] Pfizer. (https://www.pfizer.com/news/press-release/press-release-detail/pfizer-biontech-covid-19-vaccine-demonstrates-strong-immune#:~:text=search%20results%20for-,Pfizer%2DBioNTech%20COVID%2D19%20Vaccine%20Demonstrates%20Strong%20Immune%20Response%2C,of%20Age%20Following%20Third%20Dose&text=NEW%20YORK%20%26%20MAINZ%2C%20Germany%2D%2D(BUSINESS%20WIRE)%2D%2D%20Pfizer%20Inc, 2022), vol. 2022.

[16] K. McMahan, J. Yu, N. B. Mercado, C. Loos, L. H. Tostanoski, A. Chandrashekar, J. Liu, L. Peter, C. Atyeo, A. Zhu, E. A. Bondzie, G. Dagotto, M. S. Gebre, C. Jacob-Dolan, Z. Li, F. Nampanya, S. Patel, L. Pessaint, A. Van Ry, K. Blade, J. Yalley-Ogunro, M. Cabus, R. Brown, A. Cook, E. Teow, H. Andersen, M. G. Lewis, D. A. Lauffenburger, G. Alter, D. H. Barouch, Correlates of protection against SARS-CoV-2 in rhesus macaques. Nature 590, 630–634 (2021). 33276369 10.1038/s41586-020-03041-6 PMC7906955

[17] E. G. Levin, Y. Lustig, C. Cohen, R. Fluss, V. Indenbaum, S. Amit, R. Doolman, K. Asraf, E. Mendelson, A. Ziv, C. Rubin, L. Freedman, Y. Kreiss, G. Regev-Yochay, Waning immune humoral response to BNT162b2 covid-19 vaccine over 6 Months. N. Engl. J. Med. 385, e84 (2021). 34614326 10.1056/NEJMoa2114583 PMC8522797

[18] D. Urlaub, N. Wolfsdorff, J. E. Hoffmann, S. Dorok, M. Hoffmann, M. Anft, N. Pieris, P. Gunther, B. Schaaf, U. Cassens, P. Brode, M. Claus, L. K. Picard, S. Wingert, S. Backes, D. Durak, N. Babel, S. Pohlmann, F. Renken, S. Raunser, C. Watzl, Neutralizing antibody responses 300 days after SARS-CoV-2 infection and induction of high antibody titers after vaccination. Eur. J. Immunol. 52, 810–815 (2022). 35247269 10.1002/eji.202149758 PMC9087412

[19] D. Cromer, M. Steain, A. Reynaldi, T. E. Schlub, A. K. Wheatley, J. A. Juno, S. J. Kent, J. A. Triccas, D. S. Khoury, M. P. Davenport, Neutralising antibody titres as predictors of protection against SARS-CoV-2 variants and the impact of boosting: A meta-analysis. Lancet Microbe 3, e52–e61 (2022). 34806056 10.1016/S2666-5247(21)00267-6 PMC8592563

[20] D. S. Khoury, D. Cromer, A. Reynaldi, T. E. Schlub, A. K. Wheatley, J. A. Juno, K. Subbarao, S. J. Kent, J. A. Triccas, M. P. Davenport, Neutralizing antibody levels are highly predictive of immune protection from symptomatic SARS-CoV-2 infection. Nat. Med. 27, 1205–1211 (2021). 34002089 10.1038/s41591-021-01377-8

[21] L. Perez-Alos, J. J. A. Armenteros, J. R. Madsen, C. B. Hansen, I. Jarlhelt, S. R. Hamm, L. D. Heftdal, M. M. Pries-Heje, D. L. Moller, K. Fogh, R. B. Hasselbalch, A. Rosbjerg, S. Brunak, E. Sorensen, M. A. H. Larsen, S. R. Ostrowski, R. Frikke-Schmidt, R. Bayarri-Olmos, L. M. Hilsted, K. K. Iversen, H. Bundgaard, S. D. Nielsen, P. Garred, Modeling of waning immunity after SARS-CoV-2 vaccination and influencing factors. Nat. Commun. 13, 1614 (2022). 35347129 10.1038/s41467-022-29225-4 PMC8960902

[22] K. S. Corbett, B. Flynn, K. E. Foulds, J. R. Francica, S. Boyoglu-Barnum, A. P. Werner, B. Flach, S. O’Connell, K. W. Bock, M. Minai, B. M. Nagata, H. Andersen, D. R. Martinez, A. T. Noe, N. Douek, M. M. Donaldson, N. N. Nji, G. S. Alvarado, D. K. Edwards, D. R. Flebbe, E. Lamb, N. A. Doria-Rose, B. C. Lin, M. K. Louder, S. O’Dell, S. D. Schmidt, E. Phung, L. A. Chang, C. Yap, J. M. Todd, L. Pessaint, A. Van Ry, S. Browne, J. Greenhouse, T. Putman-Taylor, A. Strasbaugh, T. A. Campbell, A. Cook, A. Dodson, K. Steingrebe, W. Shi, Y. Zhang, O. M. Abiona, L. Wang, A. Pegu, E. S. Yang, K. Leung, T. Zhou, I. T. Teng, A. Widge, I. Gordon, L. Novik, R. A. Gillespie, R. J. Loomis, J. I. Moliva, G. Stewart-Jones, S. Himansu, W. P. Kong, M. C. Nason, K. M. Morabito, T. J. Ruckwardt, J. E. Ledgerwood, M. R. Gaudinski, P. D. Kwong, J. R. Mascola, A. Carfi, M. G. Lewis, R. S. Baric, A. McDermott, I. N. Moore, N. J. Sullivan, M. Roederer, R. A. Seder, B. S. Graham, Evaluation of the mRNA-1273 vaccine against SARS-CoV-2 in nonhuman primates. N. Engl. J. Med. 383, 1544–1555 (2020). 32722908 10.1056/NEJMoa2024671 PMC7449230

[23] K. S. Corbett, M. Gagne, D. A. Wagner, S. O’Connell, S. R. Narpala, D. R. Flebbe, S. F. Andrew, R. L. Davis, B. Flynn, T. S. Johnston, C. D. Stringham, L. Lai, D. Valentin, A. Van Ry, Z. Flinchbaugh, A. P. Werner, J. I. Moliva, M. Sriparna, S. O’Dell, S. D. Schmidt, C. Tucker, A. Choi, M. Koch, K. W. Bock, M. Minai, B. M. Nagata, G. S. Alvarado, A. R. Henry, F. Laboune, C. A. Schramm, Y. Zhang, E. S. Yang, L. Wang, M. Choe, S. Boyoglu-Barnum, S. Wei, E. Lamb, S. T. Nurmukhambetova, S. J. Provost, M. M. Donaldson, J. Marquez, J. M. Todd, A. Cook, A. Dodson, A. Pekosz, E. Boritz, A. Ploquin, N. Doria-Rose, L. Pessaint, H. Andersen, K. E. Foulds, J. Misasi, K. Wu, A. Carfi, M. C. Nason, J. Mascola, I. N. Moore, D. K. Edwards, M. G. Lewis, M. S. Suthar, M. Roederer, A. McDermott, D. C. Douek, N. J. Sullivan, B. S. Graham, R. A. Seder, Protection against SARS-CoV-2 Beta variant in mRNA-1273 vaccine-boosted nonhuman primates. Science 374, 1343–1353 (2021). 34672695 10.1126/science.abl8912

[24] K. S. Corbett, M. C. Nason, B. Flach, M. Gagne, S. O’Connell, T. S. Johnston, S. N. Shah, V. V. Edara, K. Floyd, L. Lai, C. McDanal, J. R. Francica, B. Flynn, K. Wu, A. Choi, M. Koch, O. M. Abiona, A. P. Werner, J. I. Moliva, S. F. Andrew, M. M. Donaldson, J. Fintzi, D. R. Flebbe, E. Lamb, A. T. Noe, S. T. Nurmukhambetova, S. J. Provost, A. Cook, A. Dodson, A. Faudree, J. Greenhouse, S. Kar, L. Pessaint, M. Porto, K. Steingrebe, D. Valentin, S. Zouantcha, K. W. Bock, M. Minai, B. M. Nagata, R. van de Wetering, S. Boyoglu-Barnum, K. Leung, W. Shi, E. S. Yang, Y. Zhang, J. M. Todd, L. Wang, G. S. Alvarado, H. Andersen, K. E. Foulds, D. K. Edwards, J. R. Mascola, I. N. Moore, M. G. Lewis, A. Carfi, D. Montefiori, M. S. Suthar, A. McDermott, M. Roederer, N. J. Sullivan, D. C. Douek, B. S. Graham, R. A. Seder, Immune correlates of protection by mRNA-1273 vaccine against SARS-CoV-2 in nonhuman primates. Science 373, eabj0299 (2021). 34529476 10.1126/science.abj0299 PMC8449013

[25] M. Gagne, K. S. Corbett, B. J. Flynn, K. E. Foulds, D. A. Wagner, S. F. Andrew, J. M. Todd, C. C. Honeycutt, L. McCormick, S. T. Nurmukhambetova, M. E. Davis-Gardner, L. Pessaint, K. W. Bock, B. M. Nagata, M. Minai, A. P. Werner, J. I. Moliva, C. Tucker, C. G. Lorang, B. Zhao, E. McCarthy, A. Cook, A. Dodson, I. T. Teng, P. Mudvari, J. Roberts-Torres, F. Laboune, L. Wang, A. Goode, S. Kar, S. Boyoglu-Barnum, E. S. Yang, W. Shi, A. Ploquin, N. Doria-Rose, A. Carfi, J. R. Mascola, E. A. Boritz, D. K. Edwards, H. Andersen, M. G. Lewis, M. S. Suthar, B. S. Graham, M. Roederer, I. N. Moore, M. C. Nason, N. J. Sullivan, D. C. Douek, R. A. Seder, Protection from SARS-CoV-2 Delta one year after mRNA-1273 vaccination in rhesus macaques coincides with anamnestic antibody response in the lung. Cell 185, 113–130.e15 (2022). 34921774 10.1016/j.cell.2021.12.002 PMC8639396

[26] J. Yu, L. H. Tostanoski, L. Peter, N. B. Mercado, K. McMahan, S. H. Mahrokhian, J. P. Nkolola, J. Liu, Z. Li, A. Chandrashekar, D. R. Martinez, C. Loos, C. Atyeo, S. Fischinger, J. S. Burke, M. D. Slein, Y. Chen, A. Zuiani, F. J. N. Lelis, M. Travers, S. Habibi, L. Pessaint, A. Van Ry, K. Blade, R. Brown, A. Cook, B. Finneyfrock, A. Dodson, E. Teow, J. Velasco, R. Zahn, F. Wegmann, E. A. Bondzie, G. Dagotto, M. S. Gebre, X. He, C. Jacob-Dolan, M. Kirilova, N. Kordana, Z. Lin, L. F. Maxfield, F. Nampanya, R. Nityanandam, J. D. Ventura, H. Wan, Y. Cai, B. Chen, A. G. Schmidt, D. R. Wesemann, R. S. Baric, G. Alter, H. Andersen, M. G. Lewis, D. H. Barouch, DNA vaccine protection against SARS-CoV-2 in rhesus macaques. Science 369, 806–811 (2020). 32434945 10.1126/science.abc6284 PMC7243363

[27] C. M. Fovet, C. Pimienta, M. Galhaut, F. Relouzat, N. Nunez, M. Cavarelli, Q. Sconosciuti, N. Dhooge, I. Marzinotto, V. Lampasona, M. Tolazzi, G. Scarlatti, R. H. T. Fang, T. Naninck, N. Dereuddre-Bosquet, J. Van Wassenhove, A. S. Gallouet, P. Maisonnasse, R. Le Grand, E. Menu, N. Seddiki, A case study to dissect immunity to SARS-CoV-2 in a neonate nonhuman primate model. Front. Immunol. 13, 855230 (2022). 35603150 10.3389/fimmu.2022.855230 PMC9114777

[28] C. Garrido, A. D. Curtis II, M. Dennis, S. H. Pathak, H. Gao, D. Montefiori, M. Tomai, C. B. Fox, P. A. Kozlowski, T. Scobey, J. E. Munt, M. L. Mallory, P. T. Saha, M. G. Hudgens, L. C. Lindesmith, R. S. Baric, O. M. Abiona, B. Graham, K. S. Corbett, D. Edwards, A. Carfi, G. Fouda, K. K. A. Van Rompay, K. De Paris, S. R. Permar, SARS-CoV-2 vaccines elicit durable immune responses in infant rhesus macaques. Sci. Immunol. 6, eabj3684 (2021). 34131024 10.1126/sciimmunol.abj3684 PMC8774290

[29] V. J. Munster, F. Feldmann, B. N. Williamson, N. van Doremalen, L. Perez-Perez, J. Schulz, K. Meade-White, A. Okumura, J. Callison, B. Brumbaugh, V. A. Avanzato, R. Rosenke, P. W. Hanley, G. Saturday, D. Scott, E. R. Fischer, E. de Wit, Respiratory disease in rhesus macaques inoculated with SARS-CoV-2. Nature 585, 268–272 (2020). 32396922 10.1038/s41586-020-2324-7 PMC7486227

[30] K. K. A. Van Rompay, K. J. Olstad, R. L. Sammak, J. Dutra, J. K. Watanabe, J. L. Usachenko, R. Immareddy, A. Verma, Y. Shaan Lakshmanappa, B. A. Schmidt, J. W. Roh, S. R. Elizaldi, A. M. Allen, F. Muecksch, J. C. C. Lorenzi, S. Lockwood, R. E. Pollard, J. L. Yee, P. B. Nham, A. Ardeshir, J. D. Deere, J. Patterson, Q. Dang, T. Hatziioannou, P. D. Bieniasz, S. S. Iyer, D. J. Hartigan-O’Connor, M. C. Nussenzweig, J. R. Reader, Early treatment with a combination of two potent neutralizing antibodies improves clinical outcomes and reduces virus replication and lung inflammation in SARS-CoV-2 infected macaques. PLOS Pathog. 17, e1009688 (2021). 34228761 10.1371/journal.ppat.1009688 PMC8284825

[31] A. Verma, C. E. Hawes, Y. S. Lakshmanappa, J. W. Roh, B. A. Schmidt, J. Dutra, W. Louie, H. Liu, Z. M. Ma, J. K. Watanabe, J. L. Usachenko, R. Immareddy, R. L. Sammak, R. Pollard, J. R. Reader, K. J. Olstad, L. L. Coffey, P. A. Kozlowski, D. J. Hartigan-O’Connor, M. Nussenzweig, K. K. A. Van Rompay, J. H. Morrison, S. S. Iyer, Monoclonal antibodies protect aged rhesus macaques from SARS-CoV-2-induced immune activation and neuroinflammation. Cell Rep. 37, 109942 (2021). 34706272 10.1016/j.celrep.2021.109942 PMC8523485

[32] K. L. Oxford, M. G. A. Dela Pena-Ponce, K. Jensen, M. K. Eberhardt, A. Spinner, K. K. Van Rompay, J. Rigdon, K. R. Mollan, V. V. Krishnan, M. G. Hudgens, P. A. Barry, K. De Paris, The interplay between immune maturation, age, chronic viral infection and environment. Immun. Ageing 12, 3 (2015). 25991918 10.1186/s12979-015-0030-3 PMC4436863

[33] K. Jensen, R. Nabi, K. K. Van Rompay, S. Robichaux, J. D. Lifson, M. Piatak Jr., W. R. Jacobs Jr., G. Fennelly, D. Canfield, K. R. Mollan, M. G. Hudgens, M. H. Larsen, A. M. Amedee, P. A. Kozlowski, K. De Paris, Vaccine-elicited mucosal and systemic antibody responses are associated with reduced simian immunodeficiency viremia in infant rhesus macaques. J. Virol. 90, 7285–7302 (2016). 27252535 10.1128/JVI.00481-16 PMC4984660

[34] K. K. Van Rompay, R. P. Singh, L. L. Brignolo, J. R. Lawson, K. A. Schmidt, B. Pahar, D. R. Canfield, R. P. Tarara, D. L. Sodora, N. Bischofberger, M. L. Marthas, The clinical benefits of tenofovir for simian immunodeficiency virus-infected macaques are larger than predicted by its effects on standard viral and immunologic parameters. J. Acquir. Immune Defic. Syndr. 36, 900–914 (2004). 15220696 10.1097/00126334-200408010-00003

[35] K. M. Merino, N. Slisarenko, J. M. Taylor, K. P. Falkenstein, M. H. Gilbert, R. P. Bohm, J. L. Blanchard, A. Ardeshir, E. S. Didier, W. K. Kim, M. J. Kuroda, Clinical and immunological metrics during pediatric rhesus macaque development. Front. Pediatr. 8, 388 (2020). 32766187 10.3389/fped.2020.00388 PMC7378395

[36] K. L. Chiou, M. J. Montague, E. A. Goldman, M. M. Watowich, S. N. Sams, J. Song, J. E. Horvath, K. N. Sterner, A. V. Ruiz-Lambides, M. I. Martinez, J. P. Higham, L. J. N. Brent, M. L. Platt, N. Snyder-Mackler, Rhesus macaques as a tractable physiological model of human ageing. Philos. Trans. R. Soc. Lond. B Biol. Sci. 375, 20190612 (2020). 32951555 10.1098/rstb.2019.0612 PMC7540946

[37] S. P. Kasturi, P. A. Kozlowski, H. I. Nakaya, M. C. Burger, P. Russo, M. Pham, Y. Kovalenkov, E. L. Silveira, C. Havenar-Daughton, S. L. Burton, K. M. Kilgore, M. J. Johnson, R. Nabi, T. Legere, Z. J. Sher, X. Chen, R. R. Amara, E. Hunter, S. E. Bosinger, P. Spearman, S. Crotty, F. Villinger, C. A. Derdeyn, J. Wrammert, B. Pulendran, Adjuvanting a simian immunodeficiency virus vaccine with toll-like receptor ligands encapsulated in nanoparticles induces persistent antibody responses and enhanced protection in TRIM5α restrictive macaques. J. Virol. 91, e01844–16 (2017). 27928002 10.1128/JVI.01844-16 PMC5286877

[38] M. Pino, T. Abid, S. Pereira Ribeiro, V. V. Edara, K. Floyd, J. C. Smith, M. B. Latif, G. Pacheco-Sanchez, D. Dutta, S. Wang, S. Gumber, S. Kirejczyk, J. Cohen, R. L. Stammen, S. M. Jean, J. S. Wood, F. Connor-Stroud, J. Pollet, W. H. Chen, J. Wei, B. Zhan, J. Lee, Z. Liu, U. Strych, N. Shenvi, K. Easley, D. Weiskopf, A. Sette, J. Pollara, D. Mielke, H. Gao, N. Eisel, C. C. LaBranche, X. Shen, G. Ferrari, G. D. Tomaras, D. C. Montefiori, R. P. Sekaly, T. H. Vanderford, M. A. Tomai, C. B. Fox, M. S. Suthar, P. A. Kozlowski, P. J. Hotez, M. Paiardini, M. E. Bottazzi, S. P. Kasturi, A yeast expressed RBD-based SARS-CoV-2 vaccine formulated with 3M-052-alum adjuvant promotes protective efficacy in non-human primates. Sci. Immunol. 6, eabh3634 (2021). 34266981 10.1126/sciimmunol.abh3634 PMC9119307

[39] L. Azzi, D. Dalla Gasperina, G. Veronesi, M. Shallak, G. Ietto, D. Iovino, A. Baj, F. Gianfagna, V. Maurino, D. Focosi, F. Maggi, M. M. Ferrario, F. Dentali, G. Carcano, A. Tagliabue, L. S. Maffioli, R. S. Accolla, G. Forlani, Mucosal immune response in BNT162b2 COVID-19 vaccine recipients. EBioMedicine 75, 103788 (2022). 34954658 10.1016/j.ebiom.2021.103788 PMC8718969

[40] A. Lombardi, E. Trombetta, A. Cattaneo, V. Castelli, E. Palomba, M. Tirone, D. Mangioni, G. Lamorte, M. Manunta, D. Prati, F. Ceriotti, R. Gualtierotti, G. Costantino, S. Aliberti, V. Scaravilli, G. Grasselli, A. Gori, L. Porretti, A. Bandera, Early phases of COVID-19 are characterized by a reduction in lymphocyte populations and the presence of atypical monocytes. Front. Immunol. 11, 560330 (2020). 33362757 10.3389/fimmu.2020.560330 PMC7756112

[41] K. K. A. Van Rompay, K. J. Olstad, R. L. Sammak, J. Dutra, J. K. Watanabe, J. L. Usachenko, R. Immareddy, J. W. Roh, A. Verma, Y. Shaan Lakshmanappa, B. A. Schmidt, C. Di Germanio, N. Rizvi, H. Liu, Z. M. Ma, M. Stone, G. Simmons, L. J. Dumont, A. M. Allen, S. Lockwood, R. E. Pollard, R. Ramiro de Assis, J. L. Yee, P. B. Nham, A. Ardeshir, J. D. Deere, A. Jain, P. L. Felgner, L. L. Coffey, S. S. Iyer, D. J. Hartigan-O’Connor, M. P. Busch, J. R. Reader, Early post-infection treatment of SARS-CoV-2 infected macaques with human convalescent plasma with high neutralizing activity had no antiviral effects but moderately reduced lung inflammation. PLOS Pathog. 18, e1009925 (2022). 35443018 10.1371/journal.ppat.1009925 PMC9060337

[42] Y. Shaan Lakshmanappa, S. R. Elizaldi, J. W. Roh, B. A. Schmidt, T. D. Carroll, K. D. Weaver, J. C. Smith, A. Verma, J. D. Deere, J. Dutra, M. Stone, S. Franz, R. L. Sammak, K. J. Olstad, J. Rachel Reader, Z. M. Ma, N. K. Nguyen, J. Watanabe, J. Usachenko, R. Immareddy, J. L. Yee, D. Weiskopf, A. Sette, D. Hartigan-O’Connor, S. J. McSorley, J. H. Morrison, N. K. Tran, G. Simmons, M. P. Busch, P. A. Kozlowski, K. K. A. Van Rompay, C. J. Miller, S. S. Iyer, SARS-CoV-2 induces robust germinal center CD4 T follicular helper cell responses in rhesus macaques. Nat. Commun. 12, 541 (2021). 33483492 10.1038/s41467-020-20642-x PMC7822826

[43] *Coronavirus (COVID-19) Update: FDA Authorizes Moderna and Pfizer-BioNTech COVID-19 Vaccines for Children Down to 6 Months of Age* (2022).

[44] K. W. Cohen, S. L. Linderman, Z. Moodie, J. Czartoski, L. Lai, G. Mantus, C. Norwood, L. E. Nyhoff, V. V. Edara, K. Floyd, S. C. De Rosa, H. Ahmed, R. Whaley, S. N. Patel, B. Prigmore, M. P. Lemos, C. W. Davis, S. Furth, J. B. O’Keefe, M. P. Gharpure, S. Gunisetty, K. Stephens, R. Antia, V. I. Zarnitsyna, D. S. Stephens, S. Edupuganti, N. Rouphael, E. J. Anderson, A. K. Mehta, J. Wrammert, M. S. Suthar, R. Ahmed, M. J. McElrath, Longitudinal analysis shows durable and broad immune memory after SARS-CoV-2 infection with persisting antibody responses and memory B and T cells. Cell Rep. Med. 2, 100354 (2021). 34250512 10.1016/j.xcrm.2021.100354 PMC8253687

[45] A. Pegu, S. E. O’Connell, S. D. Schmidt, S. O’Dell, C. A. Talana, L. Lai, J. Albert, E. Anderson, H. Bennett, K. S. Corbett, B. Flach, L. Jackson, B. Leav, J. E. Ledgerwood, C. J. Luke, M. Makowski, M. C. Nason, P. C. Roberts, M. Roederer, P. A. Rebolledo, C. A. Rostad, N. G. Rouphael, W. Shi, L. Wang, A. T. Widge, E. S. Yang; mRNA-1273 Study Group, J. H. Beigel, B. S. Graham, J. R. Mascola, M. S. Suthar, A. B. McDermott, N. A. Doria-Rose, J. Arega, J. H. Beigel, W. Buchanan, M. Elsafy, B. Hoang, R. Lampley, A. Kolhekar, H. Koo, C. Luke, M. Makhene, S. Nayak, R. Pikaart-Tautges, P. C. Roberts, J. Russell, E. Sindall, J. Albert, P. Kunwar, M. Makowski, E. J. Anderson, A. Bechnak, M. Bower, A. F. Camacho-Gonzalez, M. Collins, A. Drobeniuc, V. V. Edara, S. Edupuganti, K. Floyd, T. Gibson, C. M. G. Ackerley, B. Johnson, S. Kamidani, C. Kao, C. Kelley, L. Lai, H. Macenczak, M. P. McCullough, E. Peters, V. K. Phadke, P. A. Rebolledo, C. A. Rostad, N. Rouphael, E. Scherer, A. Sherman, K. Stephens, M. S. Suthar, M. Teherani, J. Traenkner, J. Winston, I. Yildirim, L. Barr, J. Benoit, B. Carste, J. Choe, M. Dunstan, R. Erolin, J. Ffitch, C. Fields, L. A. Jackson, E. Kiniry, S. Lasicka, S. Lee, M. Nguyen, S. Pimienta, J. Suyehira, M. Witte, H. Bennett, N. E. Altaras, A. Carfi, M. Hurley, B. Leav, R. Pajon, W. Sun, T. Zaks, R. N. Coler, S. E. Larsen, K. M. Neuzil, L. C. Lindesmith, D. R. Martinez, J. Munt, M. Mallory, C. Edwards, R. S. Baric, N. M. Berkowitz, E. A. Boritz, K. Carlton, K. S. Corbett, P. Costner, A. Creanga, N. A. Doria-Rose, D. C. Douek, B. Flach, M. Gaudinski, I. Gordon, B. S. Graham, L. Holman, J. E. Ledgerwood, K. Leung, B. C. Lin, M. K. Louder, J. R. Mascola, A. B. McDermott, K. M. Morabito, L. Novik, S. O’Connell, S. O’Dell, M. Padilla, A. Pegu, S. D. Schmidt, W. Shi, P. A. Swanson 2nd, C. A. Talana, L. Wang, A. T. Widge, E. S. Yang, Y. Zhang, J. D. Chappell, M. R. Denison, T. Hughes, X. Lu, A. J. Pruijssers, L. J. Stevens, C. M. Posavad, M. Gale Jr., V. Menachery, P. Y. Shi, Durability of mRNA-1273 vaccine-induced antibodies against SARS-CoV-2 variants. Science 373, 1372–1377 (2021). 34385356 10.1126/science.abj4176 PMC8691522

[46] N. Doria-Rose, M. S. Suthar, M. Makowski, S. O’Connell, A. B. McDermott, B. Flach, J. E. Ledgerwood, J. R. Mascola, B. S. Graham, B. C. Lin, S. O’Dell, S. D. Schmidt, A. T. Widge, V. V. Edara, E. J. Anderson, L. Lai, K. Floyd, N. G. Rouphael, V. Zarnitsyna, P. C. Roberts, M. Makhene, W. Buchanan, C. J. Luke, J. H. Beigel, L. A. Jackson, K. M. Neuzil, H. Bennett, B. Leav, J. Albert, P. Kunwar; mRNA-1273 Study Group, Antibody persistence through 6 Months after the second dose of mRNA-1273 vaccine for Covid-19. N. Engl. J. Med. 384, 2259–2261 (2021). 33822494 10.1056/NEJMc2103916 PMC8524784

[47] Z. Q. Toh, R. A. Higgins, L. A. H. Do, K. Rautenbacher, F. L. Mordant, K. Subbarao, K. Dohle, J. Nguyen, A. C. Steer, S. Tosif, N. W. Crawford, K. Mulholland, P. V. Licciardi, Persistence of SARS-CoV-2-specific IgG in children 6 months after infection, Australia. Emerg. Infect. Dis. 27, 2233–2235 (2021). 34016252 10.3201/eid2708.210965 PMC8314814

[48] X. Tian, Z. Bai, Y. Cao, H. Liu, D. Liu, W. Liu, J. Li, Evaluation of clinical and immune responses in recovered children with mild COVID-19. Viruses 14, 85 (2022). 35062289 10.3390/v14010085 PMC8779549

[49] T. W. Barnes, J. Schulte-Pelkum, L. Steller, D. Filchtinski, R. Jenness, M. R. Williams, C. Kober, S. Manni, T. Hauser, A. Hahn, U. Kalina, T. L. Simon, P. Schuetz, N. J. Roth, Determination of neutralising anti-SARS-CoV-2 antibody half-life in COVID-19 convalescent donors. Clin. Immunol. 232, 108871 (2021). 34619377 10.1016/j.clim.2021.108871 PMC8489294

[50] G. Trapani, G. Verlato, E. Bertino, G. Maiocco, R. Vesentini, A. Spadavecchia, A. Dessi, V. Fanos, Long COVID-19 in children: An Italian cohort study. Ital. J. Pediatr. 48, 83 (2022). 35659358 10.1186/s13052-022-01282-x PMC9163526

[51] D. Beckman, A. Bonillas, G. Diniz, S. Ott, J. W. Roh, S. R. Elizaldi, B. A. Schmidt, R. L. Sammak, K. K. Van Rompay, S. L. Iyer, J. H. Morrison, SARS-CoV-2 infects neurons and induces neuroinflammation in a non-human primate model of COVID-19. Cell Rep. 41, 111573 (2022). 36288725 10.1016/j.celrep.2022.111573 PMC9554328

[52] I. Rutkai, M. G. Mayer, L. M. Hellmers, B. Ning, Z. Huang, C. J. Monjure, C. Coyne, R. Silvestri, N. Golden, K. Hensley, K. Chandler, G. Lehmicke, G. J. Bix, N. J. Maness, K. Russell-Lodrigue, T. Y. Hu, C. J. Roy, R. V. Blair, R. Bohm, L. A. Doyle-Meyers, J. Rappaport, T. Fischer, Neuropathology and virus in brain of SARS-CoV-2 infected non-human primates. Nat. Commun. 13, 1745 (2022). 35365631 10.1038/s41467-022-29440-z PMC8975902

[53] K. A. Earle, D. M. Ambrosino, A. Fiore-Gartland, D. Goldblatt, P. B. Gilbert, G. R. Siber, P. Dull, S. A. Plotkin, Evidence for antibody as a protective correlate for COVID-19 vaccines. Vaccine 39, 4423–4428 (2021). 34210573 10.1016/j.vaccine.2021.05.063 PMC8142841

[54] A. B. Vogel, I. Kanevsky, Y. Che, K. A. Swanson, A. Muik, M. Vormehr, L. M. Kranz, K. C. Walzer, S. Hein, A. Guler, J. Loschko, M. S. Maddur, A. Ota-Setlik, K. Tompkins, J. Cole, B. G. Lui, T. Ziegenhals, A. Plaschke, D. Eisel, S. C. Dany, S. Fesser, S. Erbar, F. Bates, D. Schneider, B. Jesionek, B. Sanger, A. K. Wallisch, Y. Feuchter, H. Junginger, S. A. Krumm, A. P. Heinen, P. Adams-Quack, J. Schlereth, S. Schille, C. Kroner, R. de la Caridad Guimil Garcia, T. Hiller, L. Fischer, R. S. Sellers, S. Choudhary, O. Gonzalez, F. Vascotto, M. R. Gutman, J. A. Fontenot, S. Hall-Ursone, K. Brasky, M. C. Griffor, S. Han, A. A. H. Su, J. A. Lees, N. L. Nedoma, E. H. Mashalidis, P. V. Sahasrabudhe, C. Y. Tan, D. Pavliakova, G. Singh, C. Fontes-Garfias, M. Pride, I. L. Scully, T. Ciolino, J. Obregon, M. Gazi, R. Carrion Jr., K. J. Alfson, W. V. Kalina, D. Kaushal, P. Y. Shi, T. Klamp, C. Rosenbaum, A. N. Kuhn, O. Tureci, P. R. Dormitzer, K. U. Jansen, U. Sahin, BNT162b vaccines protect rhesus macaques from SARS-CoV-2. Nature 592, 283–289 (2021). 33524990 10.1038/s41586-021-03275-y

[55] U. Sahin, A. Muik, E. Derhovanessian, I. Vogler, L. M. Kranz, M. Vormehr, A. Baum, K. Pascal, J. Quandt, D. Maurus, S. Brachtendorf, V. Lorks, J. Sikorski, R. Hilker, D. Becker, A. K. Eller, J. Grutzner, C. Boesler, C. Rosenbaum, M. C. Kuhnle, U. Luxemburger, A. Kemmer-Bruck, D. Langer, M. Bexon, S. Bolte, K. Kariko, T. Palanche, B. Fischer, A. Schultz, P. Y. Shi, C. Fontes-Garfias, J. L. Perez, K. A. Swanson, J. Loschko, I. L. Scully, M. Cutler, W. Kalina, C. A. Kyratsous, D. Cooper, P. R. Dormitzer, K. U. Jansen, O. Tureci, COVID-19 vaccine BNT162b1 elicits human antibody and TH1 T cell responses. Nature 586, 594–599 (2020). 32998157 10.1038/s41586-020-2814-7

[56] D. M. Skowronski, G. De Serres, Safety and Efficacy of the BNT162b2 mRNA Covid-19 Vaccine. N. Engl. J. Med. 384, 1576–1577 (2021). 33596348 10.1056/NEJMc2036242

[57] A. Tarke, C. H. Coelho, Z. Zhang, J. M. Dan, E. D. Yu, N. Methot, N. I. Bloom, B. Goodwin, E. Phillips, S. Mallal, J. Sidney, G. Filaci, D. Weiskopf, R. da Silva Antunes, S. Crotty, A. Grifoni, A. Sette, SARS-CoV-2 vaccination induces immunological T cell memory able to cross-recognize variants from Alpha to Omicron. Cell 185, 847–859.e11 (2022). 35139340 10.1016/j.cell.2022.01.015 PMC8784649

[58] S. Kumar, K. Karuppanan, G. Subramaniam, Omicron (BA.1) and sub-variants (BA.1.1, BA.2 and BA.3) of SARS-CoV-2 spike infectivity and pathogenicity: a comparative sequence and structural-based computational assessment. J. Med. Virol. 94, 4780–4791 (2022). 35680610 10.1002/jmv.27927 PMC9347785

[59] B. Phillips, K. K. A. Van Rompay, J. Rodriguez-Nieves, C. Lorin, M. Koutsoukos, M. Tomai, C. B. Fox, J. Eudailey, M. Dennis, S. M. Alam, M. Hudgens, G. Fouda, J. Pollara, A. Moody, X. Shen, G. Ferrari, S. Permar, K. De Paris, Adjuvant-dependent enhancement of HIV env-specific antibody responses in infant rhesus macaques. J. Virol. 92, e01051-18 (2018). 30089691 10.1128/JVI.01051-18 PMC6158427

[60] D. J. Dowling, S. D. van HAren, A. Schneid, I. Bergelson, D. Kim, C. J. Mancuso, W. Foppen, A. Ozonoff, L. Fresh, T. B. Theriot, A. A. Lackner, R. N. Ficherova, D. Smirnov, J. P. Vasilakos, J. M. Beaurline, M. A. Tomai, C. C. Midkiff, X. Alvarez, J. L. Blanchard, M. H. Gilbert, P. Aye, O. Levy, TLR7/8 adjuvant overcomes newborn hyporesponsiveness to pneumococcal conjugate vaccine at birth. JCI Insight 2, e91020 (2017). 28352660 10.1172/jci.insight.91020 PMC5360187

[61] N. K. Routhu, N. Cheedarla, V. S. Bollimpelli, S. Gangadhara, V. V. Edara, L. Lai, A. Sahoo, A. Shiferaw, T. M. Styles, K. Floyd, S. Fischinger, C. Atyeo, S. A. Shin, S. Gumber, S. Kirejczyk, K. H. Dinnon III, P. Y. Shi, V. D. Menachery, M. Tomai, C. B. Fox, G. Alter, T. H. Vanderford, L. Gralinski, M. S. Suthar, R. R. Amara, SARS-CoV-2 RBD trimer protein adjuvanted with Alum-3M-052 protects from SARS-CoV-2 infection and immune pathology in the lung. Nat. Commun. 12, 3587 (2021). 34117252 10.1038/s41467-021-23942-y PMC8196016

[62] I. G. Sakala, K. M. Eichinger, N. Petrovsky, Neonatal vaccine effectiveness and the role of adjuvants. Expert Rev. Clin. Immunol. 15, 869–878 (2019). 31293189 10.1080/1744666X.2019.1642748 PMC6678067

[63] J. Pollet, W. H. Chen, U. Strych, Recombinant protein vaccines, a proven approach against coronavirus pandemics. Adv. Drug Deliv. Rev. 170, 71–82 (2021). 33421475 10.1016/j.addr.2021.01.001 PMC7788321

[64] N. Percie du Sert, V. Hurst, A. Ahluwalia, S. Alam, M. T. Avey, M. Baker, W. J. Browne, A. Clark, I. C. Cuthill, U. Dirnagl, M. Emerson, P. Garner, S. T. Holgate, D. W. Howells, N. A. Karp, S. E. Lazic, K. Lidster, C. J. MacCallum, M. Macleod, E. J. Pearl, O. H. Petersen, F. Rawle, P. Reynolds, K. Rooney, E. S. Sena, S. D. Silberberg, T. Steckler, H. Wurbel, The ARRIVE guidelines 2.0: Updated guidelines for reporting animal research. PLoS Biol. 18, e3000410 (2020). 32663219 10.1371/journal.pbio.3000410 PMC7360023

[65] M. G. Dela Pena-Ponce, J. Rodriguez-Nieves, J. Bernhardt, R. Tuck, N. Choudhary, M. Mengual, K. R. Mollan, M. G. Hudgens, S. Peter-Wohl, K. De Paris, Increasing JAK/STAT signaling function of infant CD4^+^ T cells during the first year of life. Front. Pediatr. 5, 15 (2017). 28271056 10.3389/fped.2017.00015 PMC5318443

