# Supplementary material for: Infant rhesus macaques immunized against SARS-CoV-2 are protected against heterologous virus challenge one year later
Source: Sci Transl Med. 2022 Dec 1;14:eadd6383. doi: 10.1126/scitranslmed.add6383 (PMC9765459; doi:10.1126/scitranslmed.add6383)
Supplement: Supplementary file 1 — Materials and Methods Figs. S1 to S19 Tables S1 to S11 [file scitranslmed.add6383_sm.pdf]

Science Translational Medicine
AAAS

Supplementary Materials for

Infant rhesus macaques immunized against SARS-CoV-2 are protected against heterologous virus challenge one year later

Emma C. Milligan *et al.*

Corresponding author: Kristina De Paris, abelk@med.unc.edu

Sci. Transl. Med. **14**, eadd6383 (2022)
DOI: 10.1126/scitranslmed.add6383

The PDF file includes:

Materials and Methods
Figs. S1 to S19
Tables S1 to S11
Legend for data file S1

Other Supplementary Material for this manuscript includes the following:

MDAR Reproducibility Checklist
Data file S1

METHODS

Sample collection and processing

Blood was collected by peripheral venipuncture. Complete blood counts were performed on EDTA-anticoagulated blood samples, with electronic cell counts performed on a Pentra 60C+ analyzer (ABX Diagnostics) or Vet abc (SCIL Animal Care); differential cell counts were determined manually. EDTA anti-coagulated blood was used for the collection of plasma and peripheral mononuclear cells (PBMCs) as described (*28*). PBMCs were used fresh or stored in liquid nitrogen. Blood tubes without coagulant were also collected for processing by centrifugation (900xg for 10 minutes) for serum. Plasma and serum aliquots were stored at -70°C until further processing.

For antibody analysis, saliva samples were collected with Merocel sponges (Beaver Visitec), and nasal secretions were collected with Keracel sponges (Beaver Visitec) after instilling of 250 µl PBS in the nostril and then using the sponge tip to absorb the fluid. For viral RNA analysis, nasopharyngeal, oropharyngeal and rectal secretions were collected with FLOQSwabs (Copan), placed in a vial with DNA/RNA Shield solution (Zymo Research), and stored at -70°C until further processing. Nasal secretions for antibody analysis and viral RNA analysis were always collected from opposite nostrils to avoid interference.

Bronchoalveolar lavages (BAL) were performed using a 20F rubber feeding tube with instillation of 20 mL sterile physiologic saline followed by aspiration with a syringe. BAL samples were centrifuged. The BAL cell pellet, together with 0.5 mL of supernatant, was then mixed with 1.5 mL of TRIzol-LS (Thermo Fisher Scientific) and cryopreserved at -70°C. Additional aliquots of BAL supernatant were also immediately cryopreserved. At day 7 after challenge, animals were euthanized, and a full necropsy was performed for tissue collection, including snap-frozen tissue samples, and fixed tissues for histopathology.

Measurement of cytokines and chemokines in plasma

Plasma cytokines and chemokines were measured using the Cytokine 29-Plex Monkey Panel (Invitrogen). This is a multiplex microbead fluorescent assay utilizing the Luminex™ platform. The assay was run according to manufacturer’s instructions.

Serum biochemistry

Biochemistry analysis on serum samples from day of challenge onwards was performed using Piccolo BioChemistry Plus disks that were run on the Piccolo Xpress Chemistry Analyzer (Abbott). Biochemistry analysis was performed according to the manufacturer's instructions. This panel includes alanine aminotransferase (ALT), albumin, alkaline phosphatase (ALP), amylase, aspartate aminotransferase (AST), C-reactive protein (CRP), calcium, creatinine, gamma glutamyltransferase (GGT), glucose, total protein, blood urea nitrogen (BUN), and uric acid.

Plasma IgG ELISA

IgG binding was measured in plasma before and after challenge using an enzyme-linked immunosorbent assay (ELISA) as previously described (*28*). Briefly, 384-well plates were coated overnight with 2 µg/mL of trimer spike (S) protein from the original D614G, B.1.617.2, and B.1.1.529 variants (Sino Biological). Plates were then blocked with assay diluent (phosphate-buffered saline containing 4% whey, 15% normal goat serum, and 0.5% Tween 20). Eleven serial 4-fold dilutions of plasma starting at 1:10 were added to the plates and incubated for 1 hour, followed by detection with horseradish peroxidase (HRP)-conjugated mouse anti-monkey IgG (Southern Biotech) at 1:4000 dilution (0.1375 µg/mL). The plates were developed by using an ABTS-2 [2,2'-azinobis(3-ethylbenzthiazolinesulfonic acid)] peroxidase substrate system (KPL) and absorbance was read at 450 nM with a Spectramax Microplate Reader (Molecular Devices). As positive controls, human monoclonal antibodies had to be used due to the lack of a rhesus macaque-specific IgG reagent of known concentration. For the D614G and B.1.617.2 assays, we used D001 (Sino Biological). Since D001 does not bind to B.1.1.529 S protein, we used M265 (Acrobiosystems). Goat Anti-human IgG HRP (Jackson Immunoresearch) was used for detection of the human mAb.

Measurement of IgG and IgA in mucosal secretions

Saliva was collected with absorbent Merocel sponges (Beaver Visitec) by placing a sponge between the cheek and gum in the back of the mouth for 5 minutes. Nasal secretions were collected with Keracel sponges (Beaver Visitec) by pipetting 100 µL of 1X PBS into a nostril, briefly pressing the nostril shut and massaging it, then inserting a dry Keracel sponge. Secretions were eluted from sponges as previously described (*28*). A customized binding antibody multiplex assay (BAMA) was used to measure IgG and IgA antibodies to the SARS-CoV-2 Delta (B.1.617.2) receptor-binding domain (RBD) (Sino Biologicals) as described (*28*). Briefly, serial dilutions of standard and centrifuged secretions were mixed overnight with RBD labeled beads. Before

performing IgA assays, secretions were depleted of IgG (*66*). The following day, beads were washed and reacted with biotinylated goat anti-human IgG (SouthernBiotech) or anti-monkey IgA (Rockland) followed by avidin-phycoerythrin (PE; Southern Biotech). Fluorescent intensity was measured with a BioRad BioPlex 200. Concentrations of antibody interpolated from standard curves were divided by the total IgG or IgA measured by ELISA as described (*33, 66*).

Binding antibody multiplex assay (BAMA) to measure antibody breadth

Purified S and N Protein of SARS-CoV-2 variants were purchased from Sino Biological and conjugated to BioRad Magplex magnetic COOH beads (BioRad) **(table S10)**. NHP serum samples were heat-inactivated at 56°C for 30 minutes and diluted 1:2000 in BAMA diluent (1x PBS, 1% Non-Fat Dry Milk Powder, 5% Normal Goat Serum, and 0.05% Tween-20). Samples were incubated with beads for 30 minutes while shaking at 180 rpm at room temperature. PE-labelled goat anti-Rhesus IgG (Southern Biotech) was used to detect rhesus IgG, human IgG positive control were probed using a goat-anti human IgG-PE (Southern Biotech). Secondary antibodies were diluted to 2 µg/mL in BAMA diluent and incubated with samples for 30 minutes rotating at 180 rpm at room temperature. Plates were read on the BioRad Bioplex 200 instrument. To assess background, a blank well of conjugated beads and a single unconjugated bead diluted in assay buffer was included on each plate.

To calculate the breadth score, the mean fluorescent intensity (MFI) from blank wells was subtracted from the experimental MFI signal (background correction) and log transformed. The negative cutoff was calculated by taking the log of the mean of MFI detected in plasma samples prior to immunization (week 52) and control animals on day 0 of challenge (total of n=24) for each antigen in the assay. The magnitude of antibody binding to each antigen was calculated by taking the ratio of the background corrected, log transformed MFI of each sample and the negative cutoff; ratios >1 were assigned a score of 1. The breadth score for each animal was then calculated by multiplying the sum of the magnitudes by the average frequency for all S antigens.

Pseudovirus Antibody Neutralization Assay

SARS-CoV-2 neutralization was assessed with S-pseudotyped viruses (D614G strain and B.1.617.2) in 293T/ACE2 cells (provided by Drs. Farzan and Mu, Scripps Florida, Jupiter, FL) as a function of reductions in luciferase (Luc) reporter activity as described previously (*28*). Briefly, a pre-titrated dose of pseudovirus was incubated with 8 serial 5-fold dilutions of serum samples in duplicate in a total volume of 150 µL for 1 hour at 37°C in 96-well flat bottom poly-L-lysine-

coated culture plates (Corning Biocoat). Cells were suspended using TrypLE Select Enzyme solution (Thermo Fisher Scientific) and immediately added to all wells (10,000 cells in 100 µL of growth medium per well). One set of 8 control wells received cells + virus (virus control) and another set of 8 wells received cells only (background control). After 66 to 72 hours of incubation, medium was removed by gentle aspiration and 30 µL of Promega 1X lysis buffer was added to all wells. After a 10-minute incubation at room temperature, 100 µL of Bright-Glo luciferase reagent was added to all wells. After 1 to 2 minutes, 110 µL of the cell lysate was transferred to a black/white plate (Perkin-Elmer). Luminescence was measured using a PerkinElmer Life Sciences, Model Victor2 Luminometer. Neutralization titers are the serum dilution at which relative luminescence units (RLU) were reduced by either 50% (ID_{50}) or 80% (ID_{80}) compared to virus control wells after subtraction of background RLUs. Serum samples were heat-inactivated for 30 minutes at 56°C prior to assay.

Whole Virus Neutralization Assay

Neutralization of SARS-CoV-2 nanoLUC carrying the D614G or B.1.617.2 mutation was assessed as described in Hou et al with modifications (*67*), as we reported previously (*28*). Briefly, under BSL-2 containment, serially diluted serum samples at 8 dilutions were incubated for one hour with SARS-CoV-2 D614G nanoLUC virus at 37°C and 5% CO_2. After incubation, the virus/antibody mixtures were added in duplicate to black 96-well plates containing Vero E6 cells (ATCC, CRL-1586) at $2x10^4$ cells per well). Each plate contains virus only (no serum) control wells. The plates were incubated for 24 hours at 37°C and 5% CO_2. After 24 hours, cells were lysed, and luciferase activity measured with the Nano-Glo Luciferase Assay System (Promega). Neutralization activity is expressed as the dilution concentration at which the observed RLU are reduced by 50% or 80% relative to the virus-only controls.

Spike protein-expressing cell antibody binding assay (SECABA)

The cell antibody binding assay was performed based on previously described methods (*68, 69*), modified to use target cells derived by transfection with plasmids expressing the SARS-CoV-2 spike protein with a C-terminus flag tag. We used one plasmid expressing the D614G sequence to generate transfected 293F cells. Cells not transfected with any plasmid (mock transfected) were used as a negative control. After resuspension, washing and counting, $1x10^5$ S protein transfected target cells were dispensed into 96-well V-bottom plates and incubated with six serial dilutions of plasma from RMs infused with either convalescent plasma or two monoclonal antibodies starting at 1:50 dilution. After a 30 minute incubation at 37°C, cells are washed twice

with 250 µL/well of PBS, stained with viability dye (Invitrogen) to exclude nonviable cells from subsequent analysis, washed with Wash Buffer [1% fetal bovine serum (FBS)-PBS; WB], permeabilized with CytoFix/CytoPerm (BD Biosciences), and stained with 1.25 µg/mL anti-Rhesus IgG (H+L)- allophycocyanin (APC; Southern Biotech) and 5 µg/mL anti-flag-fluorescein isothiocyanate (FITC; clone M2; Sigma Aldrich) in the dark for 20 min at RT. After three washes with PermWash (BD Biosciences), the cells were resuspended in 125 µL PBS-1% paraformaldehyde. Samples were acquired within 24 hours using a BD Fortessa cytometer and a High Throughput Sampler (HTS, BD Biosciences), collecting a minimum of 50,000 total events per sample. Data analysis was performed using FlowJo v10 software (TreeStar). Gates were set to include singlet, live and flag+ events. Anti-Rhesus IgG was gated on the secondary antibody alone sample and applied to baseline/immunized RM samples to calculate %IgG+ cells. Results are reported as area under the curve (AUC) of a six dilution half-log (1:50 to 1:10,000) series calculated using the non-linear trapezoidal rule, and mock and baseline were subtracted.

Antibody-dependent natural killer (NK) cell degranulation assay

Cell-surface expression of CD107a was used as a marker for NK cell degranulation, a prerequisite process for ADCC (*70, 71*), performed as previously described (*72*). Briefly, target cells were 293T cells 2-days post transfection with a plasmid expressing SARS-CoV-2 S protein of the D614G or B.1.617.2 variants. Serum samples were tested at 1:100, 1:500, and 1:1,000 dilutions. Samples from a SARS-CoV-2 infected (PC020v1) and a non-infected (SORF Neg) human donor were tested at the same dilutions as positive and negative controls, respectively. NK cells were purified from peripheral blood of a healthy human volunteer by negative selection (Miltenyi Biotech) and were incubated with target cells at a 1:1 ratio in the presence of diluted serum, Brefeldin A (1 µL/mL, BD Biosciences), monensin (GolgiStop, 4 µL/6mL, BD Biosciences), and CD107a-FITC (0.625 µg/mL; BD Biosciences, clone H4A3) in 96-well flat bottom plates for 6 hours at 37ºC and 5% CO_2. NK cells were then recovered and stained for viability prior to staining with antibodies specific for NK cells and cytotoxic function as previously described (*72*). Flow cytometry data analysis was performed using FlowJo v10 (BD Biosciences) and data are reported as the percentage (%) of $CD107a^+$ live NK cells (gating strategy: singlets, lymphocytes, aqua blue-, $CD56^+$ or $CD16^+$, $CD107a^+$). Final data were reported as specific activity for each dilution, determined by subtraction of non-specific activity observed in assays performed with mock-infected cells and in the absence of antibodies and presented as AUC calculated using the non-linear trapezoidal rule.

T Cell Responses

Cryopreserved peripheral blood mononuclear cell (PBMC) or lymph node cell suspensions were thawed, washed and cultured in RPMI-1640 (10^6/mL) with 2 µg/mL overlapping peptides for the S proteins of SARS-CoV-2 wildtype (WT) / Wuhan strain, B.1.617.2, or B.1.1.529, or for SARS-CoV-2 nucleocapsid (N) protein (JPT peptides) or dimethyl sulfoxide vehicle together with co-stimulatory antibodies against CD28 and CD49d (BD Biosciences) (*28*). Positive control samples were stimulated with a cell stimulation cocktail (eBiosciences). Cells were surface stained as outlined **in table S11** and then permeabilized with CytoFix/CytoPerm (BD Biosciences) per manufacturer's recommendations and stained with intracellular antibodies as recommended **(table S11)** and previously described (*28*). Data were collected using an LSRFortessa and BD FACSDiva v8.0 and analyzed with FlowJo software v10 (TreeStar). The gating strategy is illustrated in **fig. S19**. The percentage of cytokine positive $CD4^+$ or $CD8^+$ T cell responses are reported after Boolean gating and subtraction of background.

SARS-CoV-2 RNA analysis

Viral samples in RNALater were inactivated 0.1% Triton X-100 (proteomics grade, VWR) diluted in 1X phosphate-buffered saline (PBS, Life Technologies) at 37°C and with moderate shaking at 500 rpm for 30 minutes. Inactivated samples were then processed using the MagNA Pure 24 Total NA Isolation Kit (Roche Applied Science) or Direct-zol RNA Miniprep Kit (Zymo Research). RNA was eluted using DNAse/RNase-free water. For each processing batch, positive and negative controls were used. Relative viral genome copy number was ascertained by real-time quantitative polymerase chain reaction (qPCR) using primers (*N1, N3, RNP*) and procedures established by the CDC (*73*); forward and reverse primer sequences for *orf1a,b* amplification were 5'-CCCTGTGGGTTTTACACTTAA-3' and 5'-ACGATTGTGCATCAGCTGA-3', respectively. The eluted RNA was subjected to reverse transcription for viral load analysis (*74*). cDNA was used for qPCR containing primers and SYBR green as the method of detection on a Thermo Fisher QuantStudio 7 Pro and crossing point (CP) values determined by an automated threshold method.

Lung pathology analysis

The lungs were harvested, and the trachea cannulated with an 18-gauge blunt needle. Neutral buffered 10% formalin at 30 cm fluid pressure was slowly infused into the lungs. Once fully inflated the lungs were placed into formalin and fixed for 72 hours. Then each lobe was separated and sliced from the hilus to the periphery in slabs approximately 5 mm thick. Each slice was placed in

a cassette (or multiple cassettes if the slices were large), recording its place in the stack, and held in 70% ethanol until routine processing. After processing and paraffin embedding, 4 of the lobes were selected for histology (both caudal lobes, right cranial lobe and left middle lobe). Every second slab from these lobes was sectioned at 5 µm for hematoxylin and eosin (H&E) staining. Depending on the size of the lung lobe, between 20 and 24 slides per animal were blindly and independently evaluated by two pathologists. Each slide was given a single score from 0 to 4 which encompassed the extent and severity of the interstitial and alveolar inflammation, such as “interstitial pneumonia”, as described in **tables S7** and **S8**.

Supplemental Figures

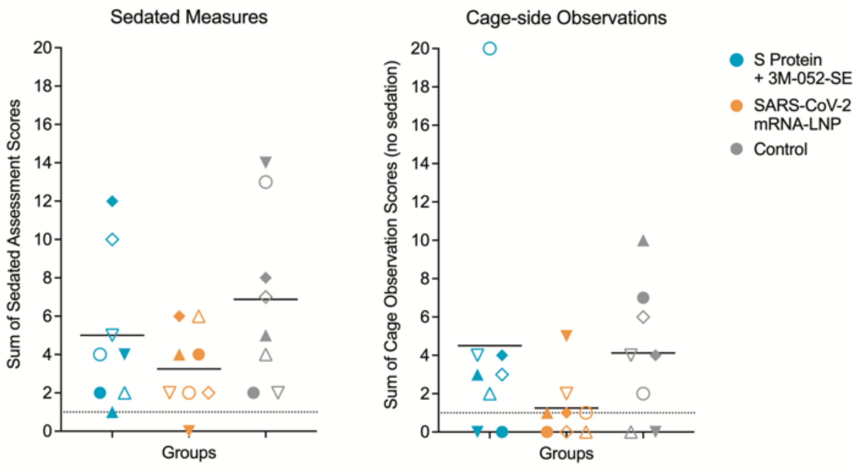

Fig S1. Clinical monitoring scores. Post-SARS-CoV-2 challenge, RMs were monitored daily for clinical signs of disease. Specific measurements were taken when the animals were sedated (such as temperature, heart rate, respiratory rate) and others (such as coughing and responsiveness) while observing the animals in their cage (see Materials and Methods). Each measurement or observation was scored and the sum of all scores over the 7-day post-challenge follow-up period was calculated. The left and right graphs depict the Clinical Monitoring Scores of measures during sedation and the Cage Observation Scores, respectively, for RMs in each group. Each symbol represents an individual RM (see table S1) with n=8 RMs per group; horizontal lines indicate the group median value.

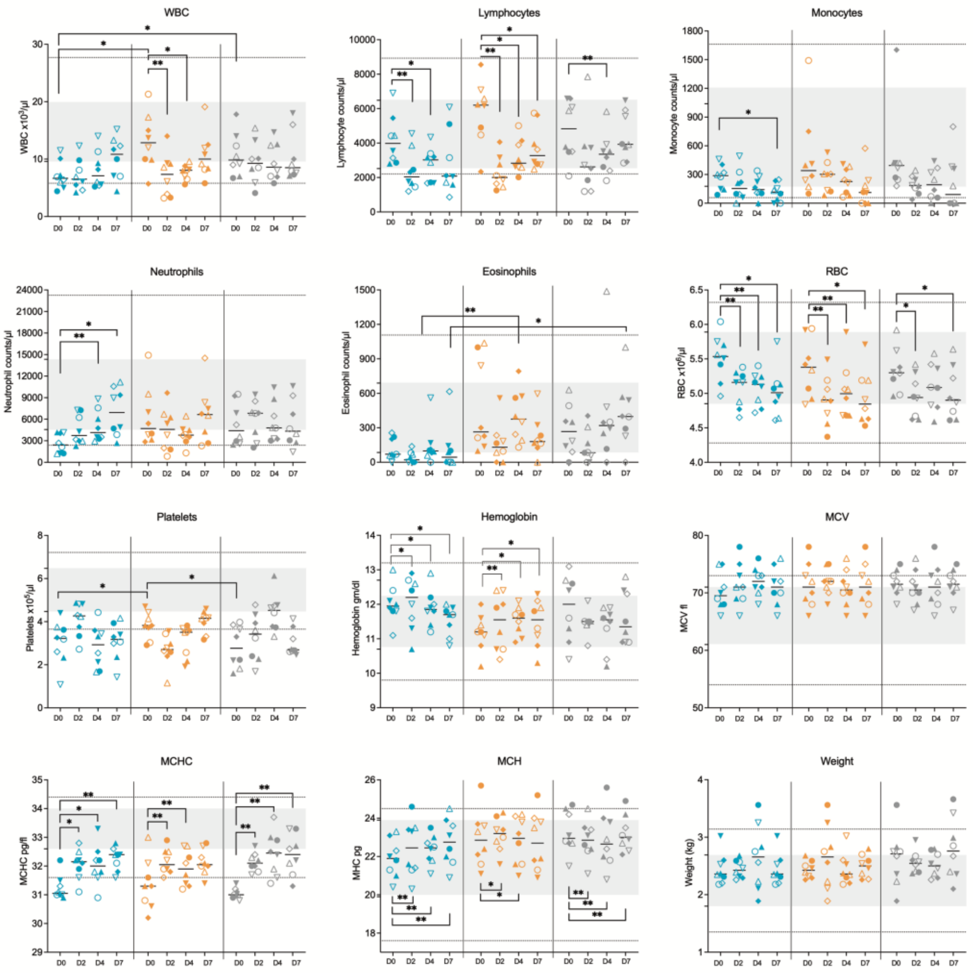

Fig. S2. Complete blood count (CBC) data and other clinical measurements. Peripheral blood differential blood cell counts were obtained on days (D) 0, 2, 4, and 7 post challenge. Data are presented for absolute numbers of white blood cells (WBC), lymphocytes, monocytes, neutrophils, eosinophils, and red blood cells (RBC). Additional measurements include hemoglobin, mean corpuscular volume (MCV), mean corpuscular hemoglobin concentration (MCHC), mean corpuscular hemoglobin (MCH), platelet numbers, and weight. Data in each graph are ordered by group and time. Light gray shaded areas indicate the mean range±1 standard deviation (SD) and upper and lower dashed lines represent minimum and maximum values based on data for 1-year old outdoor RM housed at the UC Davis CNPRC. Each symbol represents an individual RM (see table S1) of the Protein (blue symbols) group, mRNA group (orange symbols), or control RMs (gray symbols); horizontal lines indicate the group median value. Differences within a group between two timepoints were determined by Wilcoxon rank test and differences between RMs of different groups at the same timepoint by Mann-Whitney test, with *p<0.05 and **p<0.01.

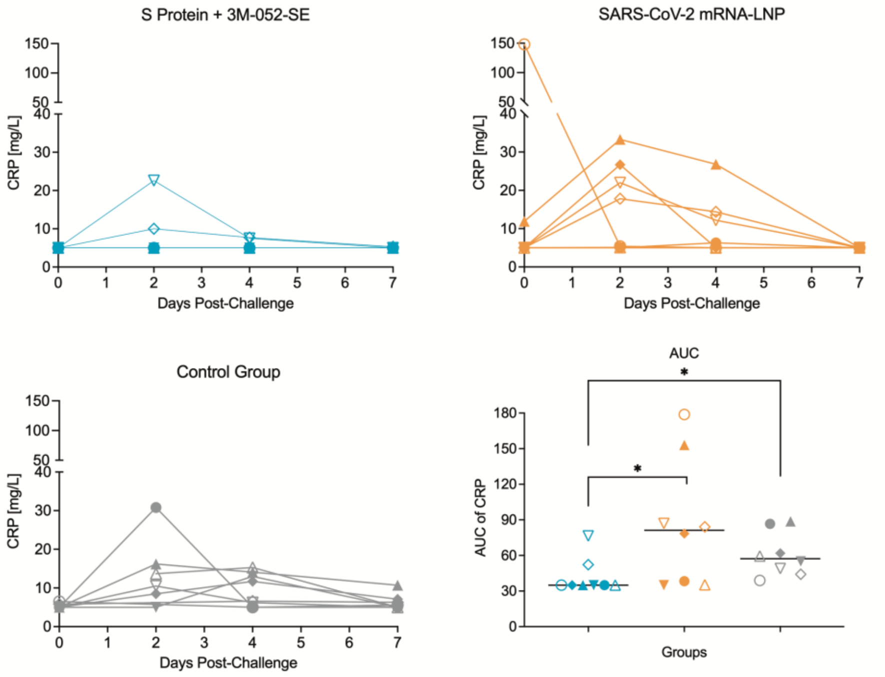

Fig. S3. Serum C-reactive protein (CRP) concentrations after challenge. CRP serum concentrations were measured on days 0, 2, 4 and 7 and are shown for each vaccinated and control RM over time. CRP concentrations [mg/L] for RMs of the same group are plotted in the same graph with individual RMs being identified by their specific symbol and color as described in table S1. The limit of detection was 5 mg/l. Differences in CPR concentrations over time were illustrated by graphing the area under the curve (AUC) of CRP serum concentrations in the bottom right graph. Horizontal lines indicate the group median value. Differences between groups were determined by Mann-Whitney test, with *p<0.05.

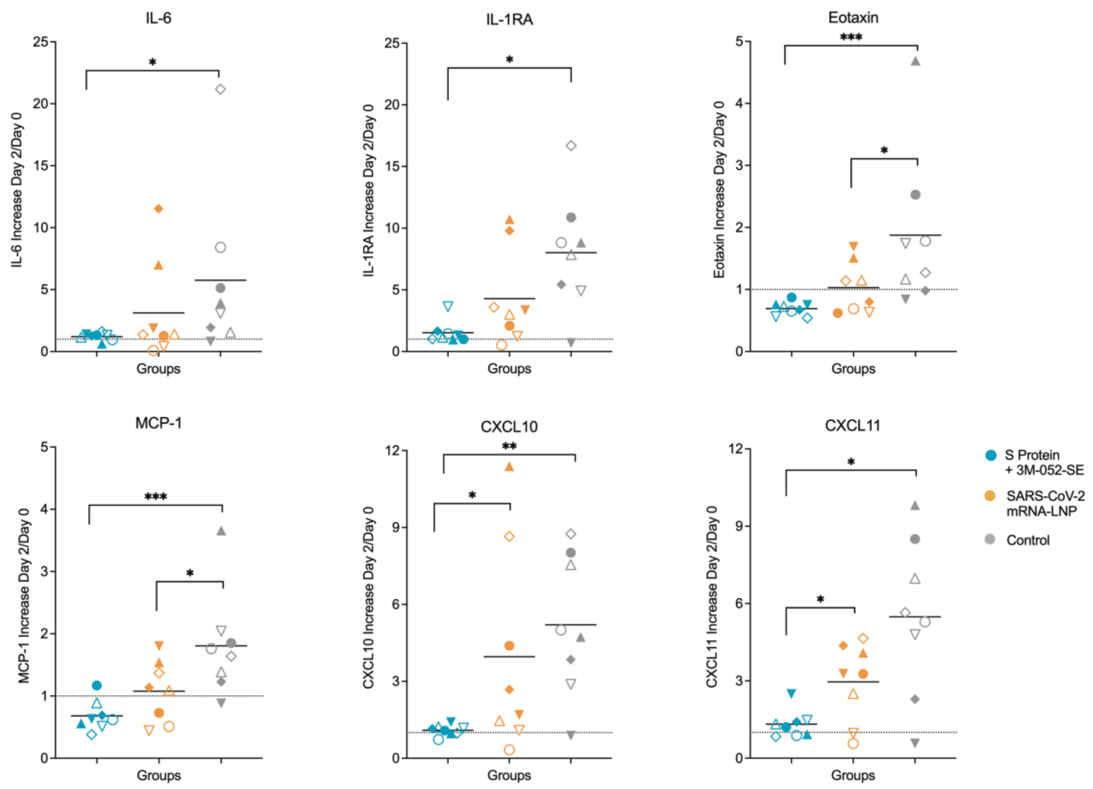

Fig. S4. Increase in plasma cytokines post SARS-CoV-2 challenge. Plasma samples were tested for changes in the concentrations of several cytokines and chemokines. Results represent the increase in cytokine concentration on day 2 compared to day 0 for interleukin (IL)-6, IL-1RA, eotaxin, monocyte chemoattractant protein-1 (MCP-1), CXCL10, and CXCL11. The dashed horizontal lines indicate the lower limit of detection for each of the immune markers. Each symbol represents an individual RM with n=8 RMs per group (see table S1) and horizontal lines indicate the group median value. Differences between two groups were determined by Mann-Whitney test with *p<0.05, **p<0.01, and ***p<0.001.

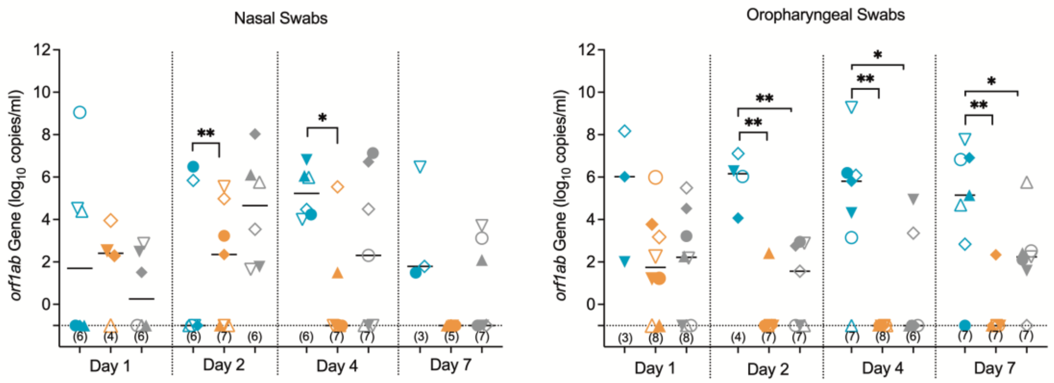

Fig. S5. Genomic SARS-CoV-2 RNA concentrations in mucosal secretions. Genomic viral RNA concentrations of the *orf1a,b* gene were measured by real-time (RT)-PCR and are reported as $\log_{10}$ copies/mL. The left and right graphs show *orf1a,b* copies/mL in nasal or pharyngeal secretions, respectively, on days 1, 2, 4, and 7 after challenge with the B.1.617.2 virus. Each symbol represents an individual RM (table S1) and horizontal lines indicate the group median value. The number of RMs in each group with sufficient sample size to measure *orf1a,b* expression is listed in parentheses above the x-axis for each of the testing days. The dashed horizontal lines indicate the lower limit of detection. Blue, orange, and gray symbols represent RMs of the Protein, mRNA, or control group, respectively. Differences in RNA concentrations between two groups were assessed by Mann-Whitney test with *$p<0.05$ and **$p<0.01$.

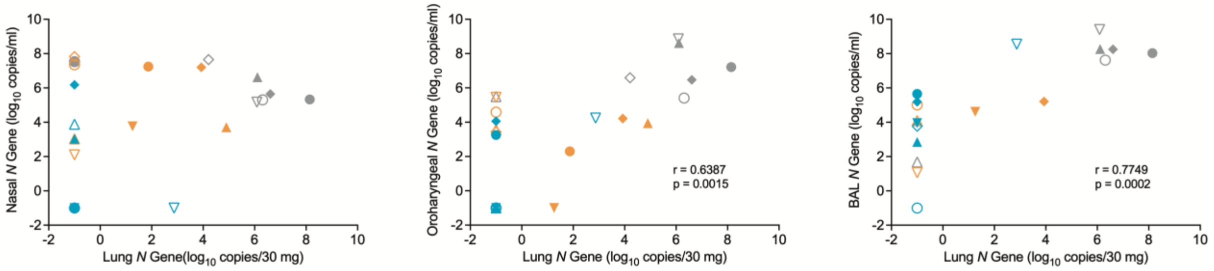

Fig. S6. Correlations between viral RNA concentrations in the upper and lower respiratory tract. Lung viral RNA concentrations (n=8 per group) were plotted against day 4 viral RNA in nasal (left graph) swabs (n=8 per group), oropharyngeal (middle graph) swabs (n=8 per group), or in BAL (right graph; Protein group: n=7, mRNA group: n=5, or control group: n=7 RMs). Correlations were determined by Spearman rank test. Lung SARS-CoV-2 sgRNA concentrations were correlated with day 4 *N* gene RNA concentrations in oropharyngeal secretions and BAL but not with virus replication in nasal secretions. Individual RMs in their respective groups are identified by their specific symbol and color as described in table S1.

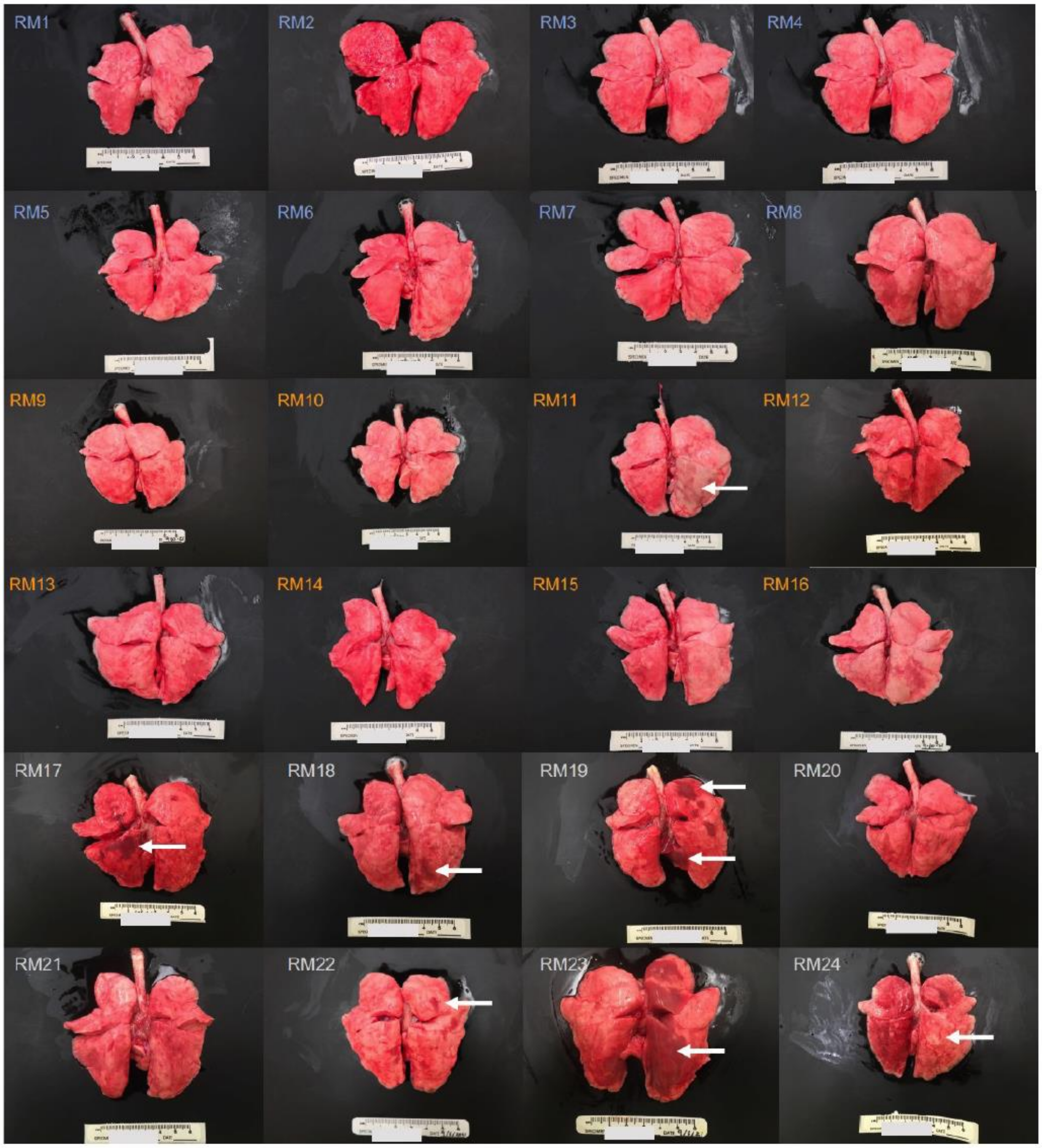

Fig. S7. Lung images. Lung images were taken immediately after perfusion and lung harvest. Images are ordered by experimental group and RM numbers are listed in the top left corner of each image. RMs of the Protein, mRNA, or control group are indicated by blue, orange, or gray designations, respectively. Each image contains a size bar. White arrows point to visible lesions.

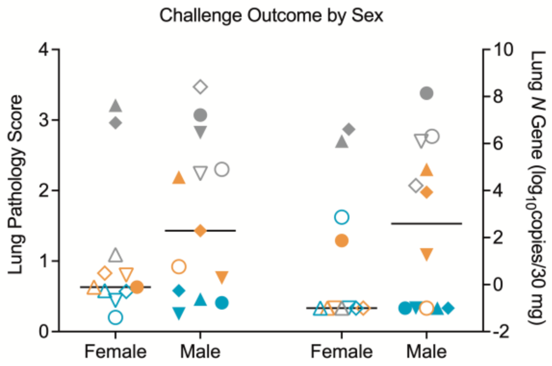

Fig. S8. Challenge outcome in female versus male rhesus macaques. Pathology scores on the left y-axis and lung *N* gene RNA concentrations on the right y-axis were graphed for female versus male RMs independent of their vaccine status. RMs vaccinated with the Protein vaccine (n=8), the mRNA vaccine (n=8), or control RMs (n=8) are represented by blue, orange, or gray symbols, respectively, as noted in table S1. Although median (horizontal lines) values are higher for male RMs, these differences are not statistically significant (Mann-Whitney test, p>0.05).

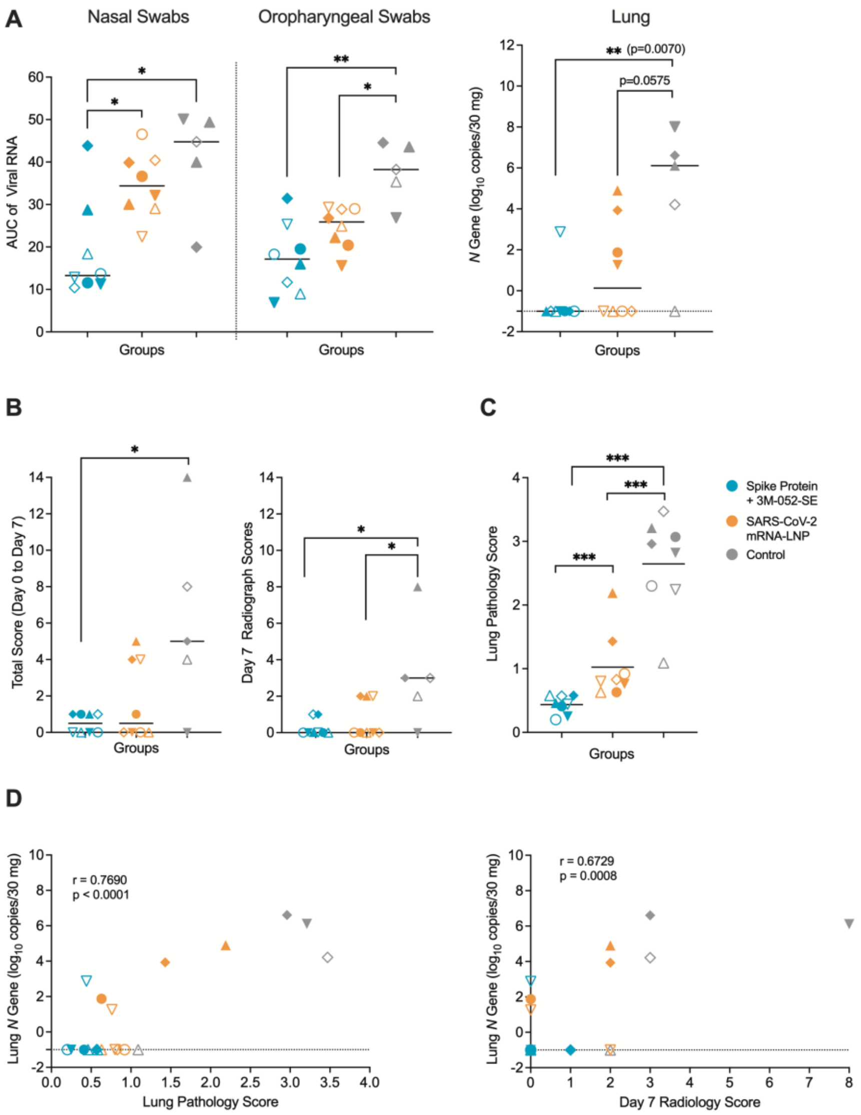

Fig. S9. Sensitivity analysis of challenge outcome. We performed a sensitivity analysis excluding the three control RMs (#17, #18, #21) that were housed outdoors until one month prior to challenge to test whether these altered housing conditions, and therefore potentially altered microbiota, impacted challenge outcome. Data are shown for **(A)** SARS-CoV-2 virus replication measured as AUC in nasal and oropharyngeal sections and in the lung, **(B)** lung radiograph and **(C)** lung pathology scores, and **(D)** for correlations between the lung pathology scores with lung virus replication and lung radiograph scores on day 7 post challenge. The dashed horizontal lines indicate the lower limit of detection. All graphs include results for n=8 Protein and n=8 mRNA vaccinated RMs compared to n=5 control RMs. Differences between two groups were determined by Mann-Whitney test with *p<0.05, **p<0.01, and ***p<0.001. Correlations were assessed by Spearman rank test.

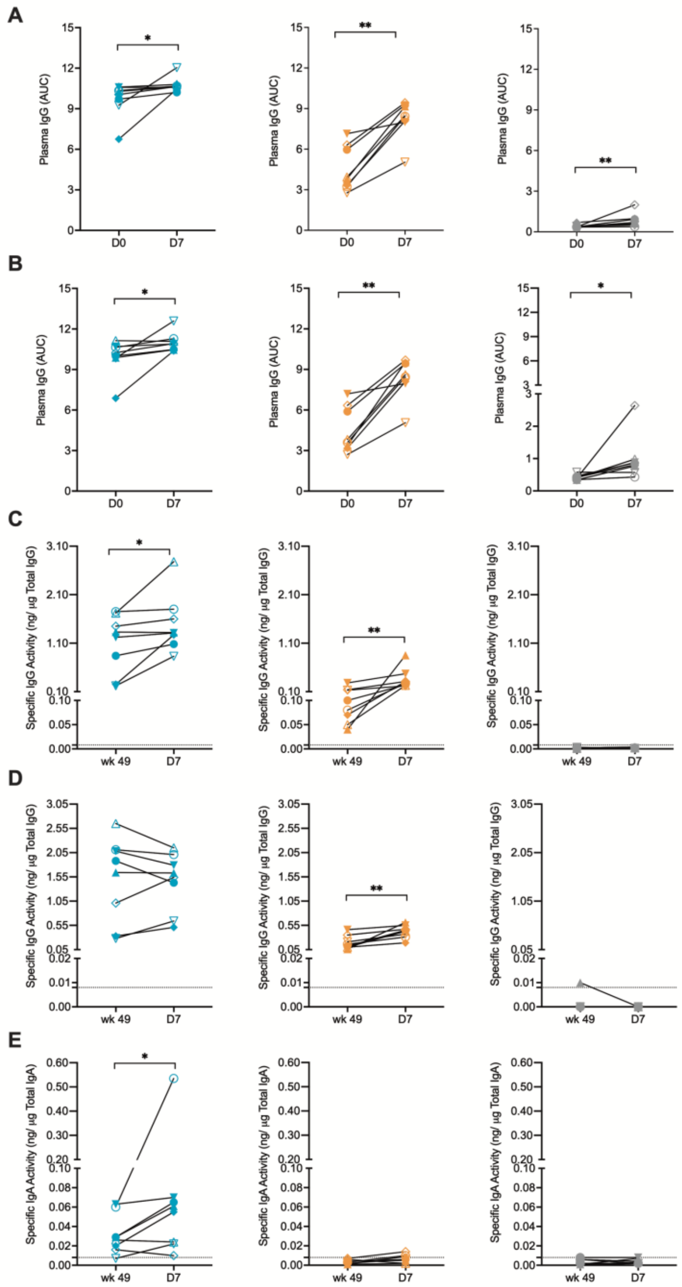

Fig. S10. Post-challenge antibody responses. The day 7 post-challenge antibody responses for each RM are graphed and paired with the day 0 response of the same animal. We assessed plasma **(A)** D614G and **(B)** B.1.617.2 IgG responses, **(C)** nasal and **(D)** salivary B.1.617.2 receptor-binding domain (RBD)-specific IgG activity, and **(E)** B.1.617.2 RBD-specific IgA activity in nasal secretions. The dashed horizontal lines indicate the cut-off for significance (mean + 3SD of negative controls). Differences between the two timepoints were determined by Wilcoxon rank test with *p<0.05, and **p<0.01. Data for n=8 RMs per group and timepoint are shown in each of the graphs.

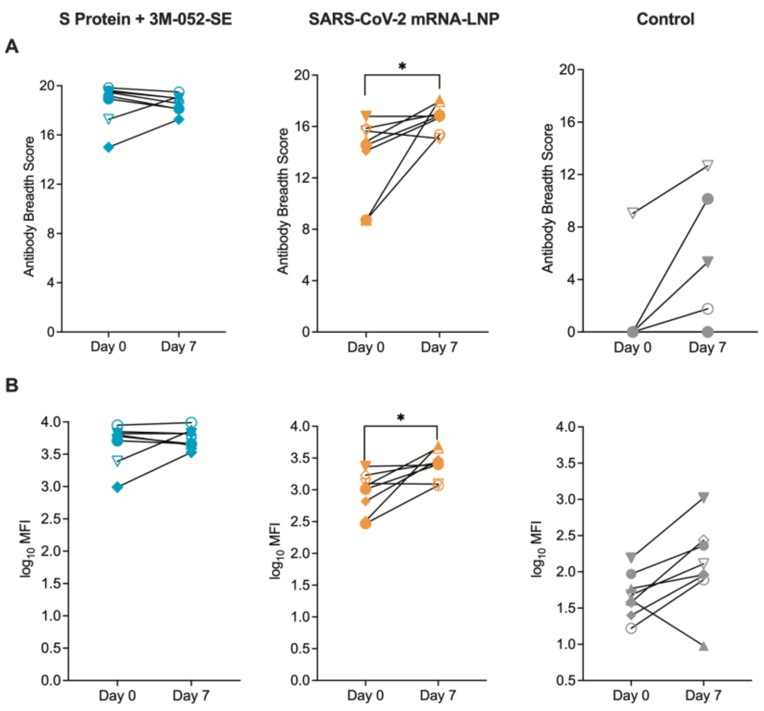

Fig. S11. Plasma antibody breadth post-challenge. (A) Plasma IgG breadth scores (see Materials and Methods) of individual RMs in the two vaccine groups and in control RMs are shown on the day of challenge (day 0) and on day 7 after challenge. Note that plasma antibodies in the single RM of the control group with a breadth score >8 on day 0 did not bind to SARS-CoV-2 nucleoprotein (see Fig. 3; binding to S proteins of variants of concern was confirmed in a repeat sample. **(B)** Median antibody binding was measured as $\log_{10}$ median fluorescence intensity (MFI) to the B.1.617.2 S protein on day 0 and day 7 after challenge. Individual RMs of the two vaccine groups are presented by their respective symbol listed in table S1. Differences between the two timepoints were determined by Wilcoxon rank test with $^*p<0.05$. Data for n=8 RMs per group and timepoint are shown in each of the graphs.

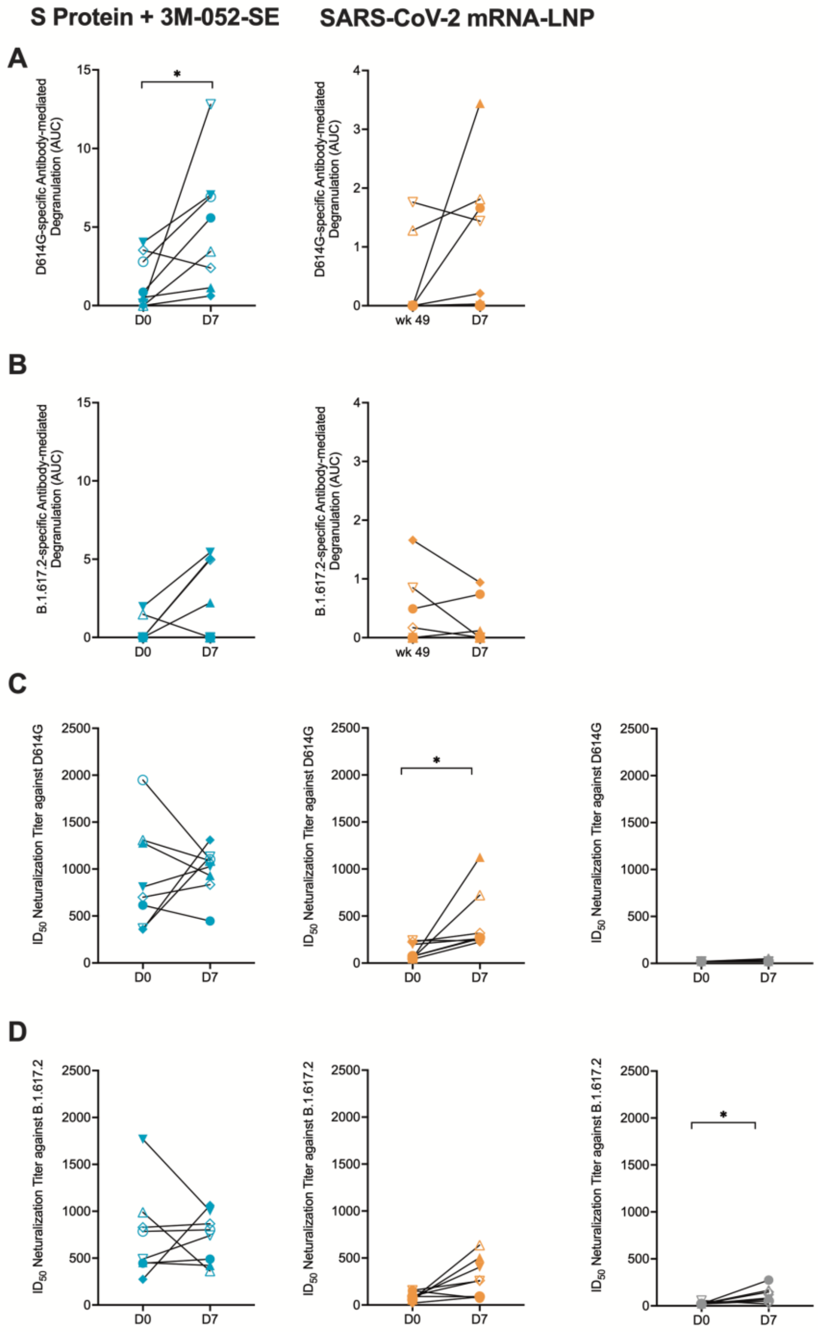

Fig. S12. Post-challenge antibody function. (A and B) The ability of plasma D614G-specific **(A)** or B.1.617.2-specific **(B)** antibodies to induce degranulation of natural killer cells on day 7 compared to day 0 is shown for RMs of the Protein+3M-052-SE or the mRNA-LNP vaccine group. **(C and D)** ID_{50} nAb titers specific for the D614G **(C)** or the B.1.617.2 **(D)** S protein on day 7 compared to day 0 for both vaccine groups (n=8 per group) and control (n=8) RMs. Individual RMs are presented by their respective symbol listed in table S1. Differences between the two timepoints were determined by Wilcoxon rank test with $*p<0.05$.

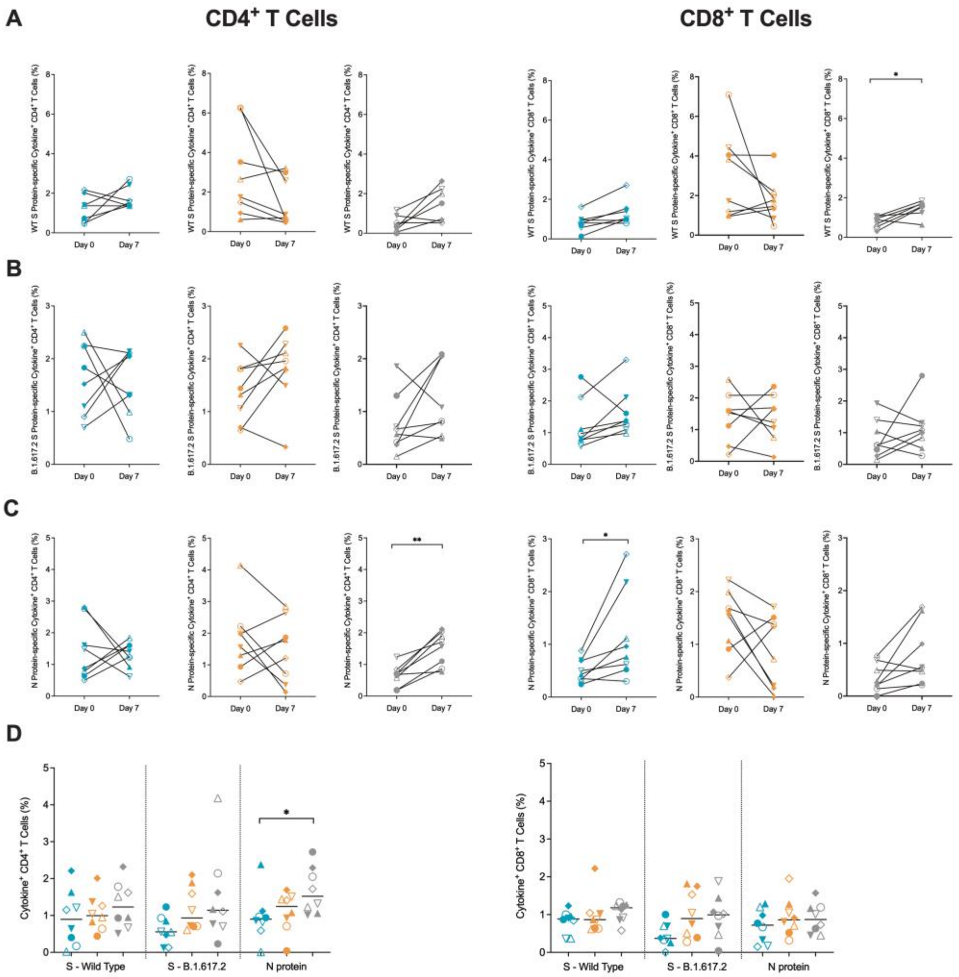

Fig. S13. Post-challenge T cell responses in blood and mediastinal lymph nodes. (A and B) Peripheral blood CD4+ and CD8+ T cell responses to the WT **(A)** or B.1.617.2 **(B)** S protein on day 7 post-challenge compared to day 0. Note that on day 7, due to limited PBMC cell numbers, only n=7 RMs of the Protein and Control groups could be tested for WT S protein-specific CD4+ and CD8+ T cell responses. B.1.617.2 S protein-specific T cell responses were tested for n=8 per group on day 0 and day 7. **(C)** Peripheral blood CD4+ and CD8+ T cell responses to the N protein on day 7 post-challenge compared to day 0; n=8 RMs per group and time point. **(D)** CD4+ and CD8+ T cell responses were measured to the WT or B.1.617.2 S protein, or the N protein on day 7 post-challenge in bronchial lymph nodes, with n=8 RMs per group and time point. Reported are the sum of all single, double, or triple cytokine positive CD4+ or CD8+ T cells as determined by Boolean gating. Horizontal bars indicate medians, each symbol represents an individual RM as outlined in table S1. Differences between RMs of the same group at different timepoints were determined by Wilcoxon rank test with *p<0.05 and **p<0.01. Differences in T cell responses between two groups were determined by Mann-Whitney test with *p<0.05.

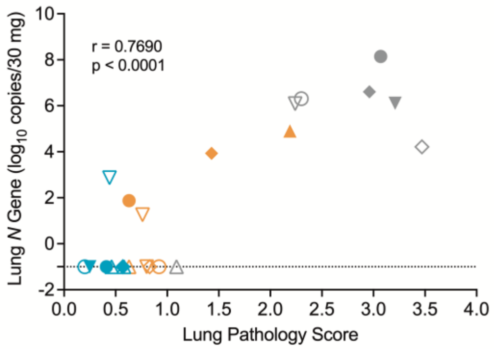

Fig. S14. Correlation between lung inflammation and virus replication. Lung virus replication ($\log_{10}$ copies/30 mg) was positively correlated with lung pathology scores as determined by Spearman rank test. The limit of detection of the viral qRT-PCR assay was 5 copies/30 mg tissue (dashed horizontal line). Each symbol represents an individual RM as outlined in table S1; n=8 RMs per group.

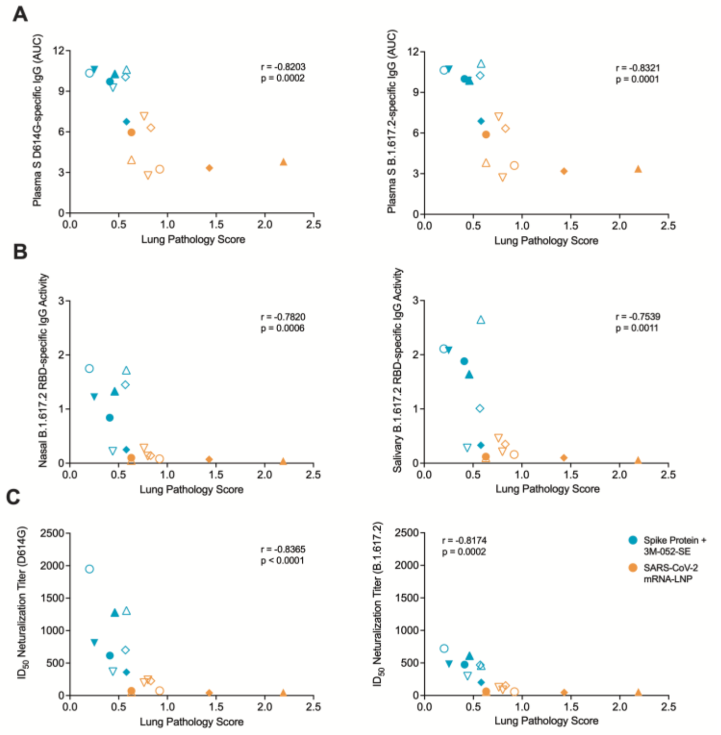

Fig. S15. Correlation between lung pathology and pre-challenge SARS-CoV-2-specific antibody responses at the time of challenge. Lung pathology scores were plotted against day of challenge plasma D614G-specific IgG and B.1.617.2-specific IgG **(A)**, nasal and salivary B.1.617.2 RBD-specific IgG activity **(B)**, and against ID_{50} nAb titers specific for the D614G or the B.1.617.2 S protein **(C)**. Correlations were tested by Spearman rank test. Individual RMs of the two vaccine groups (n=8 RMs per group) are presented by their respective symbol listed in table S1.

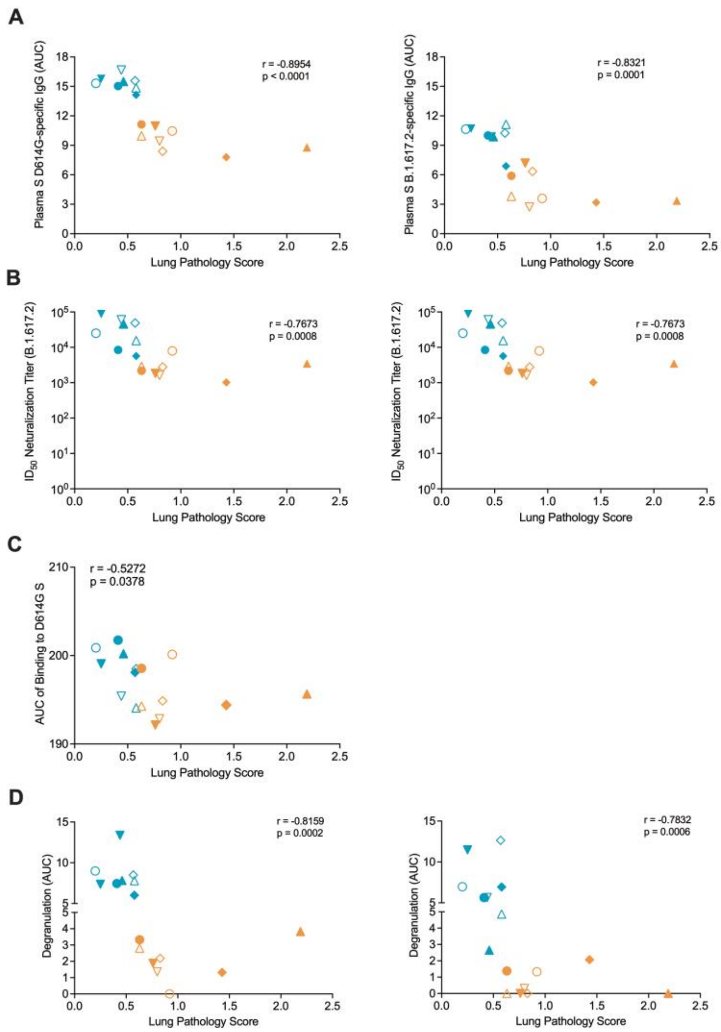

Fig. S16. Peak antibody responses are indicative of lung pathology. Peak antibody responses at week 6, two weeks after the last immunization, including **(A)** plasma D614G- and B.1.617.2-specific binding antibody responses, **(B)** D614G- and B.1.617.2-specific neutralizing ID_{50} titers, **(C)** binding of plasma IgG to D614G-transfected cells, and **(D)** degranulation function of D614G- and B.1.617.2-specific IgG were inversely correlated with lung pathology. Correlations were assessed by Spearman rank test. Individual RMs of the two vaccine groups (n=8 per group) are presented by their respective symbol listed in table S1.

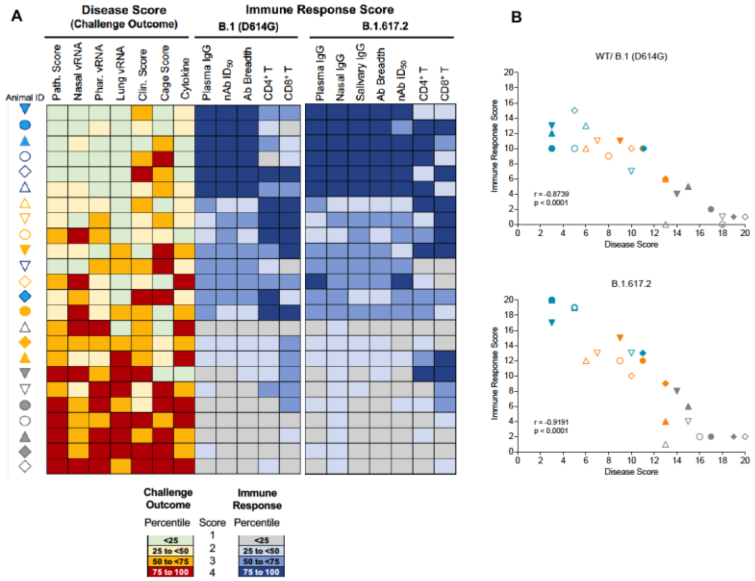

Fig. S17. Correlation matrix of immune response parameters at the time of challenge with challenge outcome parameters. (A) Shown are parameters used to assess D614G or WT-SARS-CoV-2-specific immune responses on the day of challenge, including plasma binding IgG, nAb ID_{50} titers, and peripheral blood $CD4^+$ and $CD8^+$ T cell responses. Day of challenge immune responses specific for B.1.617.2 included plasma binding IgG, nAb ID_{50} titers, nasal and salivary IgG specific activity, and peripheral blood $CD4^+$ and $CD8^+$ T cell responses. Challenge outcome parameters included the lung pathology score, lung viral RNA, nasal and pharyngeal viral RNA reported as AUC over the 7-day post-challenge period, the sedated and cage-side clinical observation scores, and the day 2 increase in systemic plasma cytokines. Data for each parameter across the 3 groups were tabulated and then the 25th, 50th, and 75th percentiles were calculated. The actual data for each RM were then color-coded based on their percentile as shown in the legend. In addition, data were scored based on their percentile with data below the 25th percentile being assigned a score of “1”, between the 25th and 50th percentile a score of “2”, between the 50th and 75th percentile a score of “3”, and a score of “4” for data >75Th percentile. The data were entered into a matrix and ordered from lowest (no or mild) to highest (severe) challenge outcome score. A separate matrix was assembled for WT/D614G-specific immune responses and B.1.617.2-specific immune responses. Each column represents an immune or challenge outcome parameter and each row represents an individual RM. **(B)** Correlation of the sum of immune response scores with the sum of challenge outcome scores, graphed separately for WT/D614G- and B.1.617.2-specific immune responses. Spearman rank test was applied to determine the correlation strength. RMs are identifiable by their respective symbols as described in table S1.

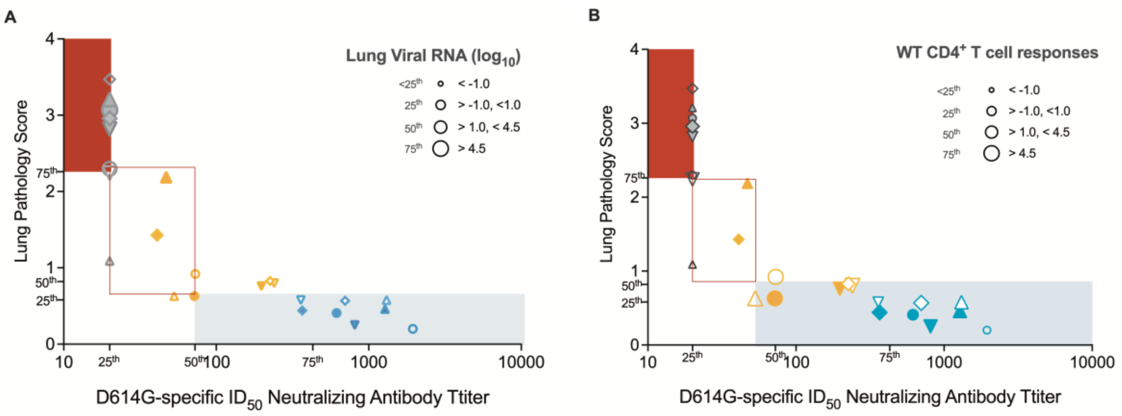

Fig. S18. Immune correlates of protection. ID_{50} titers of D614G-specific nAbs at the time of challenge are plotted against lung pathology scores. The symbol size for each RM is based on **(A)** the degree of virus replication in the lung, or **(B)** $CD4^+$ T cell responses in the same RM. RMs in the Protein or mRNA vaccine groups are indicated by blue and orange circles, respectively, and control RMs by gray circles as outlined in table S1. Note that RMs with ID_{50} titers >50 have pathology scores <1. Vertical ticks indicate the 25th, 50th and 75th percentile of all ID_{50} titers across all groups and horizontal ticks indicate the 25th, 50th and 75th percentile of lung pathology scores (table S8). Most control RMs have ID_{50} nAb titers at or below the 25th percentile and pathology scores above the 75th percentile, whereas the majority of Protein+3M-052 vaccinated RMs have ID_{50} nAb titers above the 75th percentile and pathology scores at or below the 25th percentile. RMs of the mRNA-LNP group have intermediate (between the 25th and 75th percentile) ID_{50} nAb titers and pathology scores. Red-filled, red-outlined, and light gray shaded areas indicate severe, intermediate, and no or mild lung pathology, respectively.

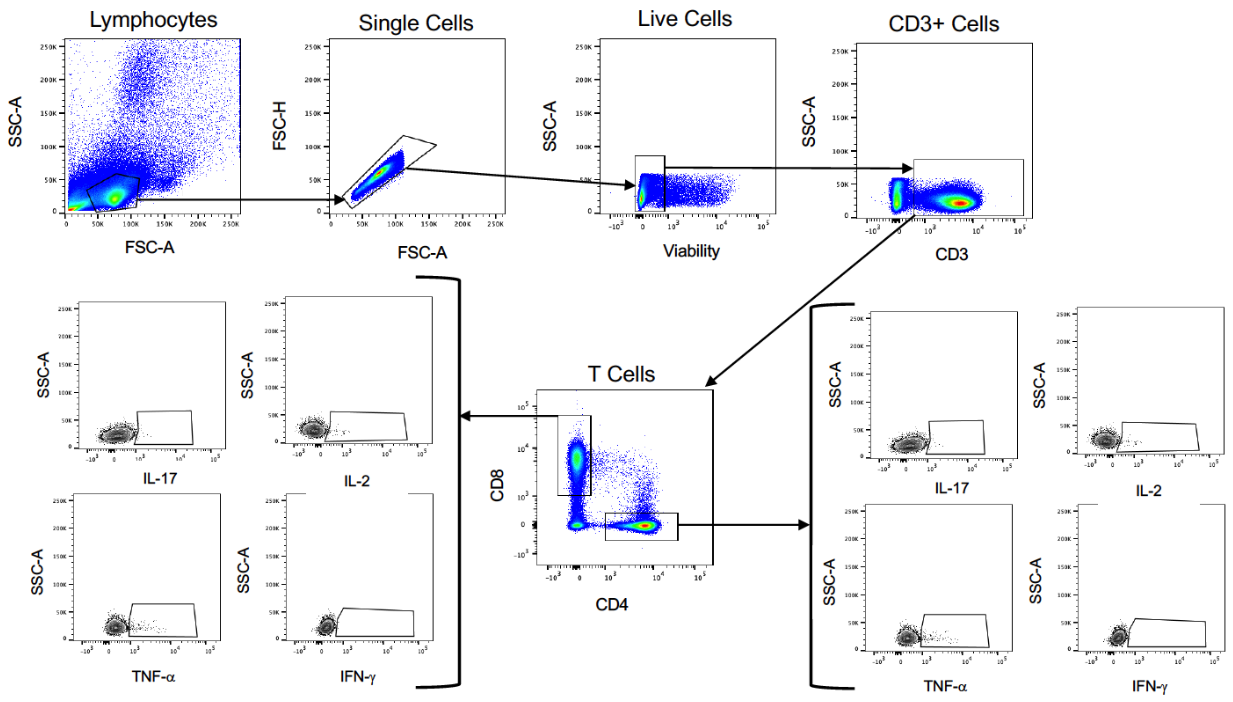

Fig. S19. Gating strategy for antigen-specific T cell responses. In vitro antigen-stimulated samples were acquired on the LSRFortessa and analyzed using FlowJo, v. 10. A lymphocyte gate was set to gate for single, live cells. Viable CD3^{+} T cells were then gated into CD4^{+} and CD8^{+} T cell populations that were then probed for IL-2, interferon (IFN)-γ, IL-17, or tumor necrosis factor (TNF)-α positive cells. Boolean gating was applied to determine single, double, triple, or quadruple-positive CD4^{+} and CD8^{+} T cells. FSC, forward scatter; SSC, side scatter; A, area; H, height.

Table S1. Experimental groups and animals.

Vaccine Group (color coding)	Animal ID	Sex	Symbol
**S Protein+3M-052-SE** (blue)	RM1	male	filled circle
	RM2	female	open circle
	RM3	male	filled triangle
	RM4	female	open triangle
	RM5	female	open inverted triangle
	RM6	male	filled inverted triangle
	RM7	female	open diamond
	RM8	male	filled diamond
**SARS-CoV-2 mRNA-LNP** (orange)	RM9	female	open circle
	RM10	male	filled circle
	RM11	male	filled triangle
	RM12	female	open triangle
	RM13	male	filled inverted triangle
	RM14	female	open inverted triangle
	RM15	male	filled diamond
	RM16	female	open diamond
**Control (no vaccine)** (gray)	RM17	male	filled circle
	RM18	male	open circle
	RM19	female	filled triangle
	RM20	female	open triangle
	RM21	male	open inverted triangle
	RM22	male	filled inverted triangle
	RM23	male	open diamond
	RM24	female	filled diamond

Table S2. Polyfunctional T Cell Responses in Peripheral Blood and Tissues.

Parameter	CD4+ T Cells Average Response (%)			CD8+ T Cells Average Response (%)		
	Single	Double	Triple[a,b]	Single	Double	Triple[a,b]
**PBMC - Day of Challenge (D0)**						
S Protein+3M-052-SE						
D614G	96.38	3.60	0.02	97.58	2.25	0.18
B.1.617.2	97.70	2.11	0.19	97.73	2.02	0.26
B.1.1.529	98.14	1.76	0.10	96.57	3.18	0.26
N-protein	99.02	0.98	0.00	97.49	2.30	0.20
SARS-CoV-2mRNA-LNP						
D614G	96.42	3.52	0.06	95.43	3.99	0.58
B.1.617.2	96.54	3.46	0.00	94.93	5.04	0.03
B.1.1.529	96.30	3.70	0.00	93.42	6.06	0.52
N-protein	97.01	2.97	0.01	95.10	4.45	0.45
Control						
D614G	74.80	23.38	1.82	93.24	2.79	3.97
B.1.617.2	98.02	1.93	0.05	95.71	3.31	0.97
B.1.1.529	97.89	2.11	0.00	82.92	16.30	0.78
N-protein	99.93	0.00	0.10	95.02	3.97	1.01
**PBMC - Day 7**						
S Protein+3M-052-SE						
D614G	89.43	8.02	2.55	93.45	6.18	0.36
B.1.617.2	84.21	10.73	5.07	95.72	3.65	0.63
B.1.1.529	89.01	7.41	3.58	94.80	5.20	0.00
N-protein	96.79	2.82	0.39	94.44	4.85	0.72
SARS-CoV-2mRNA-LNP						
D614G	94.54	4.80	0.65	95.12	4.34	0.54
B.1.617.2	96.47	3.06	0.47	95.15	4.05	0.80
B.1.1.529	96.90	2.92	0.18	95.48	3.30	1.22
N-protein	97.14	2.80	0.06	87.81	6.14	6.05
Control						
D614G	92.25	6.10	1.65	94.84	4.03	1.13
B.1.617.2	90.04	7.35	2.61	93.97	4.85	1.18
B.1.1.529	92.81	5.62	1.57	91.17	7.20	1.63
N-protein	93.35	5.27	1.38	93.03	4.65	2.32
**Bronchial Lymph Nodes - Day 7**						
S Protein+3M-052-SE						
D614G	78.72	11.32	9.96	88.53	7.75	3.72
B.1.617.2	86.35	12.08	1.49	85.73	1.43	0.35
B.1.1.529	79.85	16.26	3.89	95.81	4.13	0.06
N-protein	84.08	2.96	0.56	91.45	7.90	0.64
SARS-CoV-2mRNA-LNP						
D614G	88.99	9.02	1.99	97.52	2.13	0.35
B.1.617.2	91.88	6.34	1.78	97.53	1.77	0.79
B.1.1.529	91.13	6.95	1.93	97.16	2.69	0.27
N-protein	86.81	8.73	4.49	98.53	1.81	0.64
Control						
D614G	89.26	9.66	1.08	90.04	9.66	0.39
B.1.617.2	89.37	9.76	0.99	77.02	22.86	0.16
B.1.1.529	92.89	6.49	0.73	86.19	13.73	0.11
N-protein	93.49	6.04	0.47	91.10	8.69	0.31

[a] Single, double, or triple cytokine-positive T cells were determined by Boolean gating (see Materials and Methods) and are reported as percent of total S protein-specific; cytokine-producing CD4+ or CD8+ T cells T cells positive for all 4 cytokines were not detected. [b] Statistically, responses did not differ between the groups.

Table S3. Blood Chemistry Data.

**Analyte**	**Day**	**Group**<br>**S Protein + 3M-052-SE**	**SARS-CoV-2 mRNA LNP**	**Control**
Glucose [mg/dl]	0	58.5 ± 4.4	57.6 ± 7.6	74.3 ± 5.0
	2	68.4 ± 5.0	67.0 ± 12.7	63.4 ± 7.9
	4	64.0 ± 7.7	66.9 ± 26.2	55.3 ± 9.9
	7	72.9 ± 26.0	62.6 ± 21.9	71.3 ± 21.8
BUNa [mg/dl]	0	18.0 ± 2.5	17.9 ± 2.7	16.9 ± 5.3
	2	16.8 ± 3.0	18.3 ± 3.8	15.5 ± 2.7
	4	15.5 ± 2.4	19.4 ± 2.8	13.5 ± 3.5
	7	17.5 ± 2.9	17.5 ± 3.2	13.0 ± 3.2
Creatine [mg/dl]	0	0.5 ± 0.1	0.4 ± 0.1	0.5 ± 0.1
	2	0.5 ± 0.2	0.5 ± 0.1	0.6 ± 0.1
	4	0.6 ± 0.1	0.4 ± 0.1	0.5 ± 0.1
	7	0.5 ± 0.2	0.5 ± 0.2	0.5 ± 0.1
Calcium [mg/dl]	0	9.8 ± 0.5	9.7 ± 0.3	9.6 ± 0.6
	2	9.9 ± 0.4	9.5 ± 0.3	9.4 ± 0.4
	4	10.0 ± 0.4	9.6 ± 0.4	9.4 ± 0.5
	7	10.2 ± 0.4	9.2 ± 1.5	9.5 ± 0.5
Albumin [g/dl]	0	3.0 ± 0.3	2.9 ± 0.1	2.9 ± 0.3
	2	3.0 ± 0.2	2.8 ± 0.1	2.9 ± 0.1
	4	2.9 ± 0.2	2.7 ± 0.2	2.8 ± 0.2
	7	3.0 ± 0.2	2.7 ± 0.5	2.7 ± 0.1
Total Protein [g/dl]	0	5.9 ± 0.3	5.9 ± 0.4	6.0 ± 0.6
	2	6.0 ± 0.2	5.9 ± 0.3	6.1 ± 0.3
	4	6.0 ± 0.2	5.9 ± 0.2	6.1 ± 0.2
	7	6.1 ± 0.1	5.6 ± 0.9	6.0 ± 0.2
ALT[b] [U/l]	0	38.8 ± 10.5	46.6 ± 11.1	38.3 ± 5.9
	2	52.3 ± 13.0	56.0 ± 12.2	47.5 ± 6.5
	4	45.8 ± 11.4	46.0 ± 7.1	46.0 ± 18.4
	7	39.3 ± 13.1	37.1 ± 7.3	34.4 ± 7.9
AST[c] [U/l]	0	51.5 ± 6.0	63.0 ± 14.5	47.9 ± 8.1
	2	65.8 ± 16.4	68.6 ± 27.4	64.3 ± 14.4
	4	45.5 ± 7.6	53.4 ± 12.9	59.3 ± 13.4
	7	49.0 ± 12.3	49.9 ± 19.8	51.3 ± 10.0
ALP[d] [U/l]	0	646.6 ± 128.5	598.4 ± 159.3	502.1 ± 99.9
	2	660.1 ± 143.3	534.6 ± 106.6	578.8 ± 115.9
	4	588.5 ± 138.3	496.6 ± 106.6	536.8 ± 110.4
	7	605.5 ± 136.4	507.0 ± 151.0	512.5 ± 77.3
GGT[e] [U/l]	0	90.4 ± 10.0	68.6 ± 20.9	61.3 ± 7.4
	2	87.5 ± 10.3	64.3 ± 18.5	66.1 ± 18.5
	4	81.6 ± 9.3	60.5 ± 15.6	63.6 ± 17.0
	7	79.5 ± 8.3	57.4 ± 18.0	60.4 ± 17.8

Analyte	Day	S Protein + 3M-052-SE	SARS-CoV-2 mRNA LNP	Control
Amylase [U/l]	0	255.0 ± 33.6	271.1 ± 68.4	265.0 ± 48.4
	2	238.9 ± 58.9	240.1 ± 63.5	246.9 ± 76.3
	4	237.8 ± 57.4	248.0 ± 63.8	255.1 ± 79.1
	7	226.4 ± 63.3	225.9 ± 63.9	261.0 ± 89.1
C-reactive Protein [mg/l]	0	5.0 ± 0.0	23.7 ± 50.3	5.3 ± 0.6
	2	7.8 ± 6.2	15.0 ± 11.5	14.1 ± 9.1
	4	5.7 ± 1.2	10.0 ± 7.7	9.6 ± 4.3
	7	5.0 ± 0.1	5.0 ± 0.0	6.2 ± 2.0

[a] BUN, Blood urea nitrogen
[b] ALT, alanine transaminase
[c] AST, aspartate aminotransferase
[d] ALP, alkaline phosphatase
[e] GGT, gamma-glutamyl transpeptidase

Table S4. Plasma Cytokine Concentrations after SARS-CoV-2 challenge.

Cytokine[a]	Day	Cytokine [pg/mL]		
		S Protein + 3M-052-SE	SARS-CoV-2 mRNA LNP	Control
IL-1β	0	9.1 ± 4.4	12.7 ± 6.6	9.2 ± 5.4
	2	8.8 ± 5.0	10.0 ± 4.3	11.6 ± 1.6
	4	11.7 ± 3.8	13.4 ± 3.4	9.8 ± 4.3
	7	11.2 ± 3.0	11.2 ± 2.6	8.1 ± 4.6
IL-1RA	0	135.3 ± 36.6	188.8 ± 151.2	193.6 ± 89.4
	2	195.1 ± 85.6	621.3 ± 510.5	1,315.0 ± 791.5
	4	120.7 ± 37.1	184.7 ± 143.6	786.4 ± 982.5
	7	121.5 ± 40.6	159.0 ± 62.2	485.8 ± 364.1
IL-2	0	295.0 ± 194.5	226.9 ± 126.0	376.4 ± 347.1
	2	304.0 ± 249.1	276.1 ± 209.1	366.7 ± 229.5
	4	431.6 ± 316.9	283.1 ± 175.4	376.0 ± 200.7
	7	354.9 ± 274.8	240.4 ± 154.0	535.7 ± 206.7
IL-4	0	30.2 ± 16.3	39.0 ± 0.0	39.3 ± 0.9
	2	39.0 ± 0.0	39.0 ± 0.0	35.6 ± 9.7
	4	34.4 ± 12.8	39.0 ± 0.0	37.1 ± 5.4
	7	36.6 ± 6.7	39.0 ± 0.0	32.8 ± 13.2
IL-5	0	3.8 ± 1.9	3.3 ± 1.5	2.2 ± 4.1
	2	3.6 ± 1.9	3.0 ± 1.8	4.4 ± 2.1
	4	3.8 ± 1.6	3.2 ± 1.8	5.2 ± 1.5
	7	2.9 ± 1.5	3.8 ± 1.8	5.4 ± 2.8
IL-6	0	3.1 ± 1.4	5.3 ± 8.5	3.4 ± 1.3
	2	3.4 ± 0.9	8.2 ± 10.6	18.1 ± 24.1
	4	2.5 ± 1.1	3.5 ± 4.9	11.0 ± 11.8
	7	2.8 ± 1.0	3.2 ± 1.7	4.4 ± 2.7
IL-8	0	4.3 ± 2.5	21.2 ± 47.4	31.7 ± 79.8
	2	4.7 ± 2.9	19.5 ± 42.8	57.4 ± 153.5
	4	4.7 ± 3.5	18.9 ± 38.2	30.5 ± 75.3
	7	5.6 ± 2.7	12.2 ± 26.3	60.0 ± 139.0
IL-10	0	9.6 ± 4.7	10.0 ± 4.6	9.7 ± 4.1
	2	9.8 ± 5.4	10.0 ± 4.1	10.8 ± 4.4
	4	10.1 ± 3.9	11.2 ± 3.2	12.2 ± 2.6
	7	10.0 ± 4.2	8.7 ± 4.9	13.4 ± 0.3
IL-12	0	1,281.7 ± 546.2	1,384.2 ± 532.0	1,432.8 ± 745.4
	2	783.2 ± 199.8	876.4 ± 398.3	863.4 ± 517.8
	4	794.2 ± 225.3	967.6 ± 291.2	940.8 ± 420.8
	7	862.7 ± 278.3	1,066.7 ± 395.1	1,013.1 ± 594.3
IL-15	0	115.7 ± 182.2	52.6 ± 49.6	81.2 ± 62.6
	2	97.2 ± 109.1	104.4 ± 55.4	85.1 ± 34.4
	4	64.6 ± 62.9	98.5 ± 76.4	53.6 ± 27.8
	7	45.0 ± 41.6	76.1 ± 68.6	68.9 ± 36.1

Cytokine[a]	Day	Cytokine [pg/mL]		
		S Protein + 3M-052-SE	SARS-CoV-2 mRNA LNP	Control
IL-17A	0	17.5 ± 12.4	29.0 ± 5.3	33.7 ± 20.1
	2	19.1 ± 12.4	27.0 ± 9.6	30.2 ± 14.3
	4	21.6 ± 10.5	29.7 ± 4.5	32.5 ± 6.6
	7	18.7 ± 12.8	23.2 ± 9.5	28.0 ± 14.7
IFN-γ	0	52.9 ± 40.4	50.4 ± 77.0	63.6 ±95.5
	2	54.0 ± 21.2	44.1 ± 30.1	58.7 ± 35.9
	4	47.7 ± 41.6	28.5 ± 14.9	49.7 ± 30.6
	7	38.3 ± 35.0	22.4 ± 13.2	74.9 ± 44.7
CXCL9	0	50.4 ± 14.0	65.5 ± 46.0	80.8 ± 61.7
	2	55.1 ± 15.4	71.5 ± 25.5	99.8 ± 65.3
	4	55.2 ± 14.2	54.5 ± 14.0	98.1 ± 41.0
	7	50.8 ± 15.4	59.9 ± 19.1	103.5 ± 46.4
CXCL10	0	3.8 ± 1.6	4.4 ± 3.5	5.8 ± 4.2
	2	4.0 ± 1.7	9.1 ± 4.9	23.2 ± 11.1
	4	4.0 ± 1.5	3.6 ± 1.8	9.5 ± 5.4
	7	4.4 ± 1.2	3.9 ± 1.0	6.9 ± 3.4
TNF-α	0	19.8 ± 16.0	13.8 ± 3.4	28.4 ± 37.6
	2	17.4 ± 8.2	13.1 ± 8.3	23.1 ± 24.4
	4	21.8 ± 13.5	18.7 ± 11.6	21.9± 17.2
	7	18.3 ± 9.9	21.7 ± 15.9	26.2 ± 27.2
MIP-1α	0	51.1 ± 22.1	36.1 ± 9.6	66.6 ± 77.2
	2	41.5 ± 16.4	34.7 ± 16.1	44.8 ± 42.7
	4	40.8 ± 19.5	33.2 ± 11.1	43.0 ± 31.6
	7	40.4 ± 17.7	41.8 ± 17.7	59.7 ± 48.5
MIP-1	0	64.8 ± 55.1	26.6 ± 11.4	60.6 ± 92.6
	2	52.2 ± 38.1	25.4 ± 11.4	46.3 ± 44.5
	4	54.5 ± 41.6	25.5 ± 14.0	41.2 ± 42.8
	7	57.7 ± 35.5	29.9 ± 16.2	57.6 ± 52.9
RANTES	0	2,808.1 ± 1,107.7	3,274.4 ± 1,242.4	3,754.3 ± 1,476.5
	2	2,628.4 ± 951.4	2,451.1 ± 1,224.2	3,454.2 ± 926.2
	4	2,486.0 ± 975.4	2,461.9 ± 876.5	3,049.7 ± 725.5
	7	2,356.7 ± 1,301.8	2,468.0 ± 1,058.6	3,221.1 ± 868.8
I-TAC	0	115.8 ± 28.1	125.7 ± 121.1	163.4 ± 158.4
	2	148.6 ± 52.4	257.0 ± 130.4	573.4 ± 236.1
	4	100.5 ± 36.8	114.5 ± 61.3	285.6 ± 275.8
	7	84.9 ± 43.4	76.9 ± 26.9	319.2 ± 296.3
MDC	0	2,485.2 ± 1,037.7	2,112.7 ± 1,248.9	1,851.2 ± 1,163.2
	2	2,259.6 ± 814.2	1,794.7 ± 1,096.3	1,791.5 ± 990.7
	4	2,406.4 ± 1,040.1	1,822.9 ± 1,130.4	1,795.4 ± 957.2
	7	2,552.3 ± 1,272.8	2,394.7 ± 1,982.9	2,351.3 ± 1,215.0

Cytokine[a]	Day	Cytokine [pg/mL]		
		**S Protein + 3M-052-SE**	**SARS-CoV-2 mRNA LNP**	**Control**
MCP-1	0	547.8 ± 256.0	440.2 ± 158.6	399.0 ± 102.7
	2	342.7 ± 129.4	439.6 ± 198.0	689.9 ± 268.2
	4	340.0 ± 129.8	352.0 ± 88.3	617.9 ± 201.1
	7	336.0 ± 159.7	321.4 ± 85.4	561.2 ± 256.1
MIF	0	310.8 ± 156.0	323.1 ± 108.7	339.6 ± 126.7
	2	226.8 ± 162.9	177.7 ± 60.2	234.4 ± 71.7
	4	174.3 ± 53.5	211.4 ± 71.1	267.6 ± 125.3
	7	217.6 ± 129.7	230.8 ± 96.9	270.9 ± 220.4
G-CSF	0	92.8 ± 2.8	79.0 ± 12.4	93.9 ± 15.4
	2	86.1 ± 8.2	80.4 ± 10.0	80.4 ± 15.9
	4	83.9 ± 10.0	72.9 ± 39.6	87.9 ± 12.1
	7	83.0 ± 12.8	100.3 ± 39.6	108.4 ± 57.0
GM-CSF	0	6.8 ± 3.9	8.2 ± 5.0	38.8 ± 91.0
	2	6.4 ± 3.0	8.0 ± 5.2	36.5 ± 84.2
	4	8.3 ± 4.4	9.0 ± 4.2	12.5 ± 16.5
	7	5.2 ± 2.8	8.8 ± 6.0	17.5 ± 20.3
VEGF	0	0.6 ± 0.5	0.7 ± 0.3	0.8 ± 0.7
	2	0.8 ± 0.7	0.8 ± 0.4	1.0 ± 1.0
	4	1.0 ± 0.5	0.9 ± 0.4	1.1 ± 0.6
	7	0.6 ± 0.3	0.9 ± 0.5	1.0 ± 0.4
EGF	0	21.5 ± 11.4	13.4 ± 6.1	17.1 ± 12.3
	2	18.0 ± 8.8	13.0 ± 6.6	14.0 ± 8.9
	4	18.0 ± 10.5	13.1 ± 6.3	13.6 ± 7.8
	7	18.6 ± 9.7	14.1 ± 7.6	16.4 ± 9.3
HGF	0	103.5 ± 67.2	73.7 ± 23.7	173.5 ± 274.4
	2	83.5 ± 55.2	46.9 ± 40.8	124.6 ± 161.5
	4	100.4 ± 74.8	60.1 ± 32.1	104.3 ± 102.4
	7	91.4 ± 82.6	81.5 ± 25.8	146.3 ± 141.5
FGF-2	0	14.4 ± 3.8	8.0 ± 2.9	12.7 ± 8.0
	2	12.9 ± 3.7	6.8 ± 3.6	10.7 ± 5.3
	4	12.1 ± 6.4	8.8 ± 4.2	10.6 ± 6.4
	7	11.3 ± 6.2	7.3 ± 4.6	12.5 ± 6.9
Eotaxin	0	178.6 ± 94.9	148.2 ± 49.2	138.7 ± 53.5
	2	125.4 ± 61.8	147.5 ± 59.0	204.4 ± 42.3
	4	135.4 ± 67.4	121.2 ± 36.4	141.8 ± 47.2
	7	123.3 ± 71.6	98.7 ± 34.9	147.7 ± 73.7

[a] MIP-1α, macrophage inflammatory cytokine-1 alpha; RANTES, Regulated upon Activation, Normal T Cell Expressed and Secreted; I-TAC, interferon-inducible T cell alpha chemoattractant; MDC, macrophage-derived chemokine; MIF, macrophage migration inhibitory factor; G-CSF, granulocyte colony stimulating factor; GM-CSF, granulocyte macrophage CSF; VEGF, vascular endothelial growth factor; EGF, epidermal growth factor; HGF, hepatocyte growth factor; FGF-2, fibroblast growth

Table S5. Spearman Correlation Data.

**Predictor**		**Spearman Correlation[1]**	
	**Coefficient (r)**	**Unadjusted p value**	**Adjusted p value**
Virus Replication in the Upper and Lower Respiratory Tract			
Lung Viral RNA			
Nasal Viral RNA (day 4)	0.312	0.1463	0.1688
Pharyngeal Viral RNA (day 4)	0.622	0.0016	0.0030
**Nasal Viral RNA (day 4)**			
Pharyngeal Viral RNA (day 4)	0.381	0.0743	0.0929
Lung Radiology Score vs. Virus Replication			
Radiology Score (Day 0 – Day 7)			
Lung Viral RNA	0.439	0.0334[a]	0.0456[a]
Nasal Viral RNA (day 4)	0.202	0.3517	0.3768
Pharyngeal Viral RNA (day 4)	0.638	0.0011	0.0024
Radiology Score (Day 7)			
Lung Viral RNA	0.475	0.0205	0.0308[a]
Nasal Viral RNA (day 4)	0.169	0.4376	0.4376
Pharyngeal Viral RNA (day 4)	0.765	<0.0001	0.0002
Radiograph Score (day 0- day 7)	0.930	<0.0001	<0.0001
Lung Pathology Score vs. Virus Replication			
Lung Viral RNA	0.770	<0.0001	0.0002
Nasal Viral RNA (day 4)	0.651	0.0010	0.0024
Pharyngeal Viral RNA (day 4)	0.810	<0.0001	<0.0001
Lung Pathology vs. Radiology Score			
Radiology Score (day 0- day 7)	0.577	0.0038	0.0063
Radiology Score (day 7)	0.643	0.0009	0.0024
**Correlates of Protection - wk 52 (Day 0)**			
IgG Binding Abs D614G			
Lung Radiology Score (day 0- day 7)	-0.501	0.0137	0.0256
Lung Radiology Score (day 7)	-0.585	0.0033	0.0097
Lung Pathology Score	-0.900	<0.0001	<0.0001
Lung Viral RNA (day 7)	-0.676	0.0004	0.0019
Nasal Viral RNA (day 4)	-0.573	0.0046	0.0114
Pharyngeal Viral RNA (day 4)	-0.882	<0.0001	<0.0001
IgG Binding Abs B.1.617.2			
Lung Radiology Score (day 0- day 7)	-0.565	0.0048	0.0116
Lung Radiology Score (day 7)	-0.636	0.0012	0.0097
Lung Pathology Score	-0.916	<0.0001	0.0040
Lung Viral RNA	-0.689	0.0003	0.0015
Nasal Viral RNA (day 4)	-0.546	0.0083	0.0178
Pharyngeal Viral RNA (day 4)	-0.884	<0.0001	<0.0001
Breadth Score			
Lung Radiology Score (day 0- day 7)	-0.603	0.0024	0.0074
Lung Radiology Score (day 7)	-0.668	0.0006	0.0023
Lung Pathology Score	-0.906	<0.0001	<0.0001
Lung Viral RNA	-0.694	0.0003	0.0014
Nasal Viral RNA (day 4)	-0.561	0.0058	0.0137
Pharyngeal Viral RNA (day 4)	-0.888	<0.0001	<0.0001
Nasal IgG B.1.617.2			
Lung Radiology Score (day 0- day 7)	-0.541	0.0072	0.0160
Lung Radiology Score (day 7)	-0.624	0.0015	0.0050
Lung Pathology Score	-0.889	<0.0001	<0.0001
Lung Viral RNA	-0.701	0.0002	0.0012
Nasal Viral RNA (day 4)	-0.554	0.0071	0.0160
Pharyngeal Viral RNA (day 4)	-0.861	<0.0001	<0.0001

Predictor		Spearman Correlation	
	Coefficient (r)	Unadjusted p value	Adjusted p value
Salivary IgG B.1.617.2			
Lung Radiology Score (day 0- day 7)	-0.524	0.0096	0.0196
Lung Radiology Score (day 7)	-0.572	0.0042	0.0110
Lung Pathology Score	-0.878	<0.0001	<0.0001
Lung Viral RNA	-0.770	<0.0001	0.0002
Nasal Viral RNA (day 4)	-0.519	0.0124	0.0239
Pharyngeal Viral RNA (day 4)	-0.854	<0.0001	<0.0001
Nasal IgA B.1.617.2			
Lung Radiology Score (day 0- day 7)	-0.641	0.0010	0.0037
Lung Radiology Score (day 7)	-0.655	0.0008	0.0029
Lung Pathology Score	-0.763	<0.0001	0.0001
Lung Viral RNA	-0.567	0.0046	0.0114
Nasal Viral RNA (day 4)	-0.363	0.0882	0.1343
Pharyngeal Viral RNA (day 4)	-0.708	<0.0001	0.0006
Salivary IgA B.1.617.2			
Lung Radiology Score (day 0- day 7)	-0.178	0.4016	0.4816
Lung Radiology Score (day 7)	-0.154	0.4718	0.5469
Lung Pathology Score	-0.392	0.0596	0.0965
Lung Viral RNA	-0.157	0.4611	0.5406
Nasal Viral RNA (day 4)	-0.316	0.1428	0.2051
Pharyngeal Viral RNA (day 4)	-0.402	0.0512	0.0842
Neutralizing Abs D614G			
Lung Radiology Score (day 0- day 7)	-0.534	0.0084	0.0178
Lung Radiology Score (day 7)	-0.609	0.0022	0.0069
Lung Pathology Score	-0.992	<0.0001	<0.0001
Lung Viral RNA	-0.770	<0.0001	0.0002
Nasal Viral RNA (day 4)	-0.586	0.0037	0.0101
Pharyngeal Viral RNA (day 4)	-0.832	<0.0001	<0.0001
Neutralizing Abs B.1.617.2			
Lung Radiology Score (day 0- day 7)	-0.502	0.0138	0.0256
Lung Radiology Score (day 7)	-0.592	0.0030	0.0090
Lung Pathology Score	-0.916	<0.0001	<0.0001
Lung Viral RNA	-0.732	0.0001	0.0006
Nasal Viral RNA (day 4)	-0.593	0.0036	0.0101
Pharyngeal Viral RNA (day 4)	-0.768	<0.0001	0.0002
Degranulation D614G			
Lung Radiology Score (day 0- day 7)	-0.085	0.7531	0.8002
Lung Radiology Score (day 7)	-0.158	0.5571	0.6313
Lung Pathology Score	-0.661	0.0067	0.0156
Lung Viral RNA	-0.474	0.0622	0.0991
Nasal Viral RNA (day 4)	-0.658	0.0098	0.0196
Pharyngeal Viral RNA (day 4)	-0.285	0.2794	0.3607
Degranulation B.1.617.2			
Lung Radiology Score (day 0- day 7)	0.074	0.7733	0.8131
Lung Radiology Score (day 7)	0.136	0.6253	0.6714
Lung Pathology Score	0.144	0.5915	0.6487
Lung Viral RNA	0.045	0.8585	0.8694
Nasal Viral RNA (day 4)	0.146	0.6019	0.6531
Pharyngeal Viral RNA (day 4)	-0.010	0.9703	0.9703

Predictor	Spearman Correlation Coefficient (r)	Unadjusted p value	Adjusted p value
CD4⁺ PBMC WT			
Lung Radiology Score (day 0- day 7)	-0.323	0.1231	0.1820
Lung Radiology Score (day 7)	-0.437	0.0341	0.0600[b]
Lung Pathology Score	-0.412	0.0465[a]	0.0791[a]
Lung Viral RNA	-0.578	0.0038	0.0101[a]
Nasal Viral RNA (day 4)	-0.039	0.8609	0.8694
Pharyngeal Viral RNA (day 4)	-0.445	0.0297[a]	0.0532
CD8⁺ PBMC WT			
Lung Radiology Score (day 0- day 7)	-0.205	0.3334	0.4198
Lung Radiology Score (day 7)	-0.288	0.1716	0.2431
Lung Pathology Score	-0.042	0.8479	0.8694
Lung Viral RNA	-0.201	0.3431	0.4268
Nasal Viral RNA (day 4)	0.172	0.4307	0.5108
Pharyngeal Viral RNA (day 4)	-0.181	0.3910	0.4748
CD4⁺ PBMC B.1.617.2			
Lung Radiology Score (day 0- day 7)	-0.454	0.0273	0.0497
Lung Radiology Score (day 7)	-0.509	0.0122	0.0239
Lung Pathology Score	-0.524	0.0095	0.0196
Lung Viral RNA	-0.360	0.0844	0.1305
Nasal Viral RNA (day 4)	-0.380	0.0737	0.1157
Pharyngeal Viral RNA (day 4)	-0.637	0.0011	0.0039
CD8⁺ PBMC B.1.617.2			
Lung Radiology Score (day 0- day 7)	-0.183	0.3904	0.4748
Lung Radiology Score (day 7)	-0.275	0.1929	0.2624
Lung Pathology Score	-0.275	0.1910	0.2624
Lung Viral RNA	-0.225	0.2884	0.3676
Nasal Viral RNA (day 4)	-0.266	0.2216	0.2930
Pharyngeal Viral RNA (day 4)	-0.315	0.1332	0.1941
**Correlates of Protection or Infection - Day 7**			
IgG Binding Abs D614G			
Lung Radiology Score (day 0- day 7)	-0.457	0.0261	0.0599
Lung Radiology Score (day 7)	-0.556	0.0056	0.0187
Lung Pathology Score	-0.855	<0.0001	<0.0001
Lung Viral RNA	-0.662	0.0006	0.0040
Nasal Viral RNA (day 4)	-0.484	0.0205	0.0506
Pharyngeal Viral RNA (day 4)	-0.782	<0.0001	0.0001
IgG Binding Abs B.1.617.2			
Lung Radiology Score (day 0- day 7)	-0.508	0.0124	0.0331
Lung Radiology Score (day 7)	-0.588	0.0031	0.0121
Lung Pathology Score	-0.879	<0.0001	<0.0001
Lung Viral RNA	-0.686	0.0003	0.0026
Nasal Viral RNA (day 4)	-0.470	0.0250	0.0594[b]
Pharyngeal Viral RNA (day 4)	-0.799	<0.0001	0.0002
Breadth Score			
Lung Radiology Score (day 0- day 7)	-0.524	0.0096	0.0289
Lung Radiology Score (day 7)	-0.598	0.0026	0.0108
Lung Pathology Score	-0.857	<0.0001	<0.0001
Lung Viral RNA	-0.581	0.0036	0.0132[a]
Nasal Viral RNA (day 4)	-0.599	0.0032	0.0121
Pharyngeal Viral RNA (day 4)	-0.817	<0.0001	<0.0001

Predictor	Coefficient (r)	Spearman Correlation<br>Unadjusted p value	Adjusted p value
Nasal IgG B.1.617.2			
Lung Radiology Score (day 0- day 7)	-0.470	0.0217	0.0527
Lung Radiology Score (day 7)	-0.562	0.0051	0.0175
Lung Pathology Score	-0.830	<0.0001	<0.0001
Lung Viral RNA	-0.652	0.0008	0.0047
Nasal Viral RNA (day 4)	-0.523	0.0113	0.0331
Pharyngeal Viral RNA (day 4)	-0.861	<0.0001	<0.0001
Salivary IgG B.1.617.2			
Lung Radiology Score (day 0- day 7)	-0.561	0.0051	0.0175
Lung Radiology Score (day 7)	-0.653	0.0008	0.0047
Lung Pathology Score	-0.856	<0.0001	<0.0001
Lung Viral RNA	-0.603	0.0023	0.0101[a]
Nasal Viral RNA (day 4)	-0.602	0.0027	0.0111
Pharyngeal Viral RNA (day 4)	-0.896	<0.0001	<0.0001
Nasal IgA B.1.617.2			
Lung Radiology Score (day 0- day 7)	-0.456	0.0264	0.0599
Lung Radiology Score (day 7)	-0.488	0.0167	0.0431[a]
Lung Pathology Score	-0.755	<0.0001	0.0003
Lung Viral RNA	-0.618	0.0017	0.0080[a]
Nasal Viral RNA (day 4)	-0.380	0.0749	0.1472
Pharyngeal Viral RNA (day 4)	-0.665	0.0006	0.0038
Salivary IgA B.1.617.2			
Lung Radiology Score (day 0- day 7)	-0.416	0.0438	0.0936
Lung Radiology Score (day 7)	-0.398	0.0556	0.1130
Lung Pathology Score	-0.640	0.0012	0.0065
Lung Viral RNA	-0.547	0.0066	0.0213
Nasal Viral RNA (day 4)	-0.241	0.2653	0.3556
Pharyngeal Viral RNA (day 4)	-0.616	0.0014	0.0076
Neutralizing Abs D614G			
Lung Radiology Score (day 0- day 7)	-0.455	0.0269	0.0599
Lung Radiology Score (day 7)	-0.510	0.0121	0.0331[a]
Lung Pathology Score	-0.810	<0.0001	<0.0001
Lung Viral RNA	-0.671	0.0005	0.0038[a]
Nasal Viral RNA (day 4)	-0.574	0.0043	0.0155
Pharyngeal Viral RNA (day 4)	-0.716	<0.0001	0.0009
Neutralizing Abs B.1.617.2			
Lung Radiology Score (day 0- day 7)	-0.321	0.1265	0.2140
Lung Radiology Score (day 7)	-0.346	0.0981	0.1791
Lung Pathology Score	-0.733	<0.0001	0.0007
Lung Viral RNA	-0.611	0.0020	0.0089
Nasal Viral RNA (day 4)	-0.635	0.0016	0.0078
Pharyngeal Viral RNA (day 4)	-0.623	0.0016	0.0078
Degranulation D614G			
Lung Radiology Score (day 0- day 7)	-0.292	0.1636	0.2546
Lung Radiology Score (day 7)	-0.360	0.0841	0.1558
Lung Pathology Score	-0.706	0.0001	0.0012
Lung Viral RNA	-0.366	0.0794	0.1494
Nasal Viral RNA (day 4)	-0.710	0.0003	0.0020
Pharyngeal Viral RNA (day 4)	-0.529	0.0083	0.0263[a]

Predictor	Spearman Correlation Coefficient (r)	Unadjusted p value	Adjusted p value
Degranulation B.1.617.2			
Lung Radiology Score (day 0- day 7)	0.152	0.4759	0.5501
Lung Radiology Score (day 7)	-0.027	0.9004	0.9331
Lung Pathology Score	-0.168	0.4348	0.5168
Lung Viral RNA	-0.026	0.9035	0.9331
Nasal Viral RNA (day 4)	0.012	0.9585	0.9585
Pharyngeal Viral RNA (day 4)	-0.211	0.3203	0.4031
$CD4^+$ PBMC WT			
Lung Radiology Score (day 0- day 7)	-0.072	0.7496	0.8004
Lung Radiology Score (day 7)	-0.130	0.5620	0.6379
Lung Pathology Score	-0.430	0.0468	0.0966
Lung Viral RNA	-0.240	0.2811	0.3689
Nasal Viral RNA (day 4)	-0.507	0.0204	0.0506
Pharyngeal Viral RNA (day 4)	-0.177	0.4278	0.5133
$CD8^+$ PBMC WT			
Lung Radiology Score (day 0- day 7)	0.357	0.1034	0.1835
Lung Radiology Score (day 7)	0.178	0.4257	0.5133
Lung Pathology Score	0.103	0.6451	0.7122
Lung Viral RNA	0.025	0.9141	0.9364
Nasal Viral RNA (day 4)	0.077	0.7417	0.7988
Pharyngeal Viral RNA (day 4)	0.110	0.6281	0.7004
$CD4^+$ PBMC B.1.617.2			
Lung Radiology Score (day 0- day 7)	0.015	0.9449	0.9533
Lung Radiology Score (day 7)	-0.155	0.4663	0.5441
Lung Pathology Score	-0.251	0.2364	0.3256
Lung Viral RNA	-0.210	0.3231	0.4031
Nasal Viral RNA (day 4)	-0.258	0.2344	0.3256
Pharyngeal Viral RNA (day 4)	-0.185	0.3866	0.4776
$CD8^+$ PBMC B.1.617.2			
Lung Radiology Score (day 0- day 7)	-0.058	0.7861	0.8324
Lung Radiology Score (day 7)	-0.229	0.2788[b]	0.3689
Lung Pathology Score	-0.312	0.1369	0.2231
Lung Viral RNA	-0.210	0.3209	0.4031
Nasal Viral RNA (day 4)	-0.045	0.8378	0.8796
Pharyngeal Viral RNA (day 4)	-0.312	0.1381	0.2231
$CD4^+$ Bronchial LN WT			
Lung Radiology Score (day 0- day 7)	0.252	0.2331	0.3256
Lung Radiology Score (day 7)	0.257	0.2239	0.3205
Lung Pathology Score	0.305	0.1467	0.2339
Lung Viral RNA	0.164	0.4421	0.5206
Nasal Viral RNA (day 4)	0.225	0.2990	0.3884
Pharyngeal Viral RNA (day 4)	0.273	0.1958	0.2902
$CD8^+$ Bronchial LN WT			
Lung Radiology Score (day 0- day 7)	0.296	0.1600	0.2520
Lung Radiology Score (day 7)	0.367	0.0781	0.1491
Lung Pathology Score	0.314	0.1369	0.2231
Lung Viral RNA	0.276	0.1912	0.2902
Nasal Viral RNA (day 4)	0.186	0.3922	0.4798
Pharyngeal Viral RNA (day 4)	0.249	0.2403	0.3256

Predictor	Spearman Correlation Coefficient (r)	Unadjusted p value	Adjusted p value
CD4⁺ Bronchial LN B.1.617.2			
Lung Radiology Score (day 0- day 7)	0.258	0.2212[b]	0.3204[b]
Lung Radiology Score (day 7)	0.334	0.1102[b]	0.1929[b]
Lung Pathology Score	0.414	0.0457	0.0959[b]
Lung Viral RNA	0.210	0.3209	0.4031
Nasal Viral RNA (day 4)	0.265	0.2211	0.3204
Pharyngeal Viral RNA (day 4)	0.319	0.1274	0.2140
CD8⁺ Bronchial LN B.1.617.2			
Lung Radiology Score (day 0- day 7)	0.370	0.0754[b]	0.1472
Lung Radiology Score (day 7)	0.509	0.0123	0.0331
Lung Pathology Score	0.451	0.0287	0.0623[b]
Lung Viral RNA	0.328	0.1180	0.2037
Nasal Viral RNA (day 4)	0.090	0.6823	0.7417
Pharyngeal Viral RNA (day 4)	0.345	0.0998	0.1796
CD4⁺ Bronchial LN N protein			
Lung Radiology Score (day 0- day 7)	0.454	0.0271	0.0599
Lung Radiology Score (day 7)	0.465	0.0152	0.0398
Lung Pathology Score	0.522	0.0096	0.0289[a]
Lung Viral RNA	0.370	0.0759	0.1472
Nasal Viral RNA (day 4)	0.107	0.6257	0.7004
Pharyngeal Viral RNA (day 4)	0.509	0.0116	0.0331[a]
CD8⁺ Bronchial LN N protein			
Lung Radiology Score (day 0- day 7)	0.249	0.2383	0.3256
Lung Radiology Score (day 7)	0.286	0.1738	0.2671
Lung Pathology Score	0.273	0.1937	0.2902
Lung Viral RNA	0.147	0.4906	0.5620
Nasal Viral RNA (day 4)	-0.015	0.9457	0.9533
Pharyngeal Viral RNA (day 4)	0.088	0.6829	0.7417
Antigen Correlations			
Plasma IgG (AUC)			
D614G vs. B.1.617.2	0.982	<0.0001	<0.0001
D614G vs. B.1.1.529	0.953	<0.0001	<0.0001
B.1.617.2 vs. B.1.1.529	0.974	<0.0001	<0.0001
CD4⁺ T PBMC			
WT vs. B.1.617.2	-0.037	0.8933	0.8933
WT vs. B.1.1.529	0.465	0.0718	0.0923
B.1.617.2 vs. B.1.1.529	0.483	0.0599	0.0899
CD8⁺ T PBMC			
WT vs. B.1.617.2	0.346	0.1887	0.2123
WT vs. B.1.1.529	0.624	0.0148	0.0334
B.1.617.2 vs. B.1.1.529	0.504	0.0582	0.0899

[1] We performed a second, sensitivity, analysis that excluded the three control animals that were housed outdoors prior to SARS-CoV-2 challenge in the correlation analysis to control for any potential impact a different host microbiota may have had on immune responses. In cases the correlation analysis with n=5 versus n=8 control rhesus macaques resulted in a change in p values that altered significance, we marked a loss of significance - defined as p<0.05 - with the letter “a” as superscript and a gain of significance – defined as p>0.05 - the letter “b” superscript.

Table S6. Lung Radiograph Scores

Group/ Animal	Day	Radiograph Score per Lobe: Right Lobes[a] Cran.	Mid.	Caud.	Access.[b]	Left Lobes[c] Cran./Cran.	Cran./Caud.	Caud.	Sum (day)	Total D1 to 7
**S Protein+ 3M-052-SE**										
RM1	0	0	0	0	0	0	0	0	0	
	2	0	0	0	0	0	0	0	0	
	4	0	1	0	0	0	0	0	1	
	7	0	0	0	0	0	0	0	0	**1**
RM2	0	0	0	0	0	0	0	0	0	
	2	0	0	0	0	0	0	0	0	
	4	0	0	0	0	0	0	0	0	
	7	0	0	0	0	0	0	0	0	**0**
RM3	0	0	0	0	0	0	0	0	0	
	2	0	0	0	0	0	0	0	0	
	4	0	1	0	0	0	0	0	1	
	7	0	0	0	0	0	0	0	0	**1**
RM4	0	0	0	0	0	0	0	0	0	
	2	0	0	0	0	0	0	0	0	
	4	0	0	0	0	0	0	0	0	
	7	0	0	0	0	0	0	0	0	**0**
RM5	0	0	0	0	0	0	0	0	0	
	2	0	0	0	0	0	0	0	0	
	4	0	0	0	0	0	0	0	0	
	7	0	0	0	0	0	0	0	0	**0**
RM6	0	0	0	0	0	0	0	0	0	
	2	0	0	0	0	0	0	0	0	
	4	0	0	0	0	0	0	0	0	
	7	0	0	0	0	0	0	0	0	**0**
RM7	0	0	0	0	0	0	0	0	0	
	2	0	0	0	0	0	0	0	0	
	4	0	0	0	0	0	0	0	0	
	7	0	1	0	0	0	0	0	1	**1**
RM8	0	0	0	0	0	0	0	0	0	
	2	0	0	0	0	0	0	0	0	
	4	0	0	0	0	0	0	0	0	
	7	0	1	0	0	0	0	0	1	**1**
**SARS-CoV-2 mRNA-LNP**										
RM9	0	0	0	0	0	0	0	0	0	
	2	0	0	0	0	1	0	0	1	
	4	0	0	0	0	0	0	0	0	
	7	0	0	0	0	0	0	0	0	**1**
RM10	0	0	0	0	0	0	0	0	0	
	2	0	0	0	0	0	0	0	0	
	4	0	0	0	0	0	0	0	0	
	7	0	0	0	0	0	0	0	0	**0**
RM11	0	0	0	0	0	0	0	0	0	
	2	0	0	1	0	0	0	0	1	
	4	0	0	2	0	0	0	0	2	
	7	0	1	1	0	0	0	0	2	**5**
RM12	0	0	0	0	0	0	0	0	0	
	2	0	0	0	0	0	0	0	0	
	4	0	0	0	0	0	0	0	0	
	7	0	0	0	0	0	0	0	0	**0**
RM13	0	0	0	0	0	0	0	0	0	
	2	0	0	0	0	0	0	0	0	
	4	0	0	0	0	0	0	0	0	
	7	0	0	0	0	0	0	0	0	**0**

Group/ Animal	Day	Radiograph Score per Lobe: Right Lobes[a]			Access.[b]	Left Lobes[c]			Sum	Total
		Cran.	Mid.	Caud.		Cran./Cran.	Cran./Caud.	Caud.	(day)	D1 to 7
**SARS-CoV-2 mRNA-LNP**										
RM14	0	0	0	0	0	0	0	0	0	
	2	0	1	0	0	0	0	0	1	
	4	0	0	0	0	0	1	0	1	
	7	1	0	0	0	1	0	0	2	**4**
RM15	0	0	0	0	0	0	0	0	0	
	2	0	1	0	0	0	1	0	1	
	4	0	0	0	0	0	0	1	1	
	7	1	0	0	0	0	1	0	2	**4**
RM16	0	0	0	0	0	0	0	0	0	
	2	0	0	0	0	0	0	0	0	
	4	0	0	0	0	0	0	0	0	
	7	0	0	0	0	0	0	0	0	**0**
**Control**										
RM17	0	0	0	0	0	0	0	0	0	
	2	1	0	0	0	1	0	0	2	
	4	1	0	0	0	1	1	0	3	
	7	1	0	0	0	1	1	0	3	**8**
RM18	0	0	0	0	0	0	0	0	0	
	2	0	0	0	0	0	0	0	0	
	4	0	0	0	0	0	0	0	0	
	7	0	0	0	0	0	0	0	0	**0**
RM19	0	0	0	0	0	0	0	0	0	
	2	1	0	0	0	1	0	0	2	
	4	1	1	0	1	1	0	0	4	
	7	2	1	2	1	1	1	0	8	**14**
RM20	0	0	0	0	0	0	0	0	0	
	2	0	0	0	0	1	0	0	1	
	4	1	0	0	0	0	0	0	1	
	7	0	0	0	0	1	1	0	2	**4**
RM21	0	0	0	0	0	0	0	0	0	
	2	1	1	0	0	1	0	0	3	
	4	1	0	0	0	1	0	0	2	
	7	1	1	0	0	1	1	0	4	**9**
RM22	0	0	0	0	0	0	0	0	0	
	2	0	0	0	0	0	0	0	0	
	4	0	0	0	0	0	0	0	0	
	7	0	0	0	0	0	0	0	0	**0**
RM23	0	0	0	0	0	0	0	0	0	
	2	2	0	2	0	0	0	1	5	
	4	0	0	0	0	0	0	0	0	
	7	1	0	1	0	0	0	1	3	**8**
RM24	0	0	0	0	0	0	0	0	0	
	2	0	1	0	0	0	0	0	1	
	4	1	0	0	0	0	0	0	1	
	7	1	1	0	0	0	1	0	3	**5**

[a] right lungs lobes include cranial (cran.), middle (mid.), and caudal (caud.) lobes
[b] accessory lobe
[c] left lungs lobes include cranial/cranial (cran./cran.), cranial/caudal (caran./caud.), and caudal (caud.) lobes

Table S7. Lung Pathology Scoring Criteria.

Score	Characteristics of Interstitial Pneumonia	Percent of Slides with Inflammation
**0**	**no interstitial pneumonia**<br>- alveolar parenchyma within normal limits	0%
**1**	**minimal interstitial pneumonia**<br>- rare small regions of mild alveolar interstitial expansion (1 to 2 cells thick)	<5%
**2**	**mild interstitial pneumonia**<br>- some regions of increased interstitial cellularity by inflammatory cells (up to 2 or 3 cells thick)<br>- usually with slightly increased numbers of alveolar macrophages	5% to 25%
**3**	**moderate interstitial pneumonia**<br>- moderate expansion of alveolar septae by inflammatory cells (with areas up to 4 to 6 cells thick)<br>- increased numbers of alveolar macrophages and sometimes alveolar neutrophils<br>- some cases of mild type 2 hyperplasia	25% to 50%
4	**severe interstitial pneumonia**<br>- marked increase in expansion of alveolar septae by inflammatory cells (with some areas more than 6 cells thick)<br>- increased numbers of alveolar macrophages and usually some alveolar neutrophils<br>- often type 2 hyperplasia<br>- some cases of alveolar edema or rare alveolar fibrin accumulation	>50%

Table S8. Lung Pathology Scores.

Group	Animal	Pathology Score						
		Caudal Lobe		Middle Lobe	Cranial Lobe	Lobe Average	Sum	Lung Average
		L	R	L	R			
**S Protein+ 3M-052-SE**	1	0.57	0.33	0.00	0.67	0.39	1.57	0.41
	2	0.29	0.25	0.00	0.00	0.14	0.54	0.20
	3	0.67	0.30	0.50	0.33	0.45	1.80	0.46
	4	0.67	0.63	0.00	0.67	0.49	1.97	0.58
	5	0.20	0.55	0.00	1.33	0.52	2.08	0.44
	6	0.22	0.42	0.00	0.00	0.16	0.64	0.25
	7	0.38	0.75	0.00	1.00	0.53	2.13	0.57
	8	0.38	1.13	0.00	0.43	0.49	1.94	0.58
**SARS-CoV-2 mRNA-LNP**	9	0.63	0.78	0.00	0.75	0.54	2.16	0.63
	10	0.67	0.89	0.67	1.75	1.00	3.98	0.95
	11	1.33	2.90	1.67	2.75	2.16	8.65	2.19
	12	0.67	0.63	0.50	0.67	0.62	2.47	0.63
	13	0.78	0.80	0.67	0.67	0.73	2.92	0.76
	14	0.86	1.00	0.50	0.75	0.78	3.11	0.80
	15	0.88	2.25	0.67	1.50	1.33	5.30	1.43
	16	0.78	1.10	0.67	0.67	0.81	3.22	0.83
**Control**	17	3.50	2.82	3.00	2.75	3.02	12.07	3.07
	18	2.22	2.55	3.00	1.67	2.36	9.44	2.30
	19	2.78	3.38	3.33	3.75	3.31	13.24	3.21
	20	1.29	1.00	1.00	1.00	1.07	4.29	1.09
	21	1.88	2.00	3.33	2.67	2.47	9.88	2.24
	22	3.13	2.43	2.33	4.00	2.97	11.89	2.82
	23	3.40	3.88	2.00	4.00	3.32	13.28	3.47
	24	2.56	2.78	4.00	4.00	3.34	13.34	2.96

Table S9. Data categorization into percentiles.

Parameter	Percentile		
	25th	50th	75th
**Immune Reponses**			
week 6			
Plasma D614G IgG (AUC)	9.83	14.13	15.45
D614G nAb (ID_{50} Titer)	5,095.50	11,325.00	49,971.80
Day of Challenge			
Plasma D614G IgG (AUC)	0.40	3.87	9.38
Plasma B.1.617.2 IgG (AUC)	0.45	3.71	9.85
Nasal B.1.617.2 RBD IgG (spec. act.)	0.00	0.09	0.42
Salivary B.1.617.2 RBD IgG (spec. act.)	0.00	0.14	0.60
Plasma IgG Breadth Score	0.00	14.70	17.70
D614G IgG-mediated Degranulation (AUC)	0.00	0.07	1.40
B.1.617.2 IgG-mediated Degranulation (AUC)	0.00	0.00	0.58
D614G nAb (ID_{50} Titer; pseudovirus)	20.0	72.50	429.20
B.1.617.2 nAb (ID_{50} Titer; pseudovirus)	20.0	88.50	447.80
B.1.617.2 nAb (ID_{50} Titer; whole virus)	1,299.25	3,135.00	4,571.00
WT S protein $CD4^+$ T cell responses (%)	0.48	1.06	1.82
B.1.617.2 S protein $CD4^+$ T cell responses (%)	0.69	1.20	1.82
WT S protein $CD8^+$ T cell responses (%)	0.70	0.95	1.30
B.1.617.2 S protein $CD8^+$ T cell responses (%)	0.60	1.01	1.59
**Challenge Outcome Parameters**			
Nasal SARS-CoV-2 N RNA (AUC)	1,154,276	78,239,648	390,786,722
Day 4 Nasal SARS-CoV-2 N RNA (copies/mL)	834	174,980	16,218,264
Pharyngeal SARS-CoV-2 N RNA (AUC)	220,438	834,021	40,410,389
Day 4 Pharyng. SARS-CoV-2 N RNA (copies/mL)	150	13,901	288,766
Day 4 BAL SARS-CoV-2 N RNA (copies/mL)	7,400	154,882	142,489,936
Lung SARS-CoV-2 N RNA (copies/30 mg tissue)	0.10	9.00	32,138
Clinical Score	2.00	4.00	6.25
Cage Observation Score	0.00	2.00	4.00
Average Lung Pathology Score	0.58	0.82	2.26
Day 2 Increase in Cytokines (fold-increase)[a]	1.09	1.58	4.80

[a] Only CXCL10 data were applied in this analysis because all 6 cytokines were highly correlated to each other.

Table S10. Antigens of the Binding Antibody Multiplex Assay.

**Protein**	**Vendor**	**Catalog Number**
S proteins		
S1+S2 of D614G	Sino Biological	40589-V08B4
Alpha (B.1.1.7)	Sino Biological	40589-V08B6
Beta (B.1.351)	Sino Biological	40589-V08B7
Gamma (P.1)	Sino Biological	40589-V08B8
Delta (B.1.617.2)	Sino Biological	40589-V08B16
Iota (B.1.562)	Sino Biological	40589-V08B21
Kappa (B.1.671.1)	Sino Biological	40589-V08B15
Delta+ S-protein	Sino Biological	40589-V08B26
Lambda (C.37)	Sino Biological	40589-V08B23
Eta (B.1.525)	Sino Biological	40589-V08B20
Omicron (B.1.1.529)	Sino Biological	40589-V08H26
Nucleoprotein		
N protein	Sino Biological	40589-V08B

Table S11. Antibodies for flow cytometric analysis of T cell responses.

Target	Clone	Fluorochrome[b]	Concentration [mg/mL]	Vendor	Catalog No.
Viability	N/A[a]	Aqua	N/A[a]	Invitrogen	L34966
CD3	SP34-2	APC-Cy7	2.000	BD Biosciences	557757
CD4	L200	PE-CF594	0.500	BD Biosciences	562402
CD8	RPA-T8	BV786	1.000	BD Biosciences	563823
CD45RA	5H9	V450	4.000	BD Biosciences	561220
CCR7	3D12	PE-Cy7	4.000	BD Biosciences	557648
IL-2	MQ1-17H12	PerCP-Cy5.5	0.125	BD Biosciences	560708
IL-17	eBio64CAP17	PE	8.000	eBiosciences	12-7178-42
IFN-γ	B27	AF700	0.120	BD Biosciences	557995
TNF-α	Mab11	APC	0.060	BD Biosciences	551384

[a] N/A, not applicable; [b] APC, allophycocyanin; PE, phycoerythrin; BV, brilliant violet; V, violet; PerCP, peridinin chlorophyll protein; AF, alexa fluor.

Data file S1. Raw, individual-level data.
