# Supplementary material for: Infant rhesus macaques immunized against SARS-CoV-2 are protected against heterologous virus challenge one year later
Source: Sci Transl Med. 2022 Dec 1;14:eadd6383. doi: 10.1126/scitranslmed.add6383 (PMC9765459; doi:10.1126/scitranslmed.add6383)
Supplement: Supplementary file 2 — MDAR Reproducibility Checklist [file scitranslmed.add6383_mdar_reproducibility_checklist.pdf]

Materials Design Analysis Reporting (MDAR)
Checklist for Authors

The MDAR framework establishes a minimum set of requirements in transparent reporting applicable to studies in the life sciences (see Statement of Task: doi:10.31222/osf.io/9sm4x.). The MDAR checklist is a tool for authors, editors, and others seeking to adopt the MDAR framework for transparent reporting in manuscripts and other outputs. Please refer to the MDAR Elaboration Document for additional context for the MDAR framework.

For all that apply, please note where in the manuscript the required information is provided.

Materials:

Newly created materials	indicate where provided: page no/section/legend)	n/a
The manuscript includes a dedicated "materials availability statement" providing transparent disclosure about availability of newly created materials including details on how materials can be accessed and describing any restrictions on access.	main manuscript page 31, lines 12-17 under "Data and Materials Availability"	

Antibodies	indicate where provided: page no/section/legend)	n/a
For commercial reagents, provide supplier name, catalogue number and RRID, if available.	main manuscript:<br>Materials and Methods Section, pages 18-21<br><br>Supplemental Material:<br>Materials and Methods Section, pages 5-11<br>Supplemental Tables S10 and S11	

DNA and RNA sequences	indicate where provided: page no/section/legend)	n/a
**Short novel DNA or RNA including primers, probes:** Sequences should be included or deposited in a public repository.	Primer sequences for SARS-CoV-2 RNA detection are listed in the Materials and Methods Section of Supplemental Materials, paragraph "SARS-CoV-2 RNA analysis" on page 10.	

Cell materials	indicate where provided: page no/section/legend	n/a
**Cell lines:** Provide species information, strain. Provide accession number in repository **OR** supplier name, catalog number, clone number, **OR** RRID.	Cell lines used to test for neutralization capacity of antibodies or for assessing antibody binding to spike protein expressing cells and antibody-mediated NK cell degranulations are listed in the main text in the in the Materials and Methods Section of Supplemental Materials, page 9	
**Primary cultures:** Provide species, strain, sex of origin, genetic modification status.	not applicable	x

Experimental animals	indicate where provided: page no/section/legend)	n/a
**Laboratory animals or Model organisms:** Provide species, strain, sex, age, genetic modification status. Provide accession number in repository **OR** supplier name, catalog number, clone number, **OR** RRID.	Rhesus macaques, male and female, were obtained from and housed at the CNPRC at UC Davis where all experimental animal procedures were performed. We used a total of n=24 rhesus macaques consisting of n=13 male and n=11 female animals.<br><br>Main manuscript:<br>Materials and Methods, paragraph "Animals": pages 18-19<br><br>Supplemental Material:<br>Table S1<br>Figure S8	
**Animal observed in or captured from the field:** Provide species, sex, and age where possible.	not applicable	n/a

Plants and microbes	indicate where provided: page no/section/legend)	n/a
**Plants:** provide species and strain, ecotype and cultivar where relevant, unique accession number if available, and source (including location for collected wild specimens).	not applicable	x
**Microbes:** provide species and strain, unique accession number if available, and source.	not applicable	x

Human research participants	indicate where provided: page no/section/legend) or state if these demographics were not collected	n/a
If collected and within the bounds of privacy constraints report on age, sex and gender or ethnicity for all study participants.	not applicable	x

Design:

Study protocol	indicate where provided: page no/section/legend)	n/a
If study protocol has been pre-registered, provide DOI. For clinical trials, provide the trial registration number **OR** cite DOI.	not applicable	x

Laboratory protocol	indicate where provided: page no/section/legend)	n/a
Provide DOI **OR** other citation details if detailed step-by-step protocols are available.	not applicable	x

Experimental study design (statistics details)		
**For in vivo studies:** State whether and how the following have been done	**indicate where provided: page no/section/legend. If it could have been done, but was not, write not done**	**n/a**
Sample size determination	Group sizes of n=8 were based on prior studies utilizing NHP models for SARS-CoV or MERS, that generally used 4-6 animals per experimental group. Furthermore, in our prior and ongoing pediatric HIV/ SIV vaccine studies a group size of n=6 to n=8 rhesus macaques has been sufficient to detect statistically significant differences in immune parameters between different vaccine regimens. In addition, group sizes were driven by the limited availability and capacity of BSL3 facilities necessary to conduct SARS-CoV-2 challenge studies.	
Randomisation	rhesus macaques were randomly assigned to the protein or mRNA vaccine group- see Materials and Methods, paragraph “Animals”, pages 18-19	
Blinding	Materials and Methods: Radiograph images (page 20) and lung histology (pages 10 to 11 in the Supplemental Materials) were assessed by a board-certified NHP veterinarian blinded to the assignment of the animal. Similarly, clinical observations were performed and recorded by veterinary personnel blinded to the study procedures.	
Inclusion/exclusion criteria	not applicable	x

Sample definition and in-laboratory replication	indicate where provided: page no/section/legend	n/a
State number of times the experiment was replicated in laboratory.	The animal study was performed only once.	
Define whether data describe technical or biological replicates.	Assay dependent; for each assay data set presented, we provide the number of animals per group (biological replicates) in the figure legend	

Ethics	indicate where provided: page no/section/legend	n/a

**Studies involving human participants:** State details of authority granting ethics approval (IRB or equivalent committee(s), provide reference number for approval.	not applicable	x
**Studies involving experimental animals:** State details of authority granting ethics approval (IRB or equivalent committee(s), provide reference number for approval.	Animal care followed the *Guide for Care and Use of Laboratory Animals* by the Institute for Laboratory Animal Research. Animal procedures were approved by the UC Davis Institutional Animal Care and Use Committee prior to study initiation, # 21820.<br>- see "Animals" in Materials and Methods: pages 18-19	
**Studies involving specimen and field samples:** State if relevant permits obtained, provide details of authority approving study; if none were required, explain why.	not applicable	x

Dual Use Research of Concern (DURC)	indicate where provided: page no/section/legend	n/a
If study is subject to dual use research of concern regulations, state the authority granting approval and reference number for the regulatory approval.	not applicable	x

Analysis:

Attrition	indicate where provided: page no/section/legend	n/a
Describe whether exclusion criteria were preestablished. Report if sample or data points were omitted from analysis. If yes report if this was due to attrition or intentional exclusion and provide justification.	Rhesus macaques had to be negative for type D retrovirus, simian immunodeficiency virus, simian lymphocyte tropic virus type 1 and SARS-CoV-2 to be enrolled in the study.<br>see “Animals” in Materials and Methods, pages 18-19	

Statistics	indicate where provided: page no/section/legend	n/a
Describe statistical tests used and justify choice of tests.	- main text: Materials and Methods – see “Statistical Analyses” on page 25<br>- if applicable, figure legends list statistical test applied to data<br>- Table S5 lists Spearman rank correlations	

Data availability	indicate where provided: page no/section/legend	n/a
For newly created and reused datasets, the manuscript includes a data availability statement that provides details for access or notes restrictions on access.	main manuscript: page 31 “Data and Materials Availability”	
If newly created datasets are publicly available, provide accession number in repository **OR** DOI **OR** URL and licensing details where available.	not applicable	
If reused data is publicly available provide accession number in repository **OR** DOI **OR** URL, **OR** citation.	not applicable	

Code availability	indicate where provided: page no/section/legend	n/a
For all newly generated custom computer code/software/mathematical algorithm or re-used code essential for replicating the main findings of the study, the manuscript includes a data availability statement that provides details for access or notes restrictions.	not applicable	X
If newly generated code is publicly available, provide accession number in repository, **OR** DOI **OR** URL and licensing details where available. State any restrictions on code availability or accessibility.	not applicable	x
If reused code is publicly available provide accession number in repository **OR** DOI **OR** URL, **OR** citation.	not applicable	x

Reporting

MDAR framework recommends adoption of discipline-specific guidelines, established and endorsed through community initiatives. Journals have their own policy about requiring specific guidelines and recommendations to complement MDAR.

Adherence to community standards	indicate where provided: page no/section/legend	n/a
State if relevant guidelines (e.g., ICMJE, MIBBI, ARRIVE) have been followed, and whether a checklist (e.g., CONSORT, PRISMA, ARRIVE) is provided with the manuscript.	We followed ARRIVE guidelines.<br>see page 30, line 32 under “Author Contributions”	
